# Cholinesterase Inhibition and Antioxidative Capacity of New Heteroaromatic Resveratrol Analogs: Synthesis and Physico—Chemical Properties

**DOI:** 10.3390/ijms25137401

**Published:** 2024-07-05

**Authors:** Milena Mlakić, Stanislava Talić, Ilijana Odak, Danijela Barić, Ivana Šagud, Irena Škorić

**Affiliations:** 1 Department of Organic Chemistry, Faculty of Chemical Engineering and Technology, University of Zagreb, Marulićev trg 19, HR-10000 Zagreb, Croatia; mdragojev@fkit.unizg.hr; 2 Department of Chemistry, Faculty of Science and Education, University of Mostar, Matice hrvatske bb, 88000 Mostar, Bosnia and Herzegovina; stanislava.talic@fpmoz.sum.ba (S.T.); ilijana.odak@fpmoz.sum.ba (I.O.); 3 Group for Computational Life Sciences, Division of Physical Chemistry, Ruđer Bošković Institute, Bijenička cesta 54, HR-10000 Zagreb, Croatia; dbaric@irb.hr; 4 Croatian Agency for Medicinal Products and Medical Devices, Ksaverska cesta 4, HR-10000 Zagreb, Croatia; ivana.sagud@halmed.hr

**Keywords:** ADME, antioxidative activity, cholinesterase inhibition, docking, genotoxicity, resveratrol, thiazole, thiophene

## Abstract

The targeted compounds in this research, resveratrol analogs **1**–**14**, were synthesized as mixtures of isomers by the Wittig reaction using heterocyclic triphenylphosphonium salts and various benzaldehydes. The planned compounds were those possessing the *trans*-configuration as the biologically active *trans*-resveratrol. The pure isomers were obtained by repeated column chromatography in various isolated yields depending on the heteroaromatic ring. It was found that butyrylcholinesterase (BChE) was more sensitive to the heteroaromatic resveratrol analogs than acetylcholinesterase (AChE), except for **6**, the methylated thiophene derivative with chlorine, which showed equal inhibition toward both enzymes. Compounds **5** and **8** achieved the highest BChE inhibition with IC_50_ values of 22.9 and 24.8 μM, respectively. The same as with AChE and BChE, methylated thiophene subunits of resveratrol analogs showed better enzyme inhibition than unmethylated ones. Two antioxidant spectrophotometric methods, DPPH and CUPRAC, were applied to determine the antioxidant potential of new heteroaromatic resveratrol analogs. The molecular docking of these compounds was conducted to visualize the ligand-active site complexes’ structure and identify the non-covalent interactions responsible for the complex’s stability, which influence the inhibitory potential. As ADME properties are crucial in developing drug product formulations, they have also been addressed in this work. The potential genotoxicity is evaluated by in silico studies for all compounds synthesized.

## 1. Introduction

Stilbenes, well-known conjugated compounds, are naturally occurring substances known for their diverse biological activity [1,2,3,4,5]. Hydroxy-stilbenes show particularly pronounced diverse therapeutic properties, primarily due to the presence of one or more hydroxyl groups responsible for strong antioxidant properties [6,7,8,9,10,11,12,13,14]. Antioxidants are generally considered substances whose properties make it possible to slow down or even prevent the oxidation of biological substrates caused by reactive substances such as free radicals [15,16]. As it is known, most polyphenols can cross the blood–brain barrier. Therefore, they are widely utilized in the treatment of various neurodegenerative diseases (ND). Resveratrol is a polyphenol and, at the same time, a hydroxy-stilbene; as such, it is a promising neuroprotective agent against neurodegenerative disorders such as Alzheimer’s disease (AD) due to its significant antioxidative behavior [17,18,19,20,21,22,23,24,25,26,27,28,29,30,31,32,33,34,35].

Acetylcholinesterase (AChE) and butyrylcholinesterase (BChE) are related enzymes that represent pharmacologically suitable targets in neurodegenerative disorders, due to their physiological roles in the body. AChE, responsible for the hydrolysis of the neurotransmitter acetylcholine (ACh), regulates ACh levels to support normal functioning in healthy organisms. In Alzheimer’s disease, the production of acetylcholine is diminished, so the inhibition of AChE helps maintain the normal levels of ACh. BChE is a serine hydrolase that also catalyzes the hydrolysis of ACh and participates in forming β-amyloid plaques characteristic of AD. Therefore, the inhibition of cholinesterases is a therapeutic strategy to slow AD progression. The treatment of neurodegenerative disorders currently includes common reversible cholinesterase enzyme inhibitors, such as galantamine, with proven efficacy in improving cognitive function. Like AChE, BChE deactivates the neurotransmitter acetylcholine—the level of BChE increases in the advanced stage of AD, so that is why BChE inhibitors can be particularly interesting. It is known that as AD progresses, the activity of AChE decreases (it falls to 33–45% of the usual normal activity). At the same time, the activity of BChE increases significantly (by 40 to 120% of the usual value). In the last few years, we have been designing and developing new potential cholinesterase inhibitors [36,37,38,39,40,41,42,43,44,45]. The synthesis was mainly performed by the Wittig reaction, photochemical electrocyclization, and triazole ring alkylation, where all three stages were very favorable with high yields.

**Figure 1:**
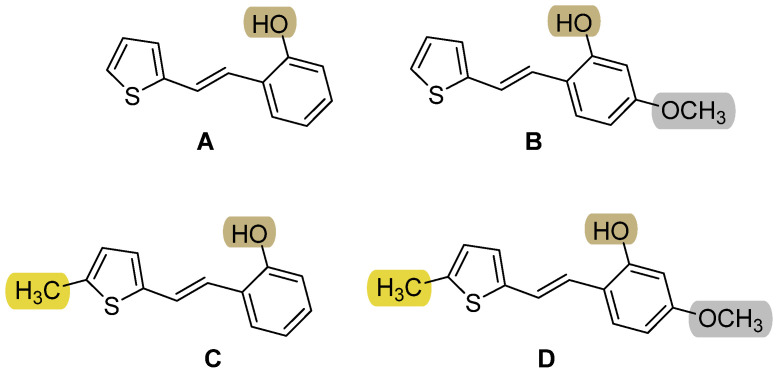
Previously confirmed potent BChE inhibitors and antioxidants [38,46].

In one of our last studies [46], new heterocyclic analogs based on the biologically active compound *trans*-resveratrol were prepared and isolated as *trans*-isomers. Biological testing on these resveratrol analogs showed that several of these compounds (Figure 1, compounds A–D) exhibited significantly enhanced BChE inhibitory and antioxidant activity. It is known that there are several hypotheses related to the primary cause of AD, and one of them is oxidative stress, which is a condition that occurs due to an imbalance between the formation of free radicals and the oxidative defense within cells. For this reason, the antioxidant potential of the molecules tested as cholinesterase inhibitors is very important. Molecular docking studies of selected ligands into BChE pointed out that the resveratrol analogs of interest displayed an affinity for forming hydrogen bonds with residues in the active site, which was accompanied by additional stabilizing effects such as π-π stacking and hydrophobic interactions.

As the continuation of the previous research, in the present work, new heteroaromatic analogs of resveratrol were prepared and spectroscopically characterized, and their potential for cholinesterase inhibition and antioxidation was analyzed. By introducing new substituents on the aromatic ring, and some old ones but at different positions on the aryl ring, it is planned to see which positions and which substituents contribute the most to biological activity and to see if this series of compounds will show any better results compared to the previous research [37,38,46]. The previous and latest results were compared, and conclusions were drawn about the relationship between the structure and activity of all heteroaromatic resveratrol analogs that have been prepared so far. The molecular docking of these compounds was planned to visualize the ligand-active site complexes’ structure and identify the non-covalent interactions responsible for the complex’s stability, which influence the inhibitory potential. In addition to testing the inhibition of the cholinesterase enzymes, the in silico ADMET properties of the new structures were tested, as they are crucial in developing drug-product formulations. The potential genotoxicity is also evaluated by in silico studies for all compounds synthesized.

## 2. Results and Discussion

### 2.1. Synthesis and Characterization of New Heteroaromatic Resveratrol Analogs ***1**–**14***

**Scheme 1:**
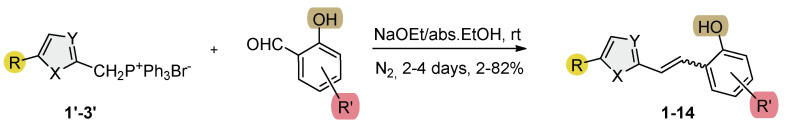
Synthesis of new resveratrol analogs **1**–**14** by Wittig reaction.

The target compounds **1**–**14** were synthesized as mixtures of isomers by the Wittig reaction using triphenylphosphonium salts and various benzaldehydes (Scheme 1) [47]. The Wittig reaction produced the mixture of two configurational isomers, each of 2-thieno- (**1**–**8**) or 1,3-thiazolo-stilbenes (**9**–**14**), with different ratios of *cis*- and *trans*-isomers depending on the substituents and especially on the nature of the heteroaromatic ring.

**Figure 2:**
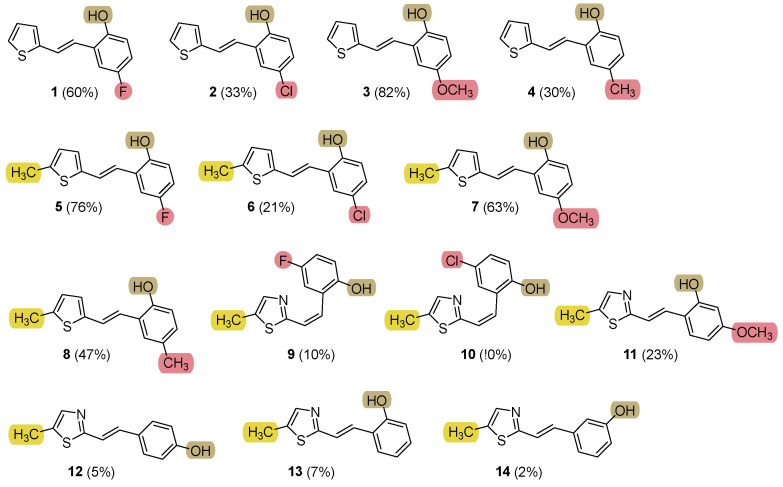
Structures of the synthesized resveratrol analogs **1**–**14** bearing various functionalities (isolated yields for individual compounds are shown in parentheses).

In the case of the unsubstituted thiophene salt **1′** (compounds **1**–**4**, Figure 2), the dominant *trans*-isomer is formed (the cis-isomer is represented by 10–20% of the reaction mixture). For the methyl-substituted thiophene salt **2′**, the proportion of the *cis*-isomer in the reaction mixture is even lower (5–10%, compounds **5**–**8**). In the case of thiazole salt **3′**, compared to the same aryl substituents, as with salt **1′**, the cis-isomer predominates (the *trans*-isomer represented 10–30%, compounds **9** and **10**). For the other thiazole derivatives (**11**–**14**), the *trans*-isomer is again predominant (the *cis*-isomer represents 10–20%), and the overall yields of the reactions are very low. The obtained mixtures of isomers were purified by extraction and column chromatography, where the petroleum ether/ether (PE/E) solvent system of variable proportions was used (see Section 3, Materials and Methods).

All the synthesized new phenolic stilbenes and resveratrol analogs **1**–**14** have been fully proven by NMR, UV, MS, and HRMS analyses (Figure 3, Figures S1–S75).

**Figure 4:**
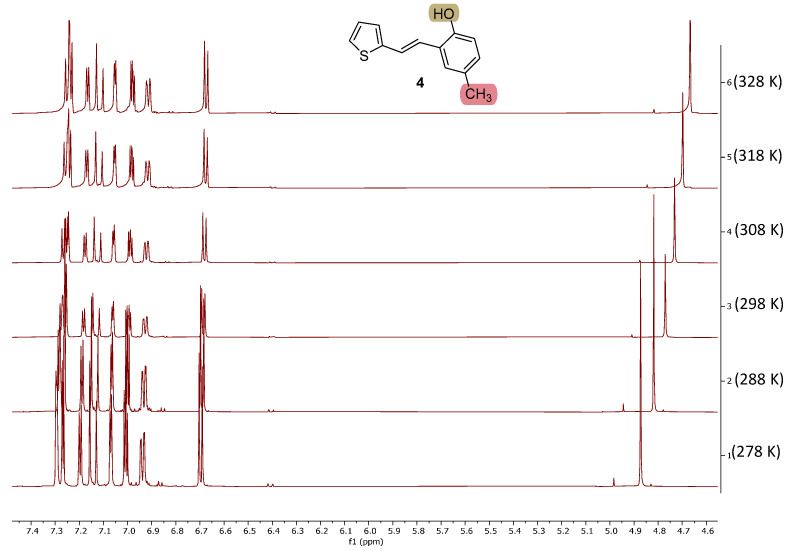
Temperature dependence of the chemical shifts of characteristic protons in the NMR experiments for compound **4**.

Resveratrol analog **4** was additionally exposed to temperature changes from 278 to 328 K. At each temperature, the corresponding ^1^H NMR spectrum was recorded (Figure 4) to examine possible changes in conformations due to the breaking of hydrogen bonds in the molecules. As expected, the largest change is shown by the acidic proton of the hydroxyl group (about 4.9 ppm) towards the more shaded area. In the unsaturated part of the molecule, the proton signals shift with increasing temperature towards the shielded area, but not with the same intensity. The largest shifts are shown by the aromatic proton in the immediate vicinity of the hydroxyl group and of the protons on the double bond, which in different conformations are both relatively close to the -OH group. 

This kind of examination of the change in chemical shifts under the temperature influence clearly shows that there are intermolecular hydrogen bonds between the molecules of resveratrol analogs in the solutions, which break, and the molecules become more flexible, taking some other conformations, which is reflected in the chemical shifts changes of individual protons that are closest to the particular changes.

### 2.2. Cholinesterase Inhibition and Antioxidative Potential of Resveratrol Analogs ***1**–**14***

**Figure 5:**
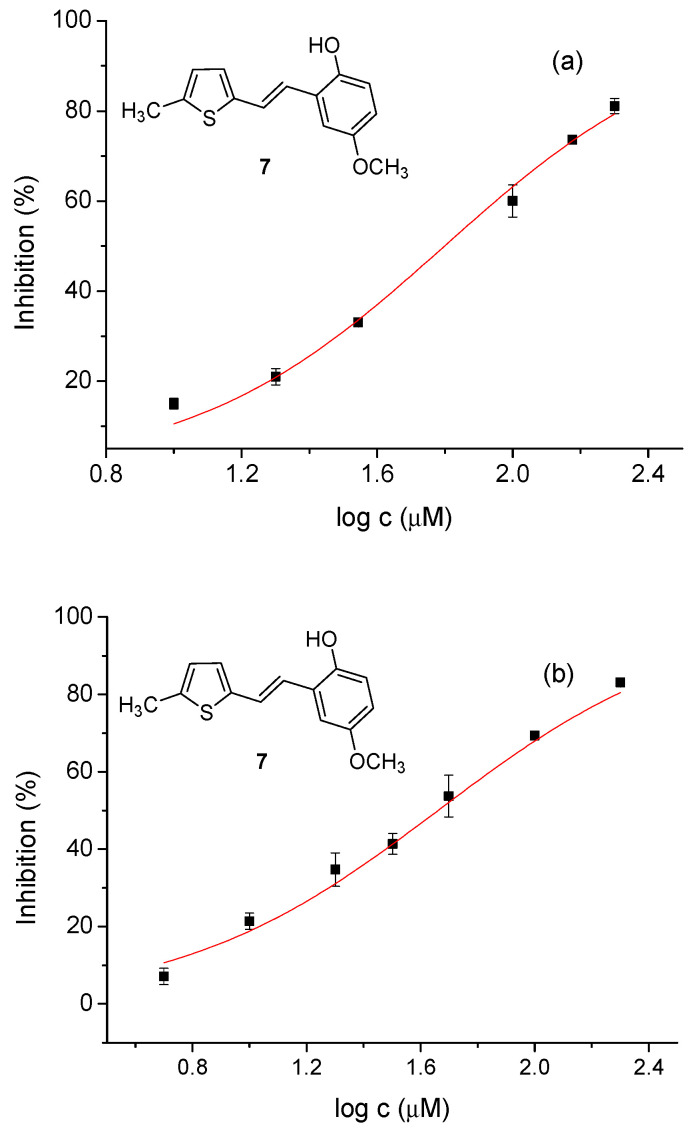
Dose-response curve for the inhibition of AChE (**a**) and BChE (**b**) by **7**.

**Figure 6:**
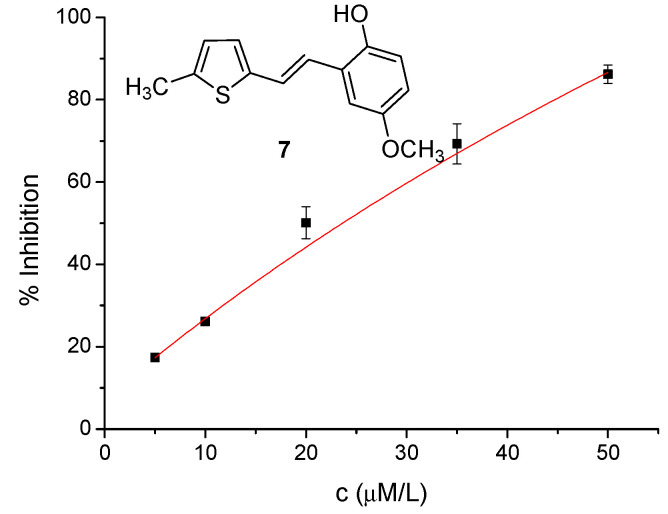

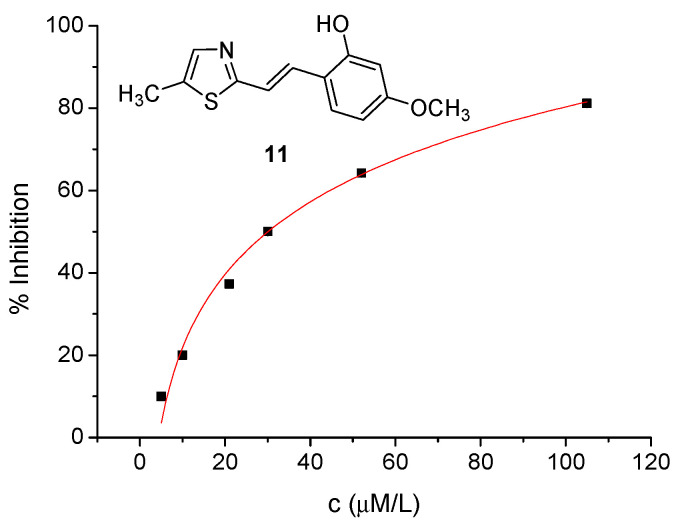
DPPH free radical scavenging activity of different concentrations of compounds **7** and **11**.

In our previous research [37,46], thiophene resveratrol analogues with OH groups in the ortho-position showed strong antioxidant activity. Some of them also showed high cholinesterase inhibition. The ortho-OH group in resveratrol analogs enables the formation of hydrogen bond(s) with amino acid residues within the enzymatic active site [46]. Therefore, these compounds were the starting point for the rational design of new bioactive resveratrol analogs with different substituents. Following synthesis and purification, the new heteroaromatic resveratrol analogs were tested for acetylcholinesterase (AChE) and butyrylcholinesterase (BChE) inhibition, as well as their antioxidant potential. In total, eight thiophene and six thiazole resveratrol analogues were investigated. The results of the investigation are summarized in Table 1. Figure 5 and Figure 6 are representative examples of dose-response curves for calculating IC_50_ values.

The inhibitory effect of new heteroaromatic resveratrol analogues on eeAChE and aqBChE enzymes was assayed using Ellman’s modified method [49]. The obtained results were compared with the reference galantamine. The influence of the type of heteroaromatic ring, the substituent on the heteroaromatic ring, and the influence of the type and position of the substituents on the phenol part was analysed.

Regarding the type of heterocyclic part of resveratrol analogs, it is obvious that the thiophene ring contributes to biological activity much more than the thiazole ring, since tiophene analogs achieved IC_50_ values in micromolar concentrations, while most analogues with a thiazole ring (**9**–**14**) did not show inhibitory activity. Among the thiophene analogs, the strongest AChE inhibitor was compound **6** (IC_50_ = 27.1 μM), with methyl substituent at the thiophene ring and *ortho*-OH and meta-Cl on the phenolic part. All investigated thiophenes showed AChE inhibition in the following order: **6** > **7** > **2** > **8** > **5** > **1** > **4** > **3**. It was observed that compounds **2** and **6** with a *meta*-Cl substituent on the polyphenolic ring inhibit AChE more strongly than compounds with fluoro, methyl, or methoxy substituents in the same position. In addition, all methylated thiophenes (**5**–**8**) showed better AChE inhibition than their unmethylated analogues (**1**–**4**). None of the investigated thiazoles achieved 50% inhibition of AChE.

In general, it was found that BChE was more sensitive to the heteroaromatic resveratrol analogs than AChE, except for **6,** which showed equal inhibition toward both enzymes. Compounds **5** and **8** achieved the highest BChE inhibition with IC_50_ values of 22.9 and 24.8 μM, respectively. With AChE and BChE, methylated thiophene subunits of resveratrol analogs showed better enzyme inhibition than unmethylated ones. A previous molecular docking study found that the methylated thiophene contributes to better binding via Trp82 at the active site of BChE [46]. In the total series of thiazole resveratrol analogs (**9**–**14**), only two compounds were selective BChE inhibitors, **12** and **13**. Both of them are methylated thiazoles with a monohydroxyl group in the *ortho* or *para* position. Thiazoles with a cis configuration and *meta*-halogen substituents did not reach 50% inhibition, nor did compounds **11** and **14**.

Two antioxidant spectrophotometric methods, DPPH and CUPRAC, were applied to determine the antioxidant potential of new heteroaromatic resveratrol analogs.

DPPH is the most commonly used method to determine the antioxidant activity of different potential antioxidants. DPPH is a stable organic radical capable of accepting an electron or a hydrogen radical and changing into a non-radical form, DPPH-H [50]. The percentage of radical quenching is used as a measure of the compound antioxidant capacity. For all new synthesized resveratrol analogs, the maximum inhibition percentages and their corresponding concentrations are also shown (Table 1).

The heteroaromatic resveratrol analogs with ortho-OH and electron-donating methoxy and methyl groups on the meta position of the phenol ring (**3**, **4**, **7**, **8**, **11**) showed stronger antioxidant activity than the standard resveratrol. Their IC_50_ values ranged from 23.8 to 51.4 μM in the following order: **7** > **8** > **11** > **3** > **4**. It was found that the methyl group on thiophene and thiazole rings has no effect on the antioxidant activity. The same was confirmed in our previous research [46]. The presence of halogen atoms on the phenol ring (**1**, **2**, **5**, **6**, **9**, **10**) decreased antioxidant activity. In the thiazole group, monohydroxylated *ortho* and *para*-OH (**12**, **13**) reach micromolar IC_50_ values, while their *meta*-OH analog (**14**) cannot be considered an antioxidant.

The CUPRAC test is based on the reduction of Cu^2+^ to Cu^+^ due to the action of antioxidants. A reaction occurs at a pH 7.0 (close to physiological pH) in the presence of neocuproin. The reactive Ar-OH groups of polyphenols and other antioxidants are oxidized to the corresponding quinones, and Cu^2+^-neocuproin is reduced to the colored complex Cu^+^-neocuproin [51]. The CUPRAC test confirmed the results from the DPPH method that the strongest antioxidants were **7** and **8**, followed by **6**, **3**, **1**, **4**, **2**, and **5**. In general, thiophene analogues of resveratrol proved to be more powerful antioxidants. Based on the results of the measurements, we can conclude that the compounds **7** and **8** have significant antioxidant activity, as well as the ability to inhibit cholinesterase.

### 2.3. ADME Properties of Resveratrol Analogs ***1**–**14***

Absorption, distribution, metabolism, excretion, and toxicity (ADME(T)) studies are critical in modern drug discovery. Critical concepts are divided into two areas: whether the compound exhibits drug-like pharmacokinetic properties and whether the compound has properties that will cause safety concerns in people [52]. ADMET properties are investigated both in silico, in vitro, and in vivo. For very early drug discovery, in silico tools are pivotal.

For this study, a free in silico tool [53] was used in order to screen the candidates that were experimentally shown to have potential as biologically active substances (Table 2, compounds **5**, **6**, **7,** and **8**).

The first step that a potential drug has to overcome is the absorption into the living human organism. Here, there are a few main obstacles: the solubility in water (linked to dissolution for the orally administered drug product) and the permeability and absorption in the intestines and or skin (depending on the route of administration). The solubility of all of these compounds is low, but this is not a rare occurrence with active drug substances and can be modified by appropriate drug-product formulations. As they have somewhat moderate permeability and a high HIA (human intestinal absorption), they are potentially good candidates for an oral route of administration.

The distribution and metabolism characterization show that all four molecules could have a fairly good distribution in the human body. The metabolites should be studied in detail, but the in silico preliminary results show them to be substrates only for the CYP3A4 enzyme, and as two of the compounds are also evaluated not to be hepatotoxic (**5** and **7**), they can be seen as the most promising candidates for advancement in the early development of drug products.

Genotoxicity has been evaluated in a separate chapter of this work.

### 2.4. Molecular Docking Study of Biologically Active Resveratrol Analogs

The measurement of cholinesterase inhibition for the studied resveratrol analogs indicated that compound **6** was the most effective candidate for AChE inhibition, with an IC_50_ of 27.1 μM. The most promising candidates for BChE inhibition were compounds **5** and **8**, with IC_50_ values of 22.9 and 24.8 μM, respectively. The molecular docking of these compounds was conducted to visualize the ligand-active site complexes’ structure and identify the non-covalent interactions responsible for the complex’s stability, which influence the inhibitory potential.

**Figure 7:**
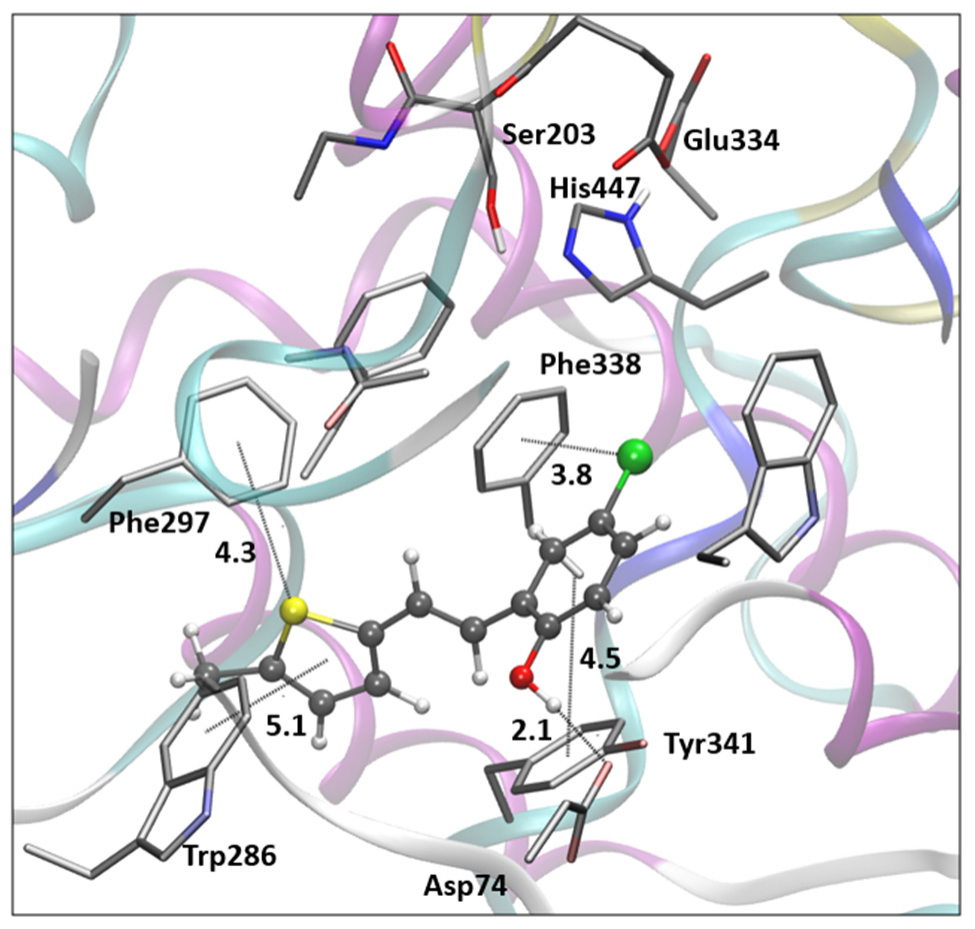
The structure of the complex obtained by docking molecule **6** into the active site of AChE.

The structure of the complex between molecule **6** and AChE (Figure 7) reveals a hydrogen bond between the hydroxyl group of the resveratrol fragment and the Asp74 residue located in the active site’s peripheral anionic subdomain (PAS). Other interactions are dispersive in nature: the thiophene sulfur interacts with the Phe297 residue of the acyl pocket subdomain, accompanied by π-π stacking between the aromatic thiophene moiety and Trp286. Another π-π stacking occurs between the phenyl ring of the resveratrol fragment and Tyr341 in PAS.

Additionally, the chlorine atom on the phenyl ring engages in a stabilizing dispersive interaction with Phe338, positioned 3.8 Å from the centroid of the phenyl ring and 3.4 Å from the closest carbon atom of the same residue (Figure S76). The difference between these distances, |Δd| = 0.4 Å, classifies this Cl-π interaction as “edge-on”; when |Δd| < 0.3 Å, the interaction is characterized as “face-on” [54] (Figure 7). The estimated free energy of binding for compound **6** in AChE is −8.28 kcal mol^−1^, compared to −11.37 kcal mol^−1^ for the reference compound donepezil, as shown in Table S1. Literature values for donepezil’s binding energy are similar, reported as −12.5, −11.9, and −10.8 kcal mol^−1^ [55,56,57].

**Figure 8:**
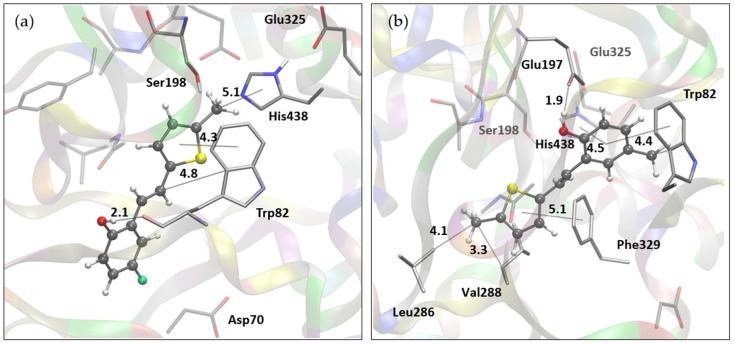
The structure of the active site of BChE docked with ligands: (**a**) molecule **5** and (**b**) molecule **8**.

The molecular docking of compounds **5** and **8** into the active site of BChE resulted in structures presented in Figure 8. In both cases, the hydroxyl group of resveratrol participates in the formation of a hydrogen bond (HB): in molecule **5**, the HB occurs between the -OH group in the ligand and the carbonyl group of Trp82 engaged in a peptide bond, whereas resveratrol’s -OH group in molecule **8** forms an HB with Glu197 of the anionic subdomain.

In the protein–ligand complex formed by docking molecule **5** into the active site of BChE, the methyl-substituted thiophene fragment is oriented toward the esteratic subdomain (catalytic triad: Ser198-His438-Glu325), enabling an alkyl-π interaction with His438 (Figure 8a). The thiophene aromatic core is involved in parallel π-π stacking with residue Trp82. An alkenyl-π dispersive interaction between the double CC bond in the ligand and Trp82 additionally stabilizes the complex.

In the most stable complex obtained by docking molecule **8** into the active site of BChE (Figure 8b), the ligand’s placement differs: His438 of the esteratic site is in non-covalent contact with the ligand’s phenyl ring. On the other side of the phenyl ring, π-π stacking with Trp82 is established; residue Trp82 is also engaged in an alkyl-π contact with the methyl substituent on the ligand’s phenyl. The thiophene fragment of molecule **8** is oriented towards the acyl pocket. Hence, the methyl substituent on thiophene participates in hydrophobic interactions with residues Leu286 and Val288, while the thiophene core engages in π-π stacking with residue Phe329. The estimated free energies of binding for compounds **5** and **8** in BChE are −7.26 and −7.09 kcal mol^−1^, compared to −9.58 kcal mol^−1^ for the reference compound donepezil (Table S2). This value for donepezil is slightly lower than the −10.6 kcal mol^−1^ reported in [56].

In summary of this section, the docking results indicate that all three resveratrol analogs with the most promising inhibitory potential toward cholinesterases can form hydrogen bonds with residues of the active site due to the -OH group on the phenyl ring. The two aromatic cores in each of these molecules, phenyl and thiophene, readily participate in π-π stacking with the aromatic residues of the active site. The methyl substituent on thiophene enables hydrophobic alkyl-π interactions in all three compounds, as does the methyl on the phenyl ring in molecule **8**. Additional dispersive stabilization is achieved in a complex containing compound **6** due to the presence of chlorine.

### 2.5. Genotoxicity of ***1**–**14***

Thorough investigation into all impurities that can be present in the active pharmaceutical compound (API) and the finished drug product is of critical importance for drug safety. Impurities that can be present in the active substance, as well as in each intermediate during the manufacturing process, have to be evaluated for toxicity (see Section 2.3. ADME properties of resveratrol analogs **1**–**14**), but an important subsection is their genotoxic potential, which is addressed in this section. Potential genotoxic compounds are more strictly regulated and controlled at much lower levels than other impurities (ICH M7 Guideline), and the levels that can be present in the drug substance/product are calculated on the basis of their determined acceptable daily intake (AI) and the maximum daily dose (MDD) of the final dosage form in question. With new compounds, the AI is usually not yet determined by toxicological studies on animals, and then the most conservative approach has to be taken with the strictest presumed AI as described in the guideline itself. Evaluations are primarily done always by the use of in silico Q(SAR) tools. When developing new active substances and finished drug forms, it is expected that the impurities will also be new compounds, and that there will be no experimental data available. In these cases, the Q(SAR) approach is of vital importance. (Q)SAR models make predictions of biological activity based on structural components [58]. (Q)SAR models are especially vital during the early stages of searching for potentially active drug substances. The elimination of all compounds can have mutagenic potential, which saves money and time. The most commonly used tool is the Lhasa software package (Nexus v.2.5.2 (Build 5, Jul 2022), Sarah Nexus v.3.2.1, Derek Nexus v.6.2.1) because it uses two complimentary models, and their predictions are then reviewed one more time by an expert.

In the case of compounds **1**–**14,** the resveratrol compound was used as a means to establish the nearest known compound that is used as a drug (Table 3). Derek Nexus found no structural alerts. For Sarah, some of these compounds are out of the scope. This is not surprising with very new synthetic compounds that are only starting to be investigated for their biological activity. This is a great example of how a complementary model is a must, especially in this early development. With compounds **8**, **9**, **11**, **13,** and **14,** Sarah Nexus has found a similar training set, and these compounds can be regarded as negative. They are the strongest candidates from the safety point of view as the safest ones for the continuation of the early stages of development. 

Compounds **1**, **3**, **4**, **5**, **7**, and **12** could be investigated further with some added scrutiny. If they become promising drug candidates, the in vitro AMES test [59] can then be done. For compounds **2**, **6**, and **10**, further in vitro AMES, and even in vivo qualification studies, would have to be done to show that they can be used as active drug substances. From the perspective of genotoxic safety, and the results that are presented for the biological activity measures, it seems that compound **8** is the most prospective lead.

## 3. Materials and Methods

### 3.1. General Remarks 

In the synthetic procedures of the target compounds, the following solvents were used (Sigma-Aldrich, St. Louis, MO, USA): petroleum ether (PE), diethyl ether (E), absolute ethanol (EtOH), dichloromethane (DCM), and carbon tetrachloride (CCl_4_). Toluene and DCM were used for extraction, and anhydrous MgSO_4_ was used as a drying salt for the organic layer after extraction. All solvents are commercially available and previously purified by distillation. Hydroxymethyl thiophene is purchased chemical. Rotary evaporator at reduced pressure was used to remove solvents from solutions. A column filled with silica gel (0.063–0.2 nm and 60 Å, technical grade, Fluka Chemie GmbH, Buchs, Switzerland) was used for column chromatography, and the PE/E system in different ratios was used as the mobile phase for its performance. Thin-layer chromatography was performed on plates coated with silica gel (0.2 mm; Kiselgel 60 F_254_) with the mobile phase of the PE/E system in different ratios. Compounds were detected on TLC plates with UV lamps at 254 nm and 365 nm. To prepare the samples for nuclear magnetic resonance (NMR), they were dissolved in deuterated chloroform (CDCl_3_) and imaged with tetramethylsilane (TMS) as an internal standard (Sigma-Aldrich, St. Louis, MO, USA). ^1^H NMR and ^13^C NMR techniques were used to confirm the structure of the synthesized compounds, and the spectra were recorded on a Bruker Avance instrument at 300 and 600 MHz for ^1^H NMR and at 75 and 150 MHz for ^13^C NMR. Chemical shifts (*δ*) are expressed in parts-per-million values (ppm) and coupling constants (*J*) in Hz.

### 3.2. Synthesis of Phosphonium Salts ***1′**–**3′***

In a 250 mL three-necked flask, 7.9 g (0.063 mol) of hydroxymethyl thiophene were dissolved in 72.3 mL of dry E and added to a three-necked flask. Then, 5.71 g (0.022 mol) of phosphorus tribromide were dissolved in 7.23 mL of dry E and added dropwise over 40 min into the reaction flask. The reaction mixture is stirred for 1 h at room temperature on a magnetic stirrer. After that, 15 mL of methanol and 100 mL of distilled water are added to the reaction mixture, and the extraction is carried out with a total of 300 mL of E. The ether layer was dried over anhydrous MgSO_4_ overnight and filtered, then evaporated to dryness on a rotary evaporator. After evaporation, bromomethyl thiophene remains in the flask as a yellow oil. The bromomethyl thiophene is dissolved in 20 mL of toluene and 16.52 g (0.037 mol) of triphenylphosphine, PPh_3_ was dissolved in 50 mL of toluene, and they were added to the reaction flask. The reaction mixture was stirred on a magnetic stirrer for 3 days and then filtered through a Büchner funnel under reduced pressure. The light-yellow salt obtained is dried in a desiccator for 12 h. Dry thiophene-phosphonium salt **1′** is used in all subsequent experiments to synthesize compounds **1**–**4**.

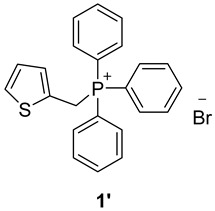



In a 250 mL round flask, 4.925 g of dimethylthiophene (0.0440 mol) were dissolved in 133 mL of carbon tetrachloride, CCl_4_. Then, 7.83 g of *N*-bromosuccinimide, NBS (1 eq.) were added to the reaction mixture. Chemicals were stirred in an oil bath at reflux temperature (150 °C). After the reflux started, the first batch of *α,α*-azobisisobutyronitrile, AIBN catalyst was added to the top of the spatula. The temperature of the oil bath was then reduced to 100 °C due to excessive reflux. When the reflux was established, the reaction mixture was illuminated with a lamp that initiated the reaction. After 1.5 h, the formation of the corresponding bromide began with the simultaneous separation of succinimide on the surface in the form of white fluffy crystals. When another hour had passed, the second dose of AIBN was added. The mixture was then refluxed for 3 h. The resulting succinimide was then filtered through pleated filter paper, and the resulting clear orange liquid was evaporated on a rotary evaporator. A brown oily precipitate of the resulting 2-bromomethyl-5-methyl thiophene remained in the flask. In the second step, thiophene bromide was dissolved in 10 mL of benzene with the addition of PPh_3_. The reaction mixture was heated to reflux (80 °C—mixer set to 110 °C) and stirred at that temperature for 1 h. Then the heating was turned off, and the mixture was stirred at room temperature for the next 24 h. The resulting salt was filtered using a Büchner funnel, the crystallizer was previously weighed, and then the salt was put into it and weighed again. The 2-methylthiophene salt **2’** was dried in a desiccator under vacuum for 4–5 h and, after that, used to synthesize compounds **5**–**8**.

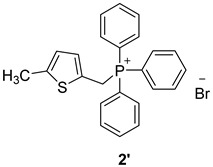



In the three-necked flask (500 mL), 110 mL of CCl_4_ and 8.65 g of NBS were added. An oil bath was placed on a magnetic stirrer, into which a round-bottomed flask was then immersed. Then, 5.5 g of the reactant 2,5-dimethyl-1,3-thiazole were added to the flask, after which the reaction mixture was stirred until it was refluxed at about 100–120 °C. A small amount of AIBN was added as a catalyst. The reaction mixture continued to stir while reflux was established, while it was illuminated by a lamp whose light stimulates the reaction. In the beginning, the mixture was orange in color. After approximately 1 h, 50 mg of AIBN were added and the mixture continued to be stirred at reflux at a temperature of 100–150 °C. During mixing, a white trace of NBS was observed on the surface of the flask, and the color of the reaction mixture changed from orange to dark brown. After a total of 3 h of mixing, the reaction mixture was filtered into a 250 mL round flask, and the solvent was evaporated from the flask. After the solvent was evaporated, 40 mL of toluene and 20 mL of toluene with 12.75 g of dissolved PPh_3_ were added to the flask with the 2-bromomethyl-5-methyl thiazole. The mixture was then heated to reflux and stirred for another 1 h at reflux. If necessary, a little more (10 mL) toluene was added for easier mixing of the dense reaction mixture. The mixture was then filtered, and the salt was left for 6 days in a desiccator to dry. The dry 2-methylthiazole salt **3’** was used to synthesize resveratrol analogs **9**–**14**.

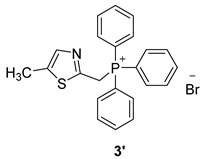



### 3.3. Synthesis of New Thienostilbenes ***1**–**8***

Before starting the reaction, the assembled apparatus was purged with nitrogen for 15 min. Then, 50 mL of absolute EtOH were added to the dropping funnel, 40 mL of which were dropped into a three-necked flask. Then, the calculated mass of the phosphonium salts **1′** (for compounds **1**–**4**) or **2′** (for compounds **5**–**8**) was added to the flask in a ratio of 1:1 in relation to 0.5 g of the corresponding aldehyde for the synthesis of compounds **1**–**8**. The reaction mixture was stirred on a magnetic stirrer at room temperature. After the salts **1′** or **2′** were dissolved, the calculated mass of sodium was slowly added to the addition funnel with EtOH already present to form sodium ethoxide (10 mL). A few drops of the resulting solution were dropped from the funnel into a three-necked flask to make the solution alkaline, and then a certain amount of aldehyde was added to the reaction flask. The remaining sodium ethoxide solution was slowly added dropwise from the funnel into the reaction mixture. The mixture was left to stir (1–94 h, 2–72 h, 3–48 h, 4–72 h, 5–65 h, 6–65 h, 7–48 h, 8–48 h) at room temperature, and the reaction was monitored by TLC plates in the PE/E system.

After completion of the reaction, the solvent was evaporated on a rotary evaporator under reduced pressure. The remaining residue was dissolved in 40 mL of solvent (**1**–**6** extractions with DCM, for seven and eight extractions with toluene), and 40 mL of distilled water were poured into a separatory funnel. The reaction mixture was extracted with corresponding solvent three times, after which the organic layer was dried over anhydrous MgSO_4_. The mixture was filtered into a round flask, and the solvent was evaporated on a rotary evaporator under reduced pressure. Compounds **1**–**8** were obtained as a mixture of *cis*- and *trans*-isomers, with a higher proportion of the *trans*-isomer. Products **1**–**8** were isolated by column chromatography on silica gel with a PE/E solvent system (1: 0–30%, 2, 8: 0–20%, 3, 4: 0–40% and 5–7: 0–60%). For all products, the structure was confirmed by spectroscopic methods, and their spectroscopic characterization is described below.

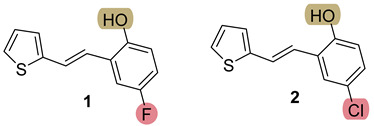



**4-fluoro-2-(2-(thiophen-2-yl)vinyl)phenol (1):** Following mixtures of *cis*- and *trans*-isomers with different ratios were obtained after the first column chromatography according to proton NMR: 260 mg (*trans*-:*cis*-:aldehyde = 2:1:7), 21 mg (*trans*-:*cis*- = 1:2), 34 mg (*trans*-:*cis*- = 4:1), and 59 mg (*trans*-:*cis*- = 9:1). The fraction with 59 mg (*trans*-:*cis*- = 9:1) in the next column chromatography with a PE/E solvent system (0–30%) provided 35 mg of pure *trans*-isomer.

**(*E*)-4-fluoro-2-(2-(thiophen-2-yl)vinyl)phenol (1):** 35 mg (isolated 60%), white powder; m.p. 92–93 °C; *Rf* (PE/E = 30%) = 0.78; ^1^H NMR (CDCl_3_, 300 MHz) *δ*/ppm: 7.23–7.21 (m, 2H), 7.17 (dd, *J* = 9.5, 2.9 Hz, 1H), 7.13–7.09 (m, 2H), 7.01 (dd, *J* = 5.1, 3.6 Hz, 1H), 6.48–6.81 (m, 1H), 6.74–6.72 (m, 1H), 4.81 (s, 1H); ^13^C NMR (CDCl_3_, 75 MHz) *δ*/ppm: 157.0 (d, *J*_C-F_ = 238 Hz), 148.9, 142.7, 127.6, 126.6, 125.6, 124.9, 124.2, 121.6, 116.9, 114.8 (d, *J*_C-F_ = 23.3 Hz), 112.7 (d, *J*_C-F_ = 23.3 Hz); MS (ESI) *m*/*z* (%, fragment): 219 (100); HRMS (*m*/*z*) for C_12_H_9_FOS: [M + H]^+^_calcd_ = 220.0358, and [M + H]^+^_measured_ = 220.0353.

**4-chloro-2-(2-(thiophen-2-yl)vinyl)phenol (2):** Column chromatography with a PE/E solvent system (0–20%) yielded 18 mg of the *trans*-isomer after repeated column chromatography. Mixtures of *cis*- and *trans*-isomers with different ratios were first obtained by column chromatography: 84 mg (*trans*-:*cis*-:aldehyde = 2.5:1:0.5), 94 mg (*trans*-:*cis*-:aldehyde = 1:0.3:0.1), 158 mg (*trans*-:*cis*-:aldehyde = 1:0.2:0.7), 74 mg (*trans*-:*cis*- = 2.5:1) and 55 mg (*trans*-:*cis*- = 5:1). The fraction with 55 mg (*trans*-:*cis*- = 5:1) in another column chromatography gave 18 mg of pure *trans*-isomer.

**(*E*)-4-chloro-2-(2-(thiophen-2-yl)vinyl)phenol (2):** 18 mg (isolated 33%), white powder; m.p. 89–91 °C; *Rf* (PE/E = 50%) = 0.61; ^1^H NMR (CDCl_3_, 600 MHz) *δ*/ppm: 7.43 (d, *J* = 2.5 Hz, 1H), 7.28–7.18 (m, 2H), 7.08 (d, *J* = 3.2 Hz, 2H), 7.05–6.98 (m, 2H), 6.71 (d, *J* = 8.9 Hz, 1H), 5.12 (s, 1H); ^13^C NMR (CDCl_3_, 75 MHz) *δ*/ppm: 151.4, 142.7, 128.0, 127.6, 126.4, 126.6, 126.1, 125.9, 124.9, 124.3, 121.3, 117.2; MS (ESI) *m*/*z* (%, fragment): 235 (100); HRMS (*m*/*z*) for C_12_H_9_ClOS: [M + H]^+^_calcd_ = 236.0063, and [M + H]^+^_measured_ = 236.0057.

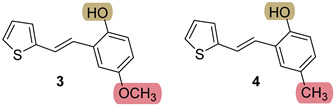



**4-methoxy-2-(2-(thiophen-2-yl)vinyl)phenol (3):** Column chromatography with a PE/E solvent system (0–40%) yielded 107 mg of the *trans*-isomer after repeated column chromatography. Mixtures of *cis*- and *trans*-isomers with different ratios were first obtained by column chromatography: 171 mg (*trans*-:*cis*- = 8.5:1), 42 mg (*trans*-:*cis*- = 8.5:1), 41 mg (*trans*-:*cis*- = 9:1), 130 mg (*trans*-:*cis*- = 17:1) and 32 mg (*trans*-:*cis*- = 23:1). The fraction with 130 mg (*trans*-:*cis*- = 17:1) in another column chromatography provides 107 mg of pure *trans*-isomer.

**(*E*)-4-methoxy-2-(2-(thiophen-2-yl)vinyl)phenol (3):** 107 mg (isolated 82%), yellow powder; m.p. 115–117 °C; *Rf* (PE/E = 30%) = 0.49; ^1^H NMR (CDCl_3_, 300 MHz) *δ*/ppm: 7.24 (d, *J* = 16.4 Hz, 1H), 7.17 (d, *J* = 5.2 Hz, 1H), 7.14 (d, *J* = 16.4 Hz, 1H), 7.05 (d, *J* = 3.7 Hz, 1H), 6.99 (d, *J* = 2.5 Hz, 1H), 6.98 (d, *J* = 5.0, 3.3 Hz, 1H), 6.71–6.67 (m, 2H), 5.17 (s, 1H), 3.78 (s, 3H); ^13^C NMR (CDCl_3_, 75 MHz) *δ*/ppm: 152.8, 146.2, 142.1, 126.5, 125.1, 124.0, 123.4, 122.2, 121.7, 115.9, 113.5, 110.5, 54.8; MS (ESI) *m*/*z* (%, fragment): 231 (100); HRMS (*m*/*z*) for C_13_H_12_O_2_S: [M + H]^+^_calcd_ = 232.0558, and [M + H]^+^_measured_ = 232.0551.

**4-methyl-2-(2-(thiophen-2-yl)vinyl)phenol (4):** Column chromatography with a PE/E solvent system (0–40%) yielded 101 mg of the pure *trans*-isomer after repeated column chromatography. Mixtures of *cis*- and *trans*-isomers with different ratios were first obtained: 48 mg (*trans*-:*cis*- = 5:1), 121 mg (*trans*-:*cis*- = 8:1), 53 mg (*trans*-:*cis*- = 11:1), 37 mg (*trans*-:*cis*- = 12:1) and 108 mg (*trans*-isomer mainly). 

**(*E*)-4-methyl-2-(2-(thiophen-2-yl)vinyl)phenol (4):** 101 mg (isolated 30%), yellow powder; m.p. 105–107 °C; *Rf* (PE/E = 30%) = 0.57; ^1^H NMR (CDCl_3_, 300 MHz) *δ*/ppm: 7.28–7.24 (m, 2H), 7.18 (d, *J* = 5.0 Hz, 1H), 7.13 (d, *J* = 16.1 Hz, 1H), 7.06 (d, *J* = 3.2 Hz, 1H), 6.98 (dd, *J* = 5.1, 3.7 Hz,1H), 6.92 (dd, *J* = 8.1, 1.6 Hz, 1H), 6.68 (d, *J* = 8.2 Hz, 1H), 4.89 (s, 1H), 2.28 (s, 3H); ^13^C NMR (CDCl_3_, 75 MHz) *δ*/ppm: 150.8, 143.4, 130.3, 129.2, 127.5, 125.8, 124.2, 123.9, 122.9, 122.9, 115.9, 20.6; MS (ESI) *m*/*z* (%, fragment): 215 (100); HRMS (*m*/*z*) for C_13_H_12_OS: [M + H]^+^_calcd_ = 216.0609, and [M + H]^+^_measured_ = 216.0609.

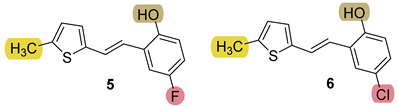



**4-fluoro-2-(2-(5-methylthiophen-2-yl)vinyl)phenol (5):** Column chromatography with a PE/E solvent system (0–60%) yielded 175 mg of pure *trans*-isomer. Mixtures of *cis*- and *trans*-isomers with different ratios were first obtained on column chromatography: 187 mg (*trans*-isomer mainly), 64 mg (*trans*-:*cis*- = 9:1), 46 mg (*trans*-:phosphine oxide = 10:1).

**(*E*)-4-fluoro-2-(2-(5-methylthiophen-2-yl)vinyl)phenol (5):** 175 mg (isolated yield 76%), white powder; m.p. 96–97 °C; *Rf* (PE/E = 50%) = 0.60; ^1^H NMR (CDCl_3_, 600 MHz) *δ*/ppm: 7.16–7.13 (m, 2H), 6.96 (d, *J* = 16.2 Hz, 1H), 6.86 (d, *J* = 3.7 Hz, 1H), 6.81–6.77 (m, 1H), 6.70 (dd, *J* = 8.2, 4.6 Hz, 1H), 6.65–6.64 (m, 1H), 4.84 (s, 1H), 2.50 (s, 3H); ^13^C NMR (CDCl_3_, 75 MHz) *δ*/ppm: 157.0 (d, *J*_C-F_ = 235.0 Hz), 148.8, 140.7, 139.9, 126.9, 125.8, 124.9, 124.6, 120.3, 116.8, 114.5 (d, *J*_C-F_ = 23.9 Hz), 112.6 (d, *J*_C-F_ = 23.9 Hz), 15.7; MS (ESI) *m*/*z* (%, fragment): 233 (100); HRMS (*m*/*z*) for C_13_H_11_FOS: [M + H]^+^_calcd_ = 234.0515, and [M + H]^+^_measured_ = 234.0515.

**4-chloro-2-(2-(5-methylthiophen-2-yl)vinyl)phenol (6):** Column chromatography with a PE/E solvent system (0–60%) yielded 47 mg of the pure *trans*-isomer. Mixtures of *cis*- and *trans*-isomers with different ratios were obtained directly from the first column chromatography: 141 mg (*trans*-:*cis*- = 12:1), 47 mg (pure *trans*-isomer), 38 mg (*trans*-:*cis*- = 8:2).

**(*E*)-4-chloro-2-(2-(5-methylthiophen-2-yl)vinyl)phenol (6):** 47 mg (isolated 21%), white powder; m.p. 90–91 °C; *Rf* (PE/E = 30%) = 0.56; ^1^H NMR (CDCl_3_, 300 MHz) *δ*/ppm: 7.41 (d, *J* = 2.6 Hz, 1H), 7.17 (d, *J* = 16.2 Hz, 1H), 7.05 (dd, *J* = 8.5, 2.6 Hz, 1H), 6.92 (d, *J* = 16.2 Hz, 1H), 6.87 (d, *J* = 3.6 Hz, 1H), 6.72 (d, *J* = 8.4 Hz, 1H), 6.64 (d, *J* = 2.7 Hz, 1H), 5.0 (s, 1H), 2.49 (s, 3H); ^13^C NMR (CDCl_3_, 75 MHz) *δ*/ppm: 151.3, 140.6, 139.9, 127.8, 127.0, 126.4, 126.2, 126.1, 125.9, 124.8, 119.9, 117.2, 15.7; MS (ESI) *m*/*z* (%, fragment): 249 (100); HRMS (*m*/*z*) for C_13_H_11_ClOS: [M + H]^+^_calcd_ = 250.0219, and [M + H]^+^_measured_ = 250.0216.

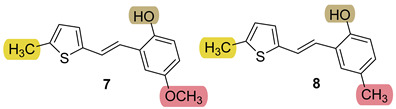



**4-methoxy-2-(2-(5-methylthiophen-2-yl)vinyl)phenol (7):** Repeated column chromatography with a PE/E solvent system (0–60%) yielded 15 mg of pure *trans*-isomer. Mixtures of *cis*- and *trans*-isomers with different ratios were first isolated: 8 mg (*trans*-:*cis*-:aldehyde- = 8:1:1), 20 mg (*trans*-isomer mainly), 12 mg (*trans*-:impurities = 9:1).

**(*E*)-4-methoxy-2-(2-(5-methylthiophen-2-yl)vinyl)phenol (7):** 15 mg (isolated 63%), yellow powder; m.p. 131–133 °C; *Rf* (PE/E = 30%) = 0.63; ^1^H NMR (CDCl_3_, 600 MHz) *δ*/ppm: 7.16 (d, *J* = 16.1 Hz, 1H), 6.98 (d, *J* = 16.1 Hz, 1H), 6.97 (d, *J* = 2.6 Hz, 1H), 6.85 (d, *J* = 3.6 Hz, 1H), 6.72 (d, *J* = 8.6 Hz, 1H), 6.68 (dd, *J* = 8.4, 2.9 Hz, 1H), 6.65–6.64 (m, 1H), 4.72 (s, 1H), 3.79 (s, 3H), 2.48 (s, 3H); ^13^C NMR (CDCl_3_, 150 MHz) *δ*/ppm: 153.9, 147.1, 141.1, 139.5, 126.5, 125.7, 125.2, 123.8, 121.4, 116.8, 114.2, 111.4, 55.8, 15.6; MS (ESI) *m*/*z* (%, fragment): 245 (100), 160 (50); HRMS (*m*/*z*) for C_14_H_14_O_2_S: [M + H]^+^_calcd_ = 246.0715, and [M + H]^+^_measured_ = 246.0708.

**4-methyl-2-(2-(5-methylthiophen-2-yl)vinyl)phenol (8):** Repeated column chromatography with a PE/E solvent system (0–20%) yielded 43 mg of pure *trans*-isomer. Mixtures of *cis*- and *trans*-isomers with different ratios were obtained after the first column chromatography: 39 mg (*trans*-:*cis*-:aldehyde = 6:1:3), 53 mg (*trans*-isomer mainly), 10 mg (impurities).

**(*E*)-4-methyl-2-(2-(5-methylthiophen-2-yl)vinyl)phenol (8):** 43 mg (isolated 47%), yellow powder; m.p. 125–127 °C; *Rf* (PE/E = 30%) = 0.69; ^1^H NMR (CDCl_3_, 600 MHz) *δ*/ppm: 7.25 (d, *J* = 1.7 Hz, 1H), 7.17 (d, *J* = 16.3 Hz, 1H), 6.98 (d, *J* = 16.3 Hz, 1H), 6.91 (dd, *J* = 8.0, 2.2 Hz, 1H), 6.84 (d, *J* = 3.6 Hz, 1H), 6.68 (d, *J* = 8.0 Hz, 1H), 6.64–6.63 (m, 1H), 4.80 (s, 1H), 2.48 (s, 3H), 2.28 (s, 3H); ^13^C NMR (CDCl_3_, 150 MHz) *δ*/ppm: 150.7, 141.3, 139.2, 130.2, 128.9, 127.4, 126.2, 125.7, 124.2, 123.5, 121.5, 115.8, 20.6, 15.6; MS (ESI) *m*/*z* (%, fragment): 229 (100); HRMS (*m*/*z*) for C_14_H_14_OS: [M + H]^+^_calcd_ = 230.0765, and [M + H]^+^_measured_ = 230.0760.

### 3.4. Synthesis of New Thiazolostilbenes ***9**–**14***

Before starting the reaction, the assembled apparatus was purged with nitrogen for 15 min. Then, 50 mL of absolute EtOH were added to the dropping funnel, 30 mL of which were dropped into a three-necked flask. Then, the calculated mass of the thiazole salt **3’** was added to the flask in a ratio of 1:1 in relation to 0.5 g of the corresponding aldehydes in the case of the synthesis of 9–10 and 11 and 1 g of aldehyde in the case of the synthesis of compounds **12**–**14**. The reaction mixture was stirred on a magnetic stirrer at room temperature. After the thiazole salt had dissolved, a calculated mass of sodium was slowly added to the addition funnel with EtOH already present to form a solution of sodium ethoxide. A few drops of the resulting solution were dropped from the funnel into a three-necked flask to make the solution alkaline, and then the aldehyde was added. The remaining sodium ethoxide solution was slowly added dropwise from the funnel into the reaction mixture. The mixture was left to stir for 5 days at room temperature.

The reaction was monitored by TLC plates in the PE/E system. After completion of the reaction, the solvent was evaporated on a rotary evaporator under reduced pressure. The remaining residue was dissolved in 50 mL of toluene and 50 mL of distilled water was added and poured into a separatory funnel. The reaction mixture was extracted with toluene three times, after which the organic layer was dried over anhydrous MgSO_4_. The mixture was filtered into a round flask, and the solvent was evaporated on a rotary evaporator under reduced pressure. Products **9**–**14** were isolated by column chromatography on silica gel with a PE/E solvent system of different ratios (**9**: 0–80%, **10**: 0–100%, **11**–**13**: 0–50% and **14**: 0–20%). For all products, the structure was confirmed by spectroscopic methods, and their spectroscopic characterization is described below.

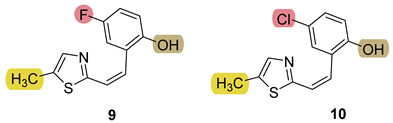



**4-fluoro-2-(2-(5-methylthiazol-2-yl)vinyl)phenol (9):** Repeated column chromatography with a PE/E solvent system (0–80%) yielded 28 mg of the pure *cis*-isomer. Mixtures of *cis*- and *trans*-isomers with different ratios were obtained after the first column chromatography: 28 mg (*cis*-isomer mainly), 94 mg (*trans*-:*cis*- = 0.2:1), 131 mg (*trans*-:*cis*- = 0.3:1), 130 mg (*trans*-:*cis*-:phosphine oxide = 1:1:9).

**(*Z*)-4-fluoro-2-(2-(5-methylthiazol-2-yl)vinyl)phenol (9):** 28 mg (isolated 10%), yellow powder, m.p. 94–95 °C; *Rf* (PE/E = 30%) = 0.35; ^1^H NMR (CDCl_3_, 600 MHz) *δ*/ppm: 7.58 (s, 1H), 7.01–6.97 (m, 1H), 6.96 (d, *J* = 11.5 Hz, 1H), 6.89 (dd, *J* = 8.9, 4.6 Hz, 1H), 6.86 (dd, *J* = 8.6, 3.1 Hz, 1H), 6.41 (d, *J* = 11.5 Hz, 1H), 2.57 (s, 3H); ^13^C NMR (CDCl_3_, 75 MHz) *δ*/ppm: 166.5 (d, *J*_C-F_ = 179.0 Hz), 158.6, 149.0, 144.4, 141.1, 132.9, 125.5, 122.2, 116.7, 116.5 (d, *J*_C-F_ = 23.3 Hz), 115.9 (d, *J*_C-F_ = 23.3 Hz), 19.2; MS (ESI) *m*/*z* (%, fragment): 234 (100); HRMS (*m*/*z*) for C_12_H_10_FNOS: [M + H]^+^_calcd_ = 235.0467, and [M + H]^+^_measured_ = 235.0469.

**4-chloro-2-(2-(5-methylthiazol-2-yl)vinyl)phenol (10):** Repeated column chromatography with a PE/E solvent system (0–100%) yielded 55 mg of pure *cis*-isomer. Following mixtures of *cis*- and *trans*-isomers with different ratios, we first obtained: 85 mg (*trans*-:*cis*- = 1:2), 55 mg (*cis*-isomer mainly), and 415 mg (*trans*-:*cis*-:phosphine oxide = 1:1:20).

**(*Z*)-4-chloro-2-(2-(5-methylthiazol-2-yl)vinyl)phenol (10):** 55 mg (isolated 10%), yellow powder, m.p. 101–103 °C; *Rf* (PE/E = 30%) = 0.36; ^1^H NMR (CDCl_3_, 300 MHz) *δ*/ppm: 7.57 (s, 1H), 7.25 (dd, *J* = 9.1, 2.9 Hz, 1H), 7.13 (d, *J* = 2.4 Hz, 1H), 6.96 (d, *J* = 11.7 Hz, 1H), 6.87 (d, *J* = 8.7 Hz, 1H), 6.40 (d, *J* = 11.7 Hz, 1H), 5.56 (s, 1H), 2.57 (s, 3H); ^13^C NMR (CDCl_3_, 75 MHz) *δ*/ppm: 167.8, 151.7, 143.3, 132.9, 129.8, 129.3, 125.6, 125.5, 124.5, 122.1, 117.3, 19.2; MS (ESI) *m*/*z* (%, fragment): 250 (100); HRMS (*m*/*z*) for C_12_H_10_ClNOS: [M + H]^+^_calcd_ = 251.0172, and [M + H]^+^_measured_ = 251.0165. 

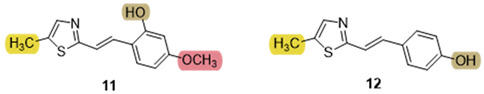



**5-methoxy-2-(2-(5-methylthiazol-2-yl)vinyl)phenol (11):** Repeated column chromatography with a PE/E solvent system (0–50%) yielded 30 mg of pure *trans*-isomer. Mixtures of *cis*- and *trans*-isomers with different ratios were first obtained: 24 mg (*trans*-:*cis*- = 5:1), 31 mg (*trans*-:*cis*- = 1.5:1), 12 mg (*trans*-:*cis*- = 1:1). A fraction of 24 mg (*trans*-:*cis*- = 5:1) was again placed on the column with the PE/E system (70%), after which 19.5 mg of the *trans*-isomer was obtained with few impurities. Fractions of 31 mg (*trans*-:*cis*- = 1.5:1) and 12 mg (*trans*-:*cis*- = 1:1) were put together on a column with a PE/E system (70%) and 30 mg of the *trans*-isomer with few impurities were obtained.

**(*E*)-5-methoxy-2-(2-(5-methylthiazol-2-yl)vinyl)phenol (11):** 30 mg (isolated 2.9%) of white powder, m.p. 119–121 °C; *Rf* (PE/E = 60%) = 0.73; UV (ACN) *λ*_max_/nm (*ε*/dm^3^mol^−1^cm^−1^): 338 (26408); ^1^H NMR (CDCl_3_, 600 MHz) *δ*/ppm: 7.47 (s, 1H), 7.34 (d, *J* = 8.6 Hz, 1H), 7.13 (d, *J* = 16.4 Hz, 1H), 7.01 (d, *J* = 16.4 Hz, 1H), 6.46 (dd, *J* = 8.6, 2.5 Hz, 1H), 6.37 (d, *J* = 2.4 Hz, 1H), 3.78 (s, 3H), 2.68 (s, 3H); MS (ESI) *m*/*z* (%, fragment): 248 (100); HRMS (*m*/*z*) for C_13_H_13_NO_2_S: [M + H]^+^_calcd_ = 247.0667, and [M + H]^+^_measured_ = 247.0667.

**4-(2-(5-methylthiazol-2-yl)vinyl)phenol (12):** Column chromatography with the PE/E system (0–50%) yielded 48 mg of pure *trans*-isomer. In this case, the pure *cis*-isomer was not obtained.

**(*E*)-4-(2-(5-methylthiazol-2-yl)vinyl)phenol (12):** 48 mg (isolated 4.8%), white powder; m.p. 110–112 °C; *Rf* (PE/E = 50%) = 0.29; UV (ACN) *λ*_max_/nm (*ε*/dm^3^mol^−1^cm^−1^): 332 (23494); ^1^H NMR (CDCl_3_, 600 MHz) *δ*/ppm: 7.51 (s, 1H), 7.34 (d, *J* = 8.9 Hz, 2H), 7.01 (d, *J* = 16.2 Hz, 1H), 6.82 (d, *J* = 8.9 Hz, 2H), 6.73 (d, *J* = 16.2 Hz, 1H), 5.10 (s, 1H), 2.70 (s, 3H); ^13^C NMR (CDCl_3_, 150 MHz) *δ*/ppm: 164.3, 155.6, 140.1, 130.5, 129.5, 128.6, 127.8, 116.5, 115.7, 29.8; MS (ESI) *m*/*z* (%, fragment): 218 (100); HRMS (*m*/*z*) for C_12_H_11_NOS: [M + H]^+^_calcd_ = 217.0561, and [M + H]^+^_measured_ = 217.0561. 

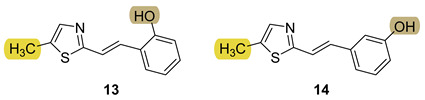



**2-(2-(5-methylthiazol-2-yl)vinyl)phenol (13):** After repeated column chromatography with the PE/E system (0–50%), 80 mg of the majority *trans*-isomer and 10 mg of a mixture of *cis*- and *trans*-isomers were obtained. For the fraction of 80 mg, column chromatography was performed again with the PE/E system (70%), which resulted in 64 mg of a pure *trans*-isomer and 11 mg of a mixture of *trans*- and *cis*-isomers. 

**(*E*)-2-(2-(5-methylthiazol-2-yl)vinyl)phenol (13):** 64 mg (isolated 7.4%), white powder; m.p. 133–134 °C; *Rf* (PE/E = 50%) = 0.18; UV (ACN) *λ*_max_/nm (*ε*/dm^3^mol^−1^cm^−1^): 327 (22333), 309 (20984), 298 (20114); ^1^H NMR (CDCl_3_, 600 MHz) *δ*/ppm: 7.51 (s, 1H), 7.43 (dd, *J* = 7.8, 1.3 Hz, 1H), 7.38 (d, *J* = 16.7 Hz, 1H), 7.25 (d, *J* = 16.7 Hz, 1H), 7.11 (t, *J* = 6.7 Hz, 1H), 6.87 (t, *J* = 7.6 Hz, 1H), 6.81 (dd, *J* = 8.1, 1.1 Hz, 1H), 2.69 (s, 3H); MS (ESI) *m*/*z* (%, fragment): 218 (100); HRMS (*m*/*z*) for C_12_H_11_NOS: [M + H]^+^_calcd_ = 217.0561, and [M + H]^+^_measured_ = 217.0561.

**3-(2-(5-methylthiazol-2-yl)vinyl)phenol (14):** After column chromatography with the PE/E system (0–20%), 21 mg of mainly *cis*-isomer was obtained, and mixtures of two isomers in different ratios were obtained as follows: 164 mg (*trans*-:*cis*- = 1:6), 336 mg (*trans*-:*cis*- = 1:1.3), 93 mg (*trans*-:*cis*- = 1:2.5) with a minor amount of phosphine oxide and 161 mg (*trans*-:*cis*- = 5:1) also with a minor amount phosphine oxide. For the fraction of 161 mg, preparative thin-layer chromatography was performed in the PE/E system (60%). The sample was dissolved in DCM and applied to a TLC plate. Then, 2.5 mg of the pure trans-isomer were obtained from the described procedure.

**(*E*)-3-(2-(5-methylthiazol-2-yl)vinyl)phenol (14):** 2.5 mg (isolated 2.5%), white powder; m.p. 127–128 °C; *Rf* (PE/E = 50%) = 0.50; UV (ACN) *λ*_max_/nm (*ε*/dm^3^mol^−1^cm^−1^): 316 (20947); ^1^H NMR (CDCl_3_, 600 MHz) *δ*/ppm: 7.53 (s, 1H), 7.19 (t, *J* = 7.7 Hz, 1H), 7.10 (d, *J* = 16.2 Hz, 1H), 6.96 (d, *J* = 7.9 Hz, 1H), 6.92 (t, *J* = 2.2 Hz, 1H), 6.76 (dd, *J* = 8.2, 2.5 Hz, 1H), 6.73 (d, *J* = 16.2 Hz, 1H), 2.68 (s, 3H); ^13^C NMR (CDCl_3_, 150 MHz) *δ*/ppm: 165.3, 157.1, 140.2, 138.0, 137.9, 131.4, 129.9, 118.4, 118.3, 115.4, 112.9, 103.4, 19.2; MS (ESI) *m*/*z* (%, fragment): 218 (100); HRMS (*m*/*z*) for C_12_H_11_NOS: [M + H]^+^_calcd_ = 217.0561, and [M + H]^+^_measured_ = 217.0561.

### 3.5. Cholinesterase Inhibition and Antioxidative Potential

#### 3.5.1. In Vitro ChE Activity Assay

The inhibitory effect of the new heteroaromatic resveratrol analogs **1**–**14** on acetylcholinesterase (eeAChE) and butyrylcholinesterase (eqBChE) activity was tested by the modified Ellman’s method [52]. Tris-HCl buffer, eeAChE (from electric eel, type VI-S), eqBChE (from equine serum), acetylthiocholine iodide (ATChI), S-butyrylthiocholine iodide (BTChI), galantamine hydrobromide, 96% ethanol, and Ellman’s reagent (DTNB, 5,5′-dithiobis-(2-nitrobenzoic acid)) were purchased from Sigma–Aldrich (St. Louis, MO, USA). Ethanol was used to dissolve heteroaromatic resveratrol analogs. Enzymes were prepared in 20 mM Tris buffer pH 7.5 and DTNB, ATChI, and BTChI in 50 mM Tris buffer pH 8.0. Cholinesterase activity was evaluated using a 96-well microplate reader (Bio Tek 800TSUV Absorbance Reader, Agilent, Santa Clara, CA, USA) at room temperature. The microplate well was filled with 180 μL of Tris buffer (50 mM, pH 8.0), 10 μL of tested solutions (final concentrations in a range of 20–1000 μM), 10 μL of an enzyme (final concentration 0.04 U/mL), 10 μL of DTNB (final concentration 0.3 mM), and 10 mL of ATChI/BTChI (final concentration of 0.5 mM). The absorbance was measured at 405 nm after 5 min. Without the use of inhibitors and enzymes, non-enzymatic hydrolysis was evaluated as a blank for the control measurement. The non-enzymatic hydrolysis reaction with an added inhibitor was used as a blank for the samples. The same volume of buffer was substituted for the enzyme. All experiments were run in triplicate. The following formula was applied to calculate the inhibition percentage: Inhibition (%) = [(A_C_ − A_T_)/A_C_] × 100, where A_C_ is the activity of the enzyme without a test sample and A_T_ is the activity of the enzyme with a test sample. The results are represented as mean values ± standard deviation. The inhibitory activity of ethanol was subtracted from all the samples. The IC_50_ values were obtained by a nonlinear fit of compound concentration values versus the response. All investigated compounds were tested against both enzymes in a wide range of concentrations, and if more than 50% inhibition was achieved, IC_50_ values were calculated.

#### 3.5.2. Antioxidative Potential

In this research, the antioxidant activity of heteroaromatic resveratrol analogs was evaluated using two methods: the DPPH radical scavenging assay and the CUPRAC reducing-antioxidant-capacity test.

**DPPH radical scavenging activity.** The stable radical 1,1-diphenyl-2-picrylhydrazyl (DPPH, Sigma–Aldrich, St. Louis, MO, USA) is the reagent used in this spectrophotometric test. The DPPH ethanolic solution (*c* = 8⋅10^−4^ mol/L) was prepared daily and stored in a dark flask at 4 °C between the measurements. The antioxidant potential of new heterocyclic resveratrol analogues was evaluated by the Brand–Williams method [60]. Briefly, 50 μL of test solutions of different concentrations (final concentrations: 5–500 μM) were added to 1 mL of DPPH solution. The reaction mixture was vortexed and incubated in the dark for 30 min at 25 °C. Absorbance was measured at 517 nm (UV-1800 UV/Vis Spectrophotometer, Shimadzu, Kyoto, Japan). Antioxidant substances reduce the DPPH radical and decrease its absorbance at this wavelength. The DPPH inhibition, reported as a percentage, was obtained according to the equation: Inhibition (%) = [(A_C(0)_ − A_A(t)_)/A_C(0)_] × 100, where A_C(0)_ is the absorbance of the control at *t* = 0 min and A_A(t)_ is the absorbance of the antioxidant at *t* = 30 min. Each of the measurements was conducted three times. Inhibition percentages were expressed as mean values ± standard deviation. IC_50_ values were calculated using a nonlinear fit of compound concentration values versus the inhibition percentage. The values were calculated for the components that achieved more than 50% quenching of DPPH radicals.

**CUPRAC-reducing-antioxidant-capacity assay.** The CUPRAC-reducing-antioxidant-capacity test of heteroaromatic resveratrol analogs was determined according to the Apak et al. method [61]. Ammonium acetate, neocuproine, copper(II) chloride, and standard Trolox were purchased from Sigma–Aldrich (St. Louis, MO, USA). The reaction mixture contained 1 mL of each of these solutions: NH_4_Ac buffer (1 M, pH 7.0), Cu(II) chloride (10 mM), and neocuproine (7.5 mM, dissolved in ethanol). To make a final volume of 4.1 mL, x mL of the testing sample (or standard Trolox) and (1,1-x) mL of H_2_O were added to an initial mixture. The tubes were vortexed and left for 30 min at room temperature. As a blank test, the same mixture was used only without the test sample. Absorbance was recorded at 450 nm (UV-1800 UV/Vis spectrophotometer, Shimadzu, Kyoto, Japan). A linear calibration graph for Trolox in the concentration range of 7–67 μM was prepared. The corresponding regression calibration equation was: A = 14.225*x* + 0.0007, where A is the absorbance at 450 nm and *x* is the concentration of Trolox (R² = 0.9904). The CUPRAC results were presented as a mole of Trolox equivalent (TE) per mole of the tested compound.

### 3.6. Computational Details

The conformational investigation and geometry optimizations of the selected ligands were conducted using the Gaussian16 program package [62] at the M06-2X/6-31G(d) level of theory. The optimized structures of the most stable conformers were used as flexible ligands in molecular docking. The molecular docking studies were performed using the Autodock program package (AutoDock4) [63], with the crystal structures 4EY7.pdb [64] and 1P0I.pdb [65] for AChE and BChE, respectively, taken from the Protein Data Bank. Docking simulations were performed with the Lamarckian Genetic Algorithm, which generated 25 genetic algorithm dockings with 25 binding poses for each ligand, with the rigid residues of the enzymes during the docking.

## 4. Conclusions

In this research, resveratrol analogs **1**–**14** were synthesized via the Wittig reaction using heterocyclic triphenylphosphonium salts and various benzaldehydes. *Trans*-resveratrol is well-known for its potential as a neuroprotective agent against neurodegenerative diseases, along with its notable antioxidative properties. Here, the aim was to synthesize compounds with the trans-configuration, mirroring the biologically active form of *trans*-resveratrol. Notably, Wittig’s reactions with unsubstituted triphenylphosphonium salt produced the highest yields of the new resveratrol analogs **1**–**14**. Enzyme BChE exhibited greater sensitivity to the heteroaromatic resveratrol analogs than AChE, except for compound **6**, the methylated thiophene derivative with chlorine, which inhibited both enzymes equally. Compounds **5** and **8** achieved the highest BChE inhibition with IC_50_ values of 22.9 and 24.8 μM, respectively. Resveratrol analogs containing methylated thiophene subunits exhibited better inhibition of both AChE and BChE compared to their unmethylated counterparts. According to DPPH and CUPRAC antioxidant spectrophotometric methods, the heteroaromatic resveratrol analogs with ortho-OH and electron-donating methoxy and methyl groups on the meta position of phenol ring (molecules **3**, **4**, **7**, **8**, **11**) exhibited more potent antioxidant activity than the standard resveratrol. Compounds **7** and **8** notably possess significant antioxidant activity and cholinesterase inhibitory properties. Molecular docking of selected compounds into cholinesterases was performed to illustrate the ligand-active site complexes’ structure and identify the non-covalent interactions responsible for the stability of these complexes. The in silico ADME analysis indicated that compounds **5** and **7** are the most promising candidates for early-stage drug development. Regarding genotoxic safety, compound **8** appears to be the most promising lead.

## Figures and Tables

**Figure 3:**
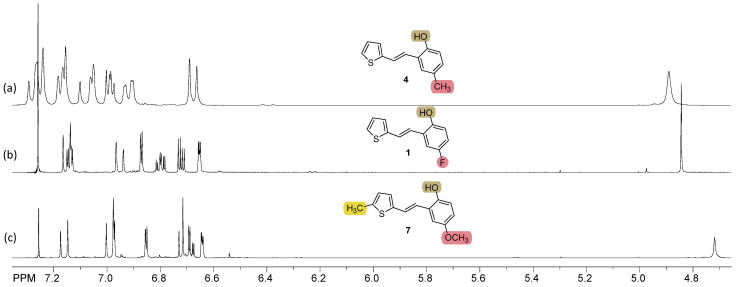
Compared parts of the ^1^H NMR spectra of some representatives of the new resveratrol analogs: (**a**) **4**, (**b**) **1**, and (**c**) **7**.

**Table 1:** Cholinesterase inhibition (eeAChE, eqBChE) and antioxidative potential (DPPH, CUPRAC) of derivatives **1**–**14**.

Compound	eeAChE		eqBChE		DPPH		CUPRAC
	IC_50_ (μM)	Inhibition ** (%)	IC_50_ (μM)	Inhibition ** (%)	IC_50_ (μM)	Inhibition ** (%)	mol TE/mol of Compound
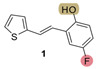	177.7	80.9 ± 1.9 (500)	66.0	83.5 ± 0.8 (400)	159.2	77.2 ± 0.5 (500)	0.925
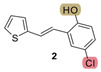	82.3	83.0 ± 2.5 (250)	53.0	80.4 ± 6.4 (200)	265.1	65.0 ± 2.4 (500)	0.713
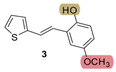	404.6	77.8 ± 5.2 (500)	206.4	68.5 ± 6.2 (500)	36.2	87.7 ± 0.8 (500)	1.111
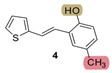	225.0	82.1 ± 1.1 (250)	36.5	81.1 ± 2.2 (250)	51.4	90.3 ± 0.6 (400)	0.852
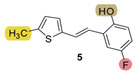	154.9	84.6 ± 2.7 (250)	22.9	75.3 ± 2.3 (100)	130.3	83.0 ± 1.9 (400)	0.204
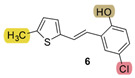	27.1	82.4 ± 0.8 (80)	39.7	84.0 ± 1.8 (100)	- *	42.9 ± 2.7 (400)	1.407
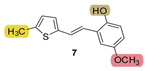	62.9	81.1 ± 1.1 (200)	42.5	83.1 ± 0.8 (200)	23.8	86.2 ± 2.3 (50)	2.350
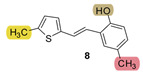	92.4	78.2 ± 7.7 (150)	24.8	88.5 ± 5.0 (250)	26.3	89.3 ± 1.2 (250)	1.148
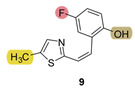	- *	17.6 ± 3.1 (100)	- *	10.5 ± 4.1 (250)	428.4	55.4 ± 0.7 (500)	1.000
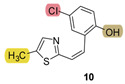	- *	34.2 ± 2.1 (500)	- *	22.4 ± 9.0 (500)	- *	24.0 ± 0.7 (500)	0.630
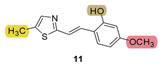	- *	29.3 ± 1.8 (500)	- *	9.8 ± 2.7 (500)	30.1	89.8 ± 0.0 (524)	n.d. ***
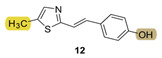	- *	9.1 ± 1.4 (500)	189.5	68.4 ± 1.3 (500)	139.8	74.1 ± 0.4 (524)	n.d. ***
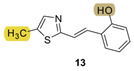	- *	17.1 ± 3.9 (500)	30.7	78.2 ± 3.8 (500)	699.2	59.8 ± 2.3 (1047)	n.d. ***
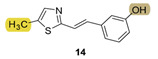	- *	10.7 ± 2.4 (250)	- *	15.6 ± 3.6 (250)	- *	11.2 ± 2.7 (250)	n.d. ***
Resveratrol	n.d. ***		n.d. ***		74.0 [48]		n.d. ***
Galantamine	0.15 [48]		7.9 [46]		n.d. ***		n.d. ***

* Not achieved an IC_50_ value. ** The numbers shown in parentheses represent maximal concentrations tested in μM. *** Not determined.

**Table 2:** In silico ADMET properties of compounds **5**, **6**, **7,** and **8** with potential as biologically active substances.

		Compound 5	Compound 6	Compound 7	Compound 8	
Property	Property Measure	Predicted Value				Unit
Absorption	Water solubility	−4.558	−4.938	−4.511	−4.592	log mol/L
	Caco2 permeability	1.338	1.497	1.348	1.489	log Papp in 10^−6^ cm/s
	Intestinal absorption (human)	91.111	90.018	92.389	91.477	% Absorbed
	Skin Permeability	−2.046	−1.9	−2.109	−1.87	log Kp
	P-glycoprotein substrate	Yes	No	No	No	
	P-glycoprotein I inhibitor	No	No	No	No	
	P-glycoprotein II inhibitor	No	No	No	No	
Distribution	VDss (human)	0.387	0.686	0.453	0.726	log L/kg
	Fraction unbound (human)	0.035	0.013	0.024	0.026	Fu
	BBB permeability	0.345	0.294	0.315	0.344	log BB
	CNS permeability	−1.626	−1.565	−1.69	−1.565	log PS
Metabolism	CYP2D6 substrate	No	No	No	No	
	CYP3A4 substrate	Yes	Yes	Yes	Yes	
	CYP1A2 inhibitior	Yes	Yes	Yes	Yes	
	CYP2C19 inhibitior	Yes	Yes	Yes	Yes	
	CYP2C9 inhibitior	No	Yes	No	Yes	
	CYP2D6 inhibitior	No	No	No	No	
	CYP3A4 inhibitior	No	No	No	No	
Excretion	Total Clearance	−0.146	−0.126	0.035	−0.109	log mL/min/kg
	Renal OCT2 substrate	No	No	No	No	
Toxicity	AMES toxicity	No	No	No	No	
	Max. tolerated dose (human)	0.574	0.639	0.613	0.647	log mg/kg/day
	hERG I inhibitor	No	No	No	No	
	hERG II inhibitor	No	No	No	No	
	Oral Rat Acute Toxicity (LD50)	2.376	2.421	2.407	2.235	mol/kg
	Oral Rat Chronic Toxicity (LOAEL)	2.167	1.116	2.204	1.165	log mg/kg_bw/day
	Hepatotoxicity	No	Yes	No	Yes	
	Skin Sensitisation	No	Yes	No	Yes	
	T.Pyriformis toxicity	1.738	2.383	1.868	2.143	log ug/L
	Minnow toxicity	0.334	0.126	0.177	0.344	

**Table 3:** Lhasa M7 evaluation of mutagenic potential of compounds **1**–**14**.

Structure	ICH M7 Class	Derek Prediction	Sarah Prediction	Overall In Silico
**1**	Class 5	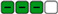	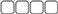	Negative
**2**	Class 3	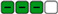	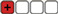	Positive
**3**	Class 5	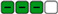	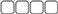	Negative
**4**	Class 5	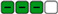	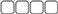	Negative
**5**	Class 5	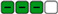	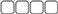	Negative
**6**	Class 3	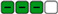	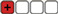	Positive
**7**	Class 5	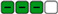	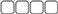	Negative
**8**	Class 5	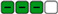	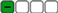	Negative
**9**	Class 5	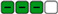	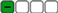	Negative
**10**	Class 3	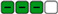	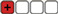	Positive
**11**	Class 5	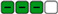	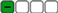	Negative
**12**	Class 5	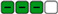	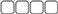	Negative
**13**	Class 5	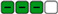	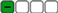	Negative
**14**	Class 5	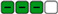	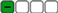	Negative

## Data Availability

The data presented in this study are available on request from the corresponding author. The data are not publicly available due to privacy.

## Supplementary Materials

The following supporting information can be downloaded at: https://www.mdpi.com/article/10.3390/ijms25137401/s1.
