# Supplementary material for: Cholinesterase Inhibition and Antioxidative Capacity of New Heteroaromatic Resveratrol Analogs: Synthesis and Physico—Chemical Properties
Source: Int J Mol Sci. 2024 Jul 5;25(13):7401. doi: 10.3390/ijms25137401 (PMC11242640; doi:10.3390/ijms25137401)
Supplement: Supplementary file 1 [file ijms-25-07401-s001.zip › ijms-3093488-supplementary.pdf]

# Cholinesterase inhibition and antioxidative capacity of new heteroaromatic resveratrol analogs: synthesis and physico-chemical properties

**Milena Mlakić [1], Ilijana Odak [2], Danijela Barić [3], Ivana Šagud [4], Stanislava Talić [2,*] and Irena Škorić [1,*]**

[1] Department of Organic Chemistry, Faculty of Chemical Engineering and Technology, University of Zagreb, Marulićev trg 19, HR-10 000 Zagreb, Croatia; mdragojev@fkit.unizg.hr (M.M.)

[2] Department of Chemistry, Faculty of Science and Education, University of Mostar, Matice hrvatske bb, 88 000 Mostar, Bosnia and Herzegovina; ilijana.odak@fpmoz.sum.ba (I.O.)

[3] Group for Computational Life Sciences, Division of Physical Chemistry, Ruđer Bošković Institute, Bijenička cesta 54, HR-10 000 Zagreb, Croatia; dbaric@irb.hr (D.B.)

[4] Croatian Agency for Medicinal Products and Medical Devices, Ksaverska Cesta 4, HR-10 000 Zagreb, Croatia; Ivana.Sagud@halmed.hr (I.S.)

[*] Correspondence: Prof Stanislava Talić; stanislava.talic@fpmoz.sum.ba (S.T.); Prof Irena Škorić; iskoric@fkit.unizg.hr (I.Š.)

# Supplementary Data

## Contents

1. NMR spectra of compounds **1–14**……………………………………………………..……..……..2
2. Mass spektra and HRMS analyses of compounds **1–14**………………………..……...………..59
3. UV spectra of compounds **11–14**…………………………………………………………………….73
4. Cartesian coordinates of docked ligands…………………………………………………………75
5. Tables S1 and S2, free energies of binding obtained by docking…………………………………77
6. Docked molecule **6**………………………………………………………………………………….78

## **1.** NMR spectra of compounds **1–14**

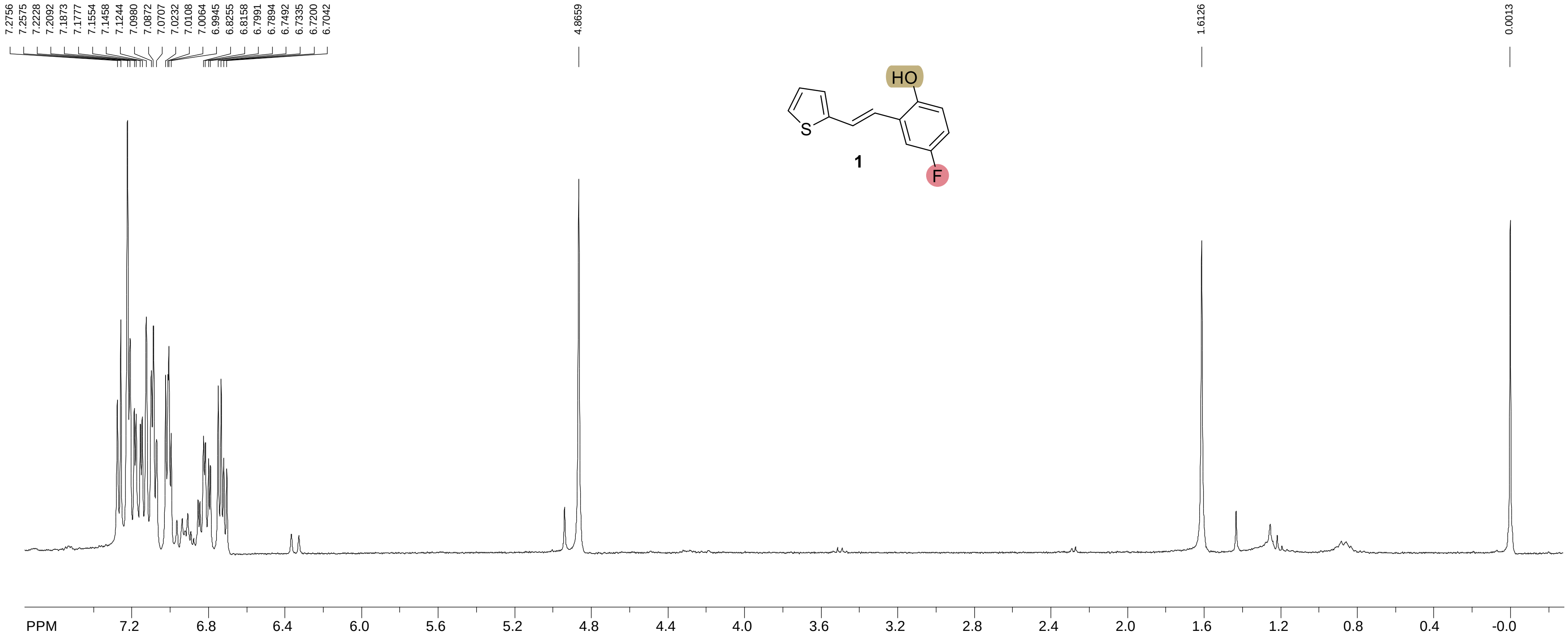


**Figure S1.** $^1$H NMR ($CDCl_3$) spectrum of **1**.

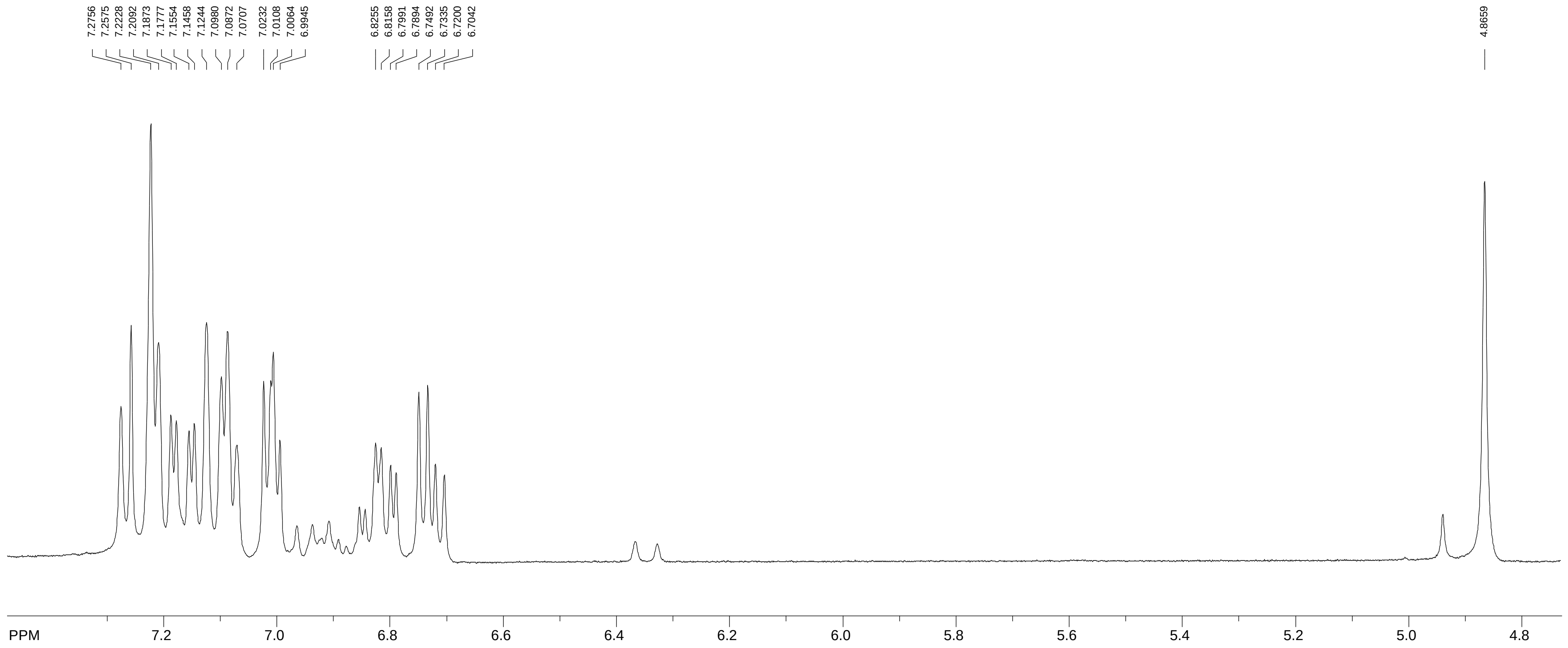


**Figure S2. P**art of $^{1}$H NMR ($CDCl_3$) spectrum of **1**.

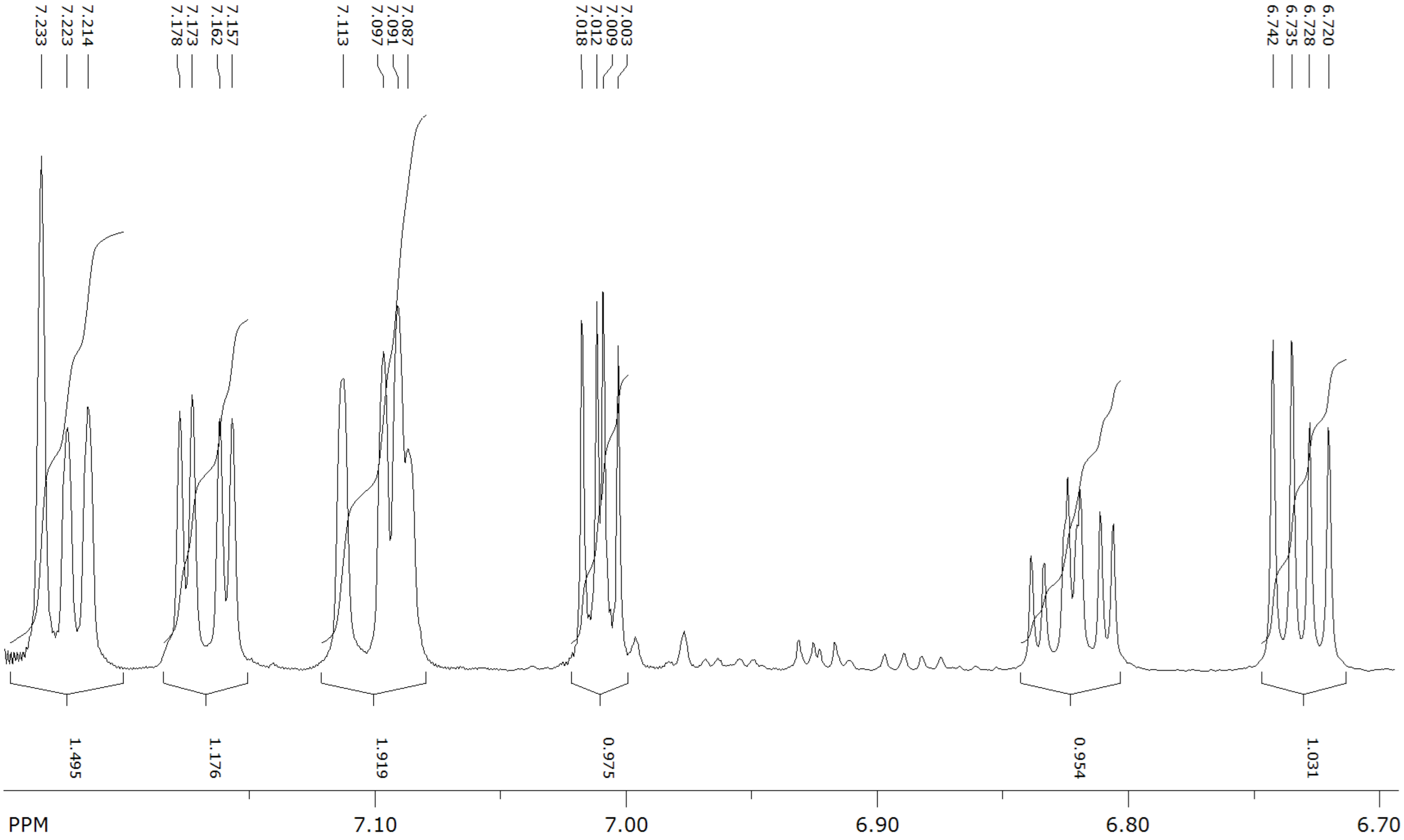


**Figure S3. A**romatic part of $^{1}$H NMR ($CDCl_3$) spectrum of **1**.

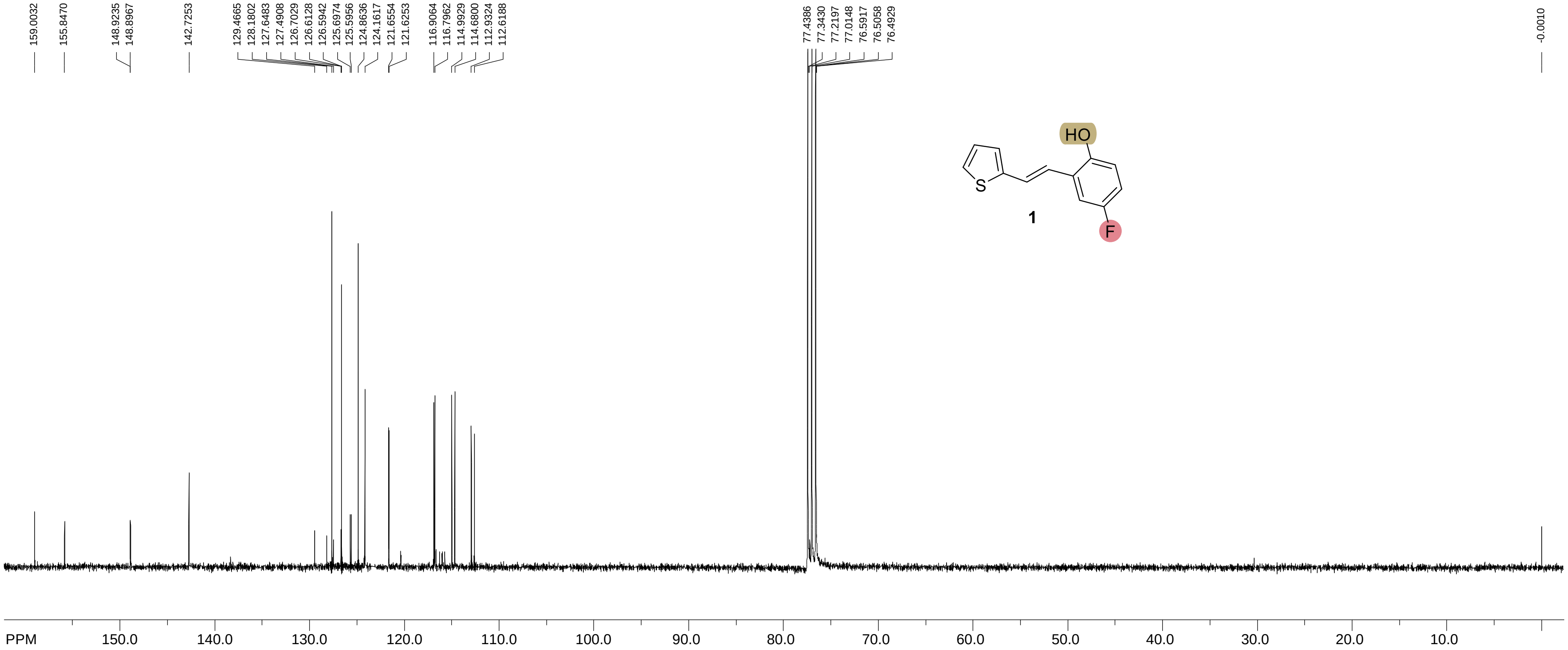


**Figure S4.** $^{13}C$ NMR ($CDCl_3$) spectrum of **1**.

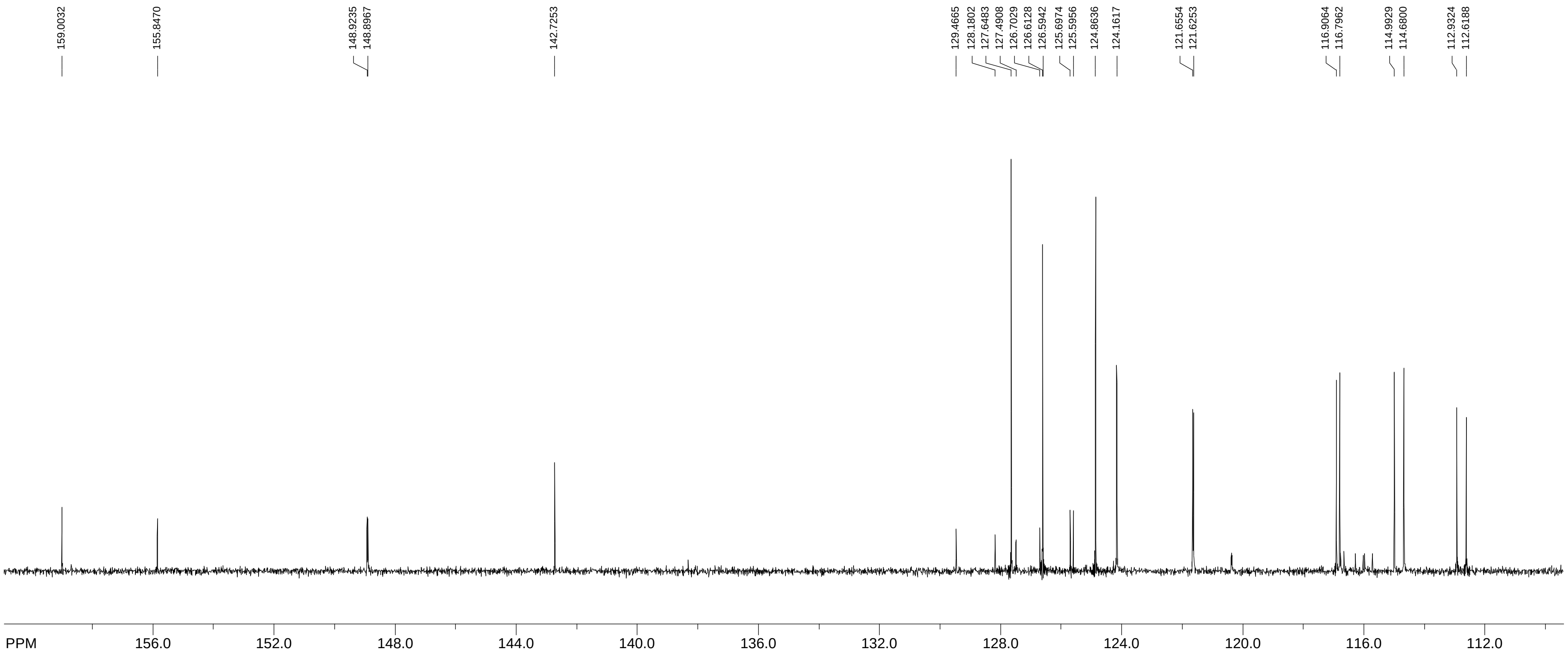


**Figure S5.** Aromatic part of $^{13}C$ NMR ($CDCl_3$) spectrum of **1**.

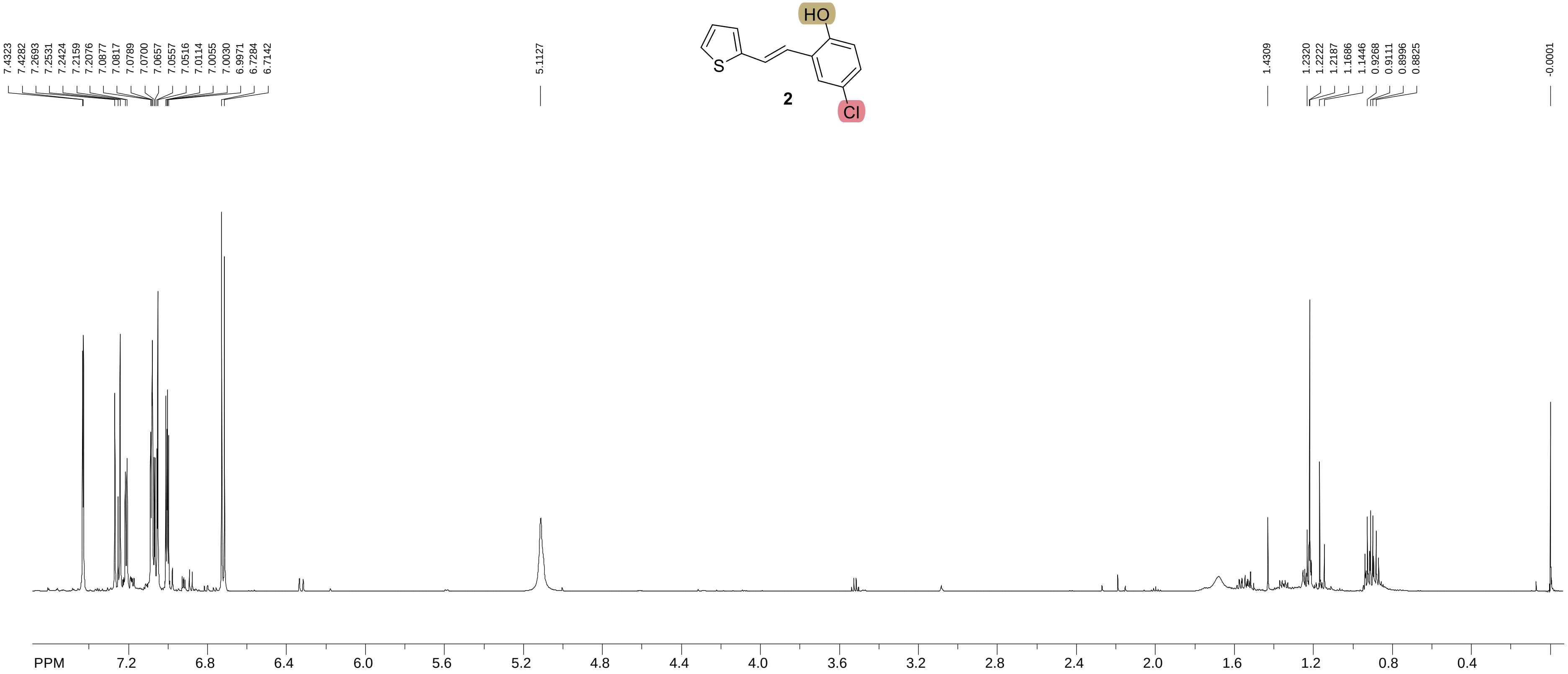


**Figure S6.** $^{1}H$ NMR ($CDCl_3$) spectrum of **2**.

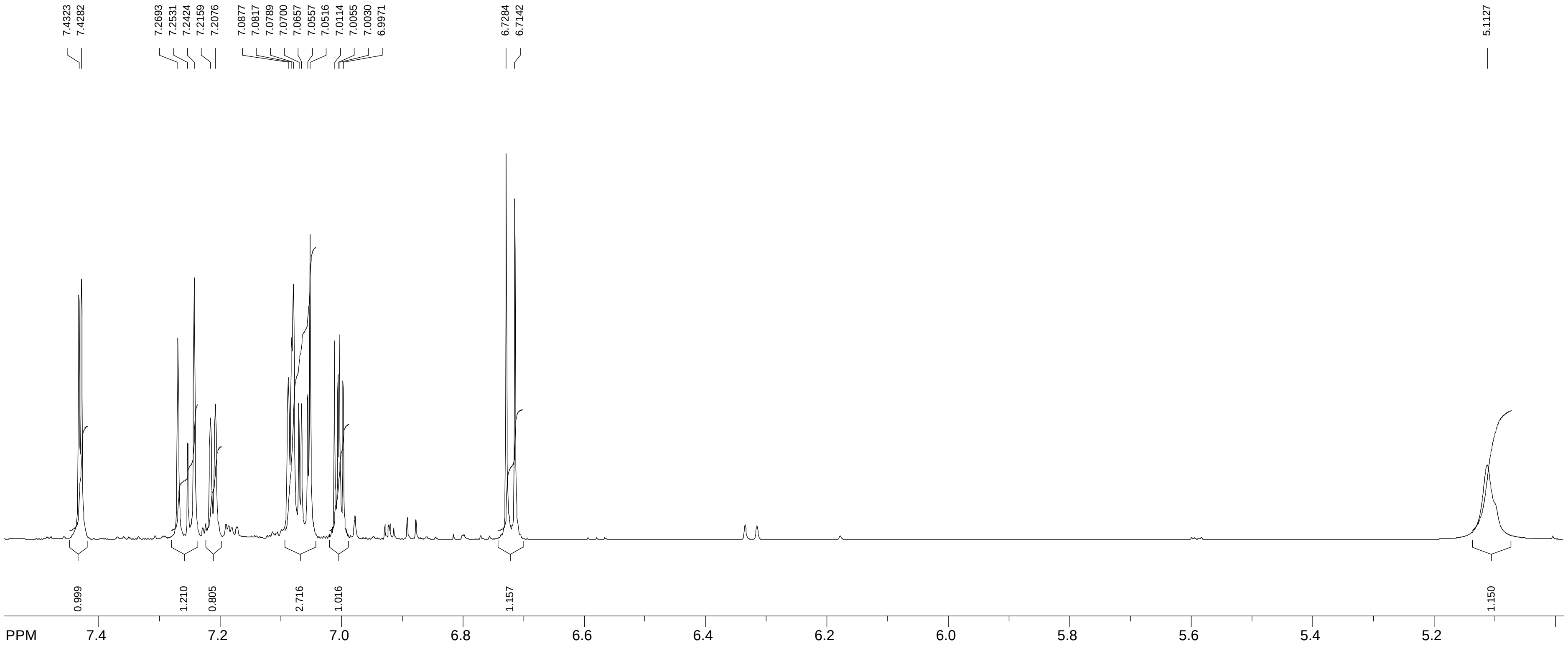


**Figure S7. P**art of $^{1}$H NMR ($CDCl_3$) spectrum of **2**.

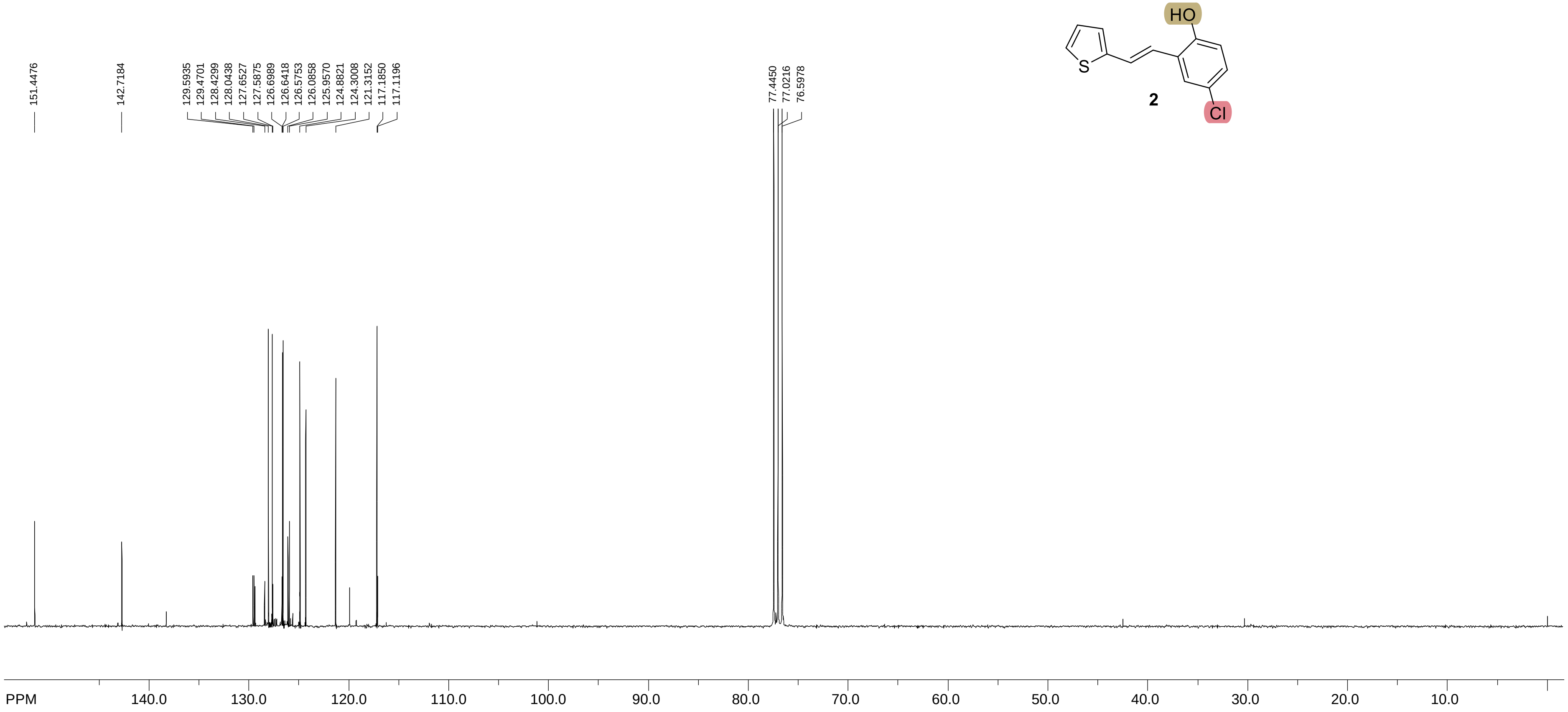


**Figure S8.** $^{13}C$ NMR ($CDCl_3$) spectrum of **2**.

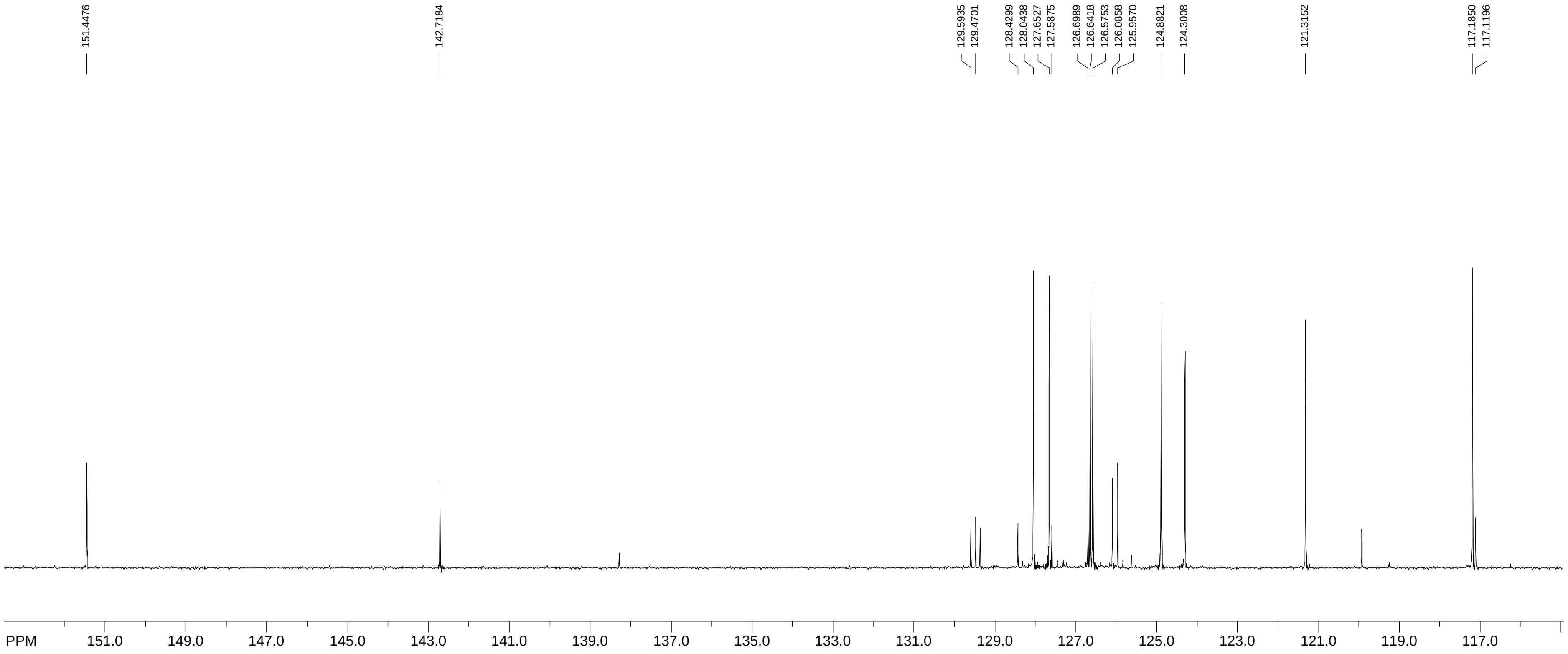


**Figure S9. A**romatic part of $^{13}C$ NMR ($CDCl_3$) spectrum of **2**.

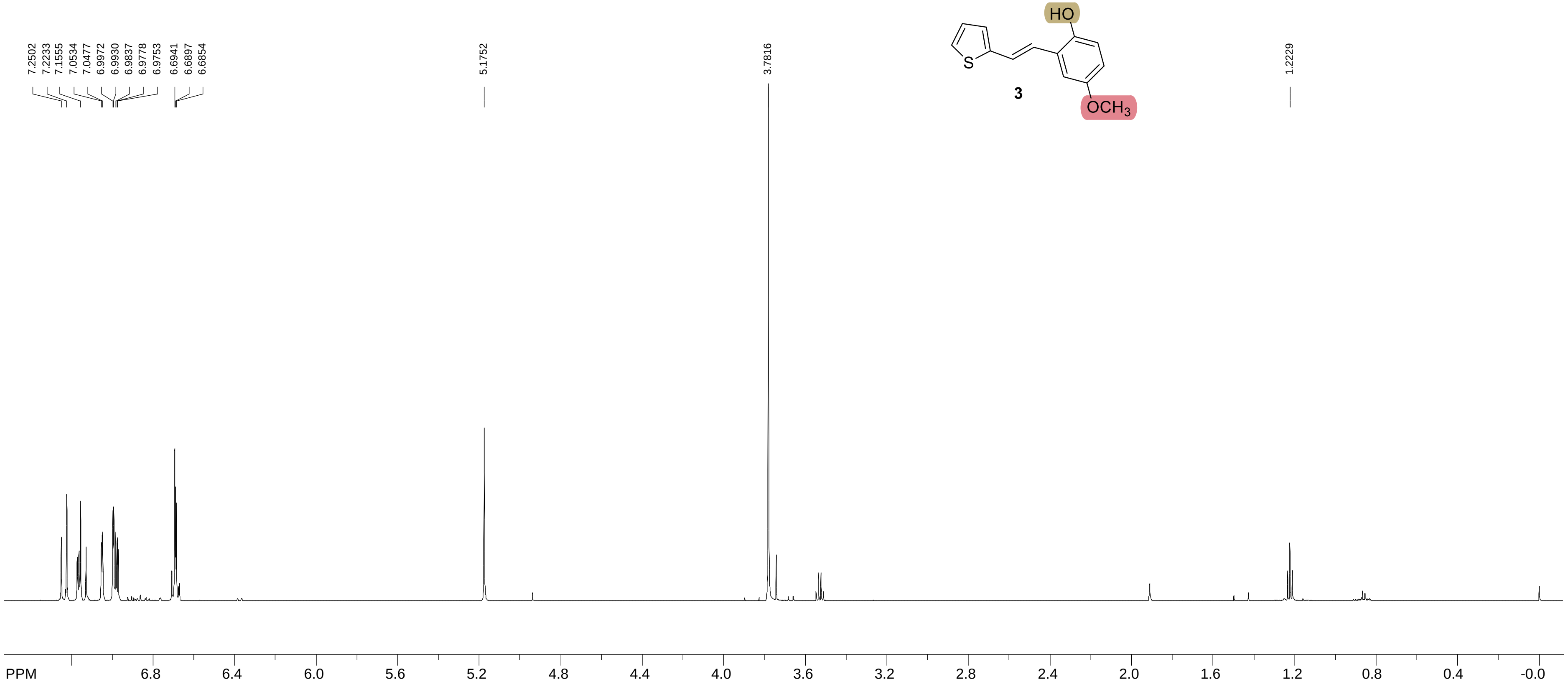


**Figure S10.** $^{1}H$ NMR ($CDCl_3$) spectrum of **3**.

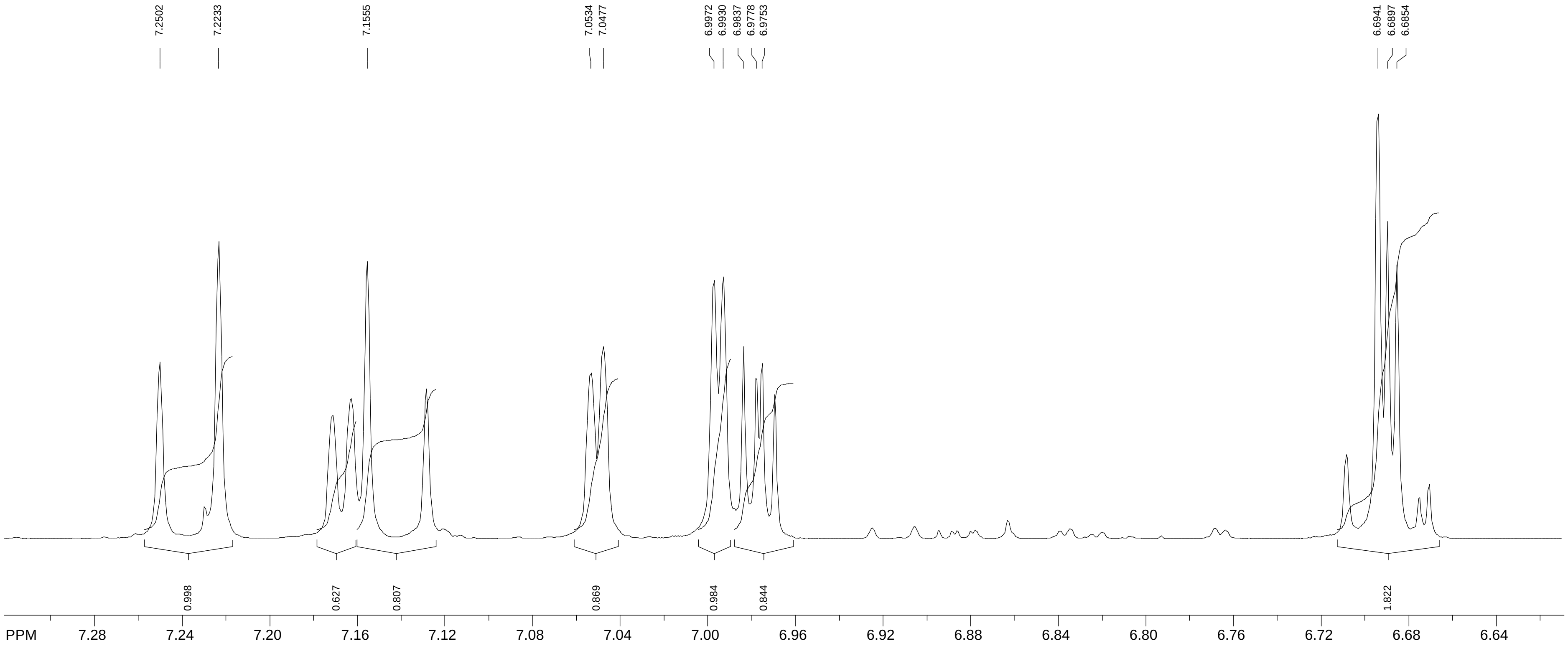


**Figure S11. A**romatic part of $^{1}$H NMR ($CDCl_3$) spectrum of **3**.

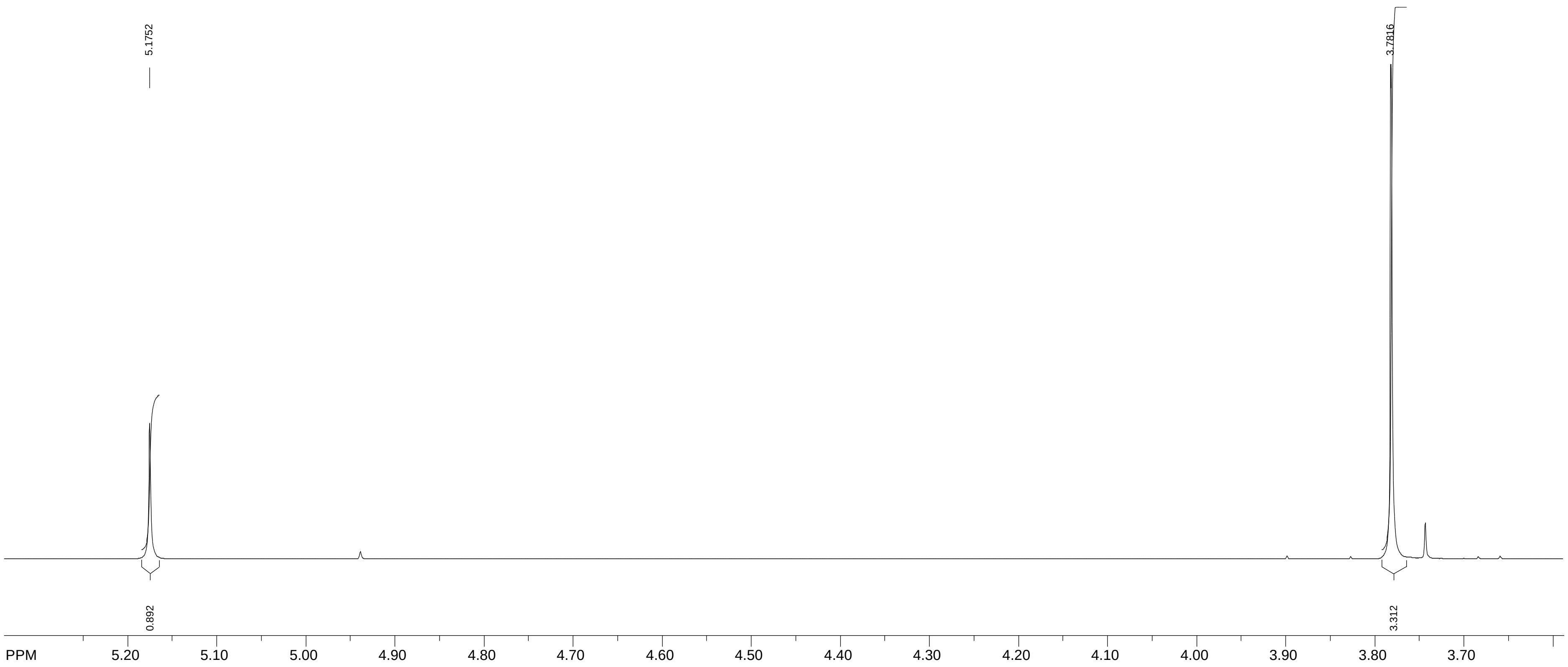


**Figure S12. A**liphatic part of $^{1}H$ NMR ($CDCl_3$) spectrum of **3**.

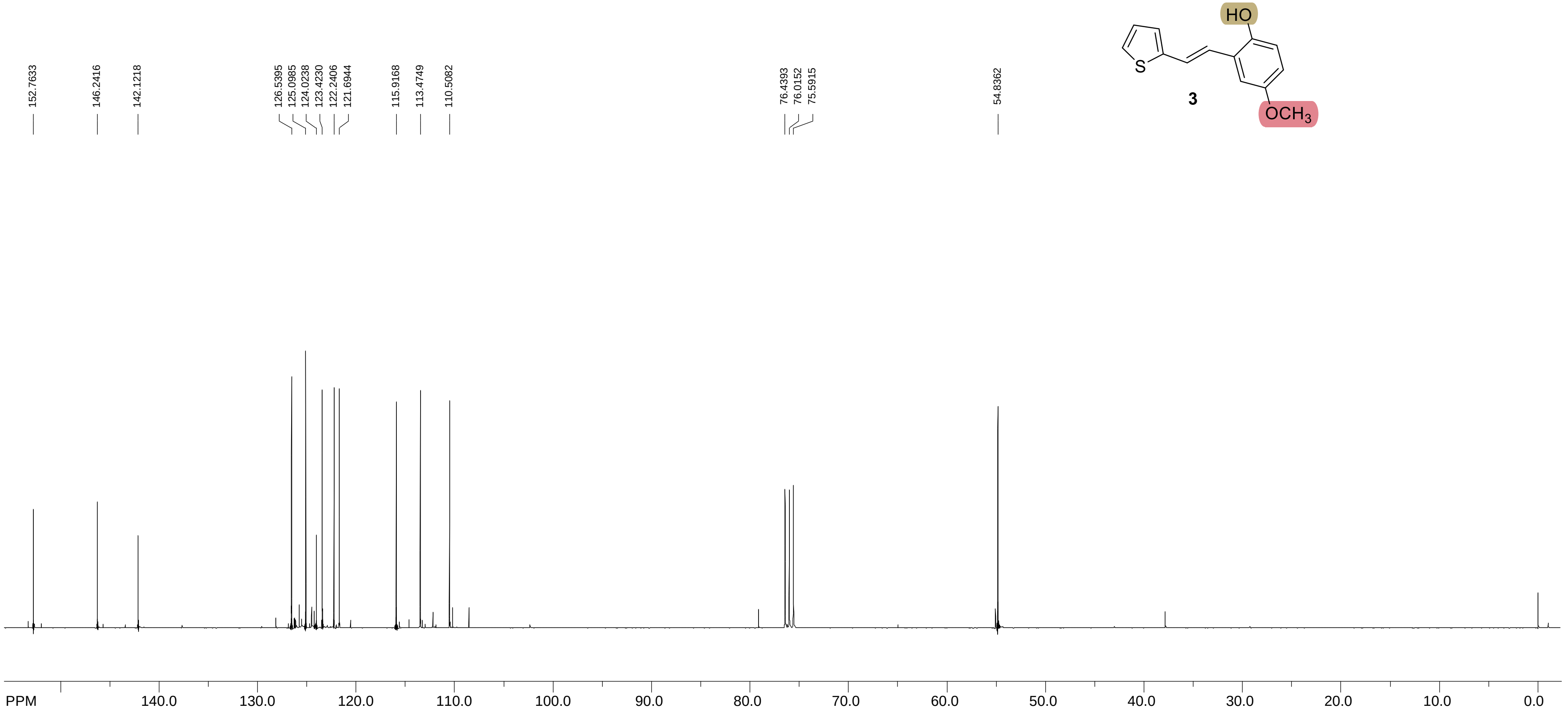


**Figure S13.** $^{13}C$ NMR ($CDCl_3$) spectrum of **3**.

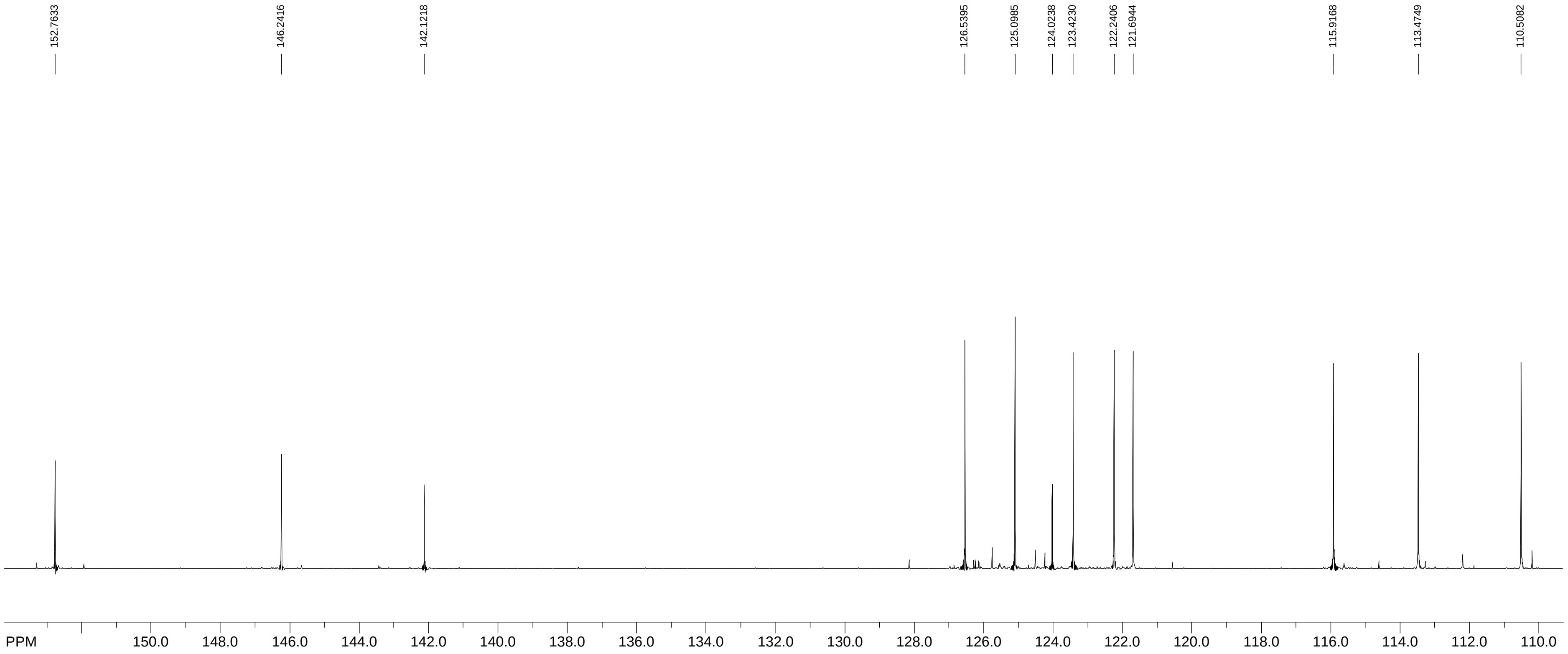


**Figure S14.** Aromatic part of $^{13}C$ NMR ($CDCl_3$) spectrum of **3**.

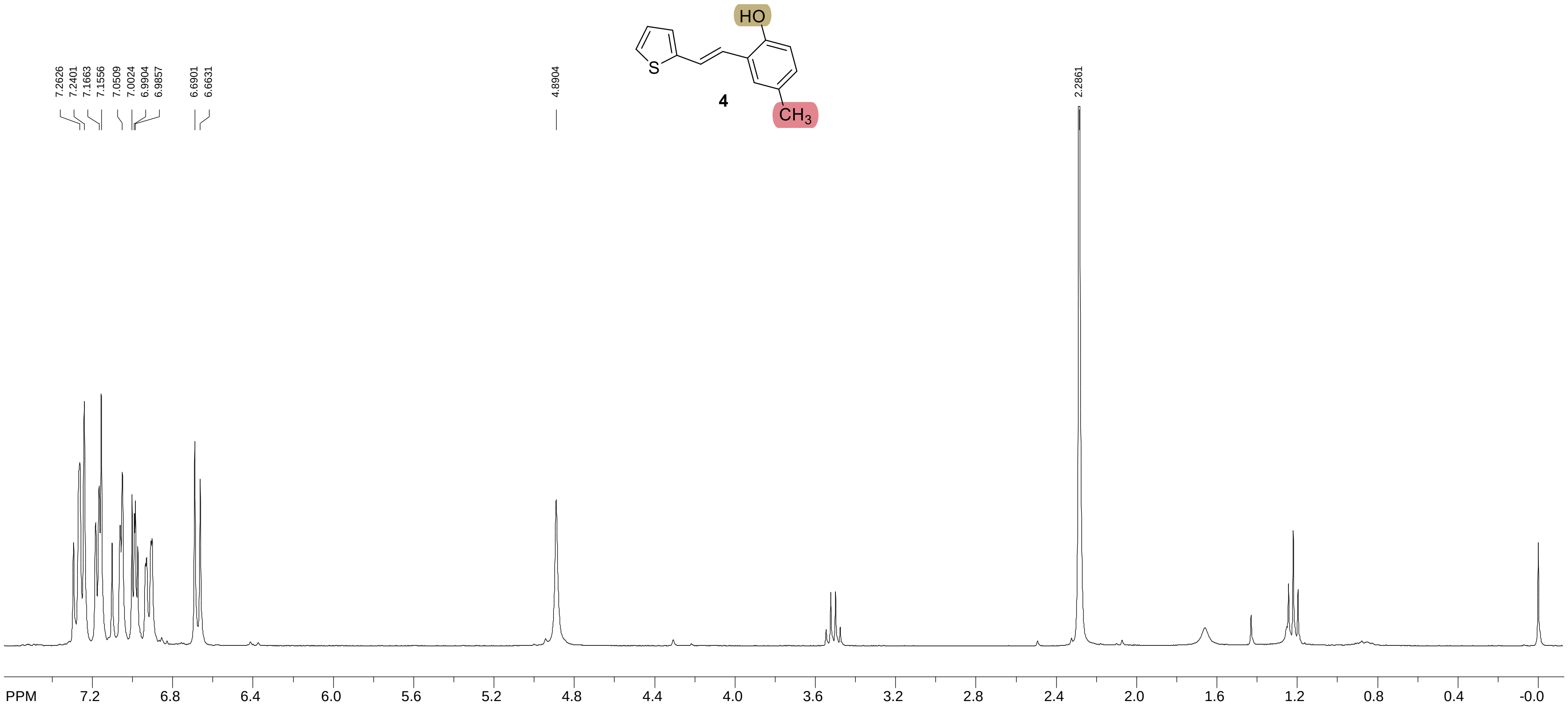


**Figure S15.** $^{1}H$ NMR ($CDCl_3$) spectrum of **4**.

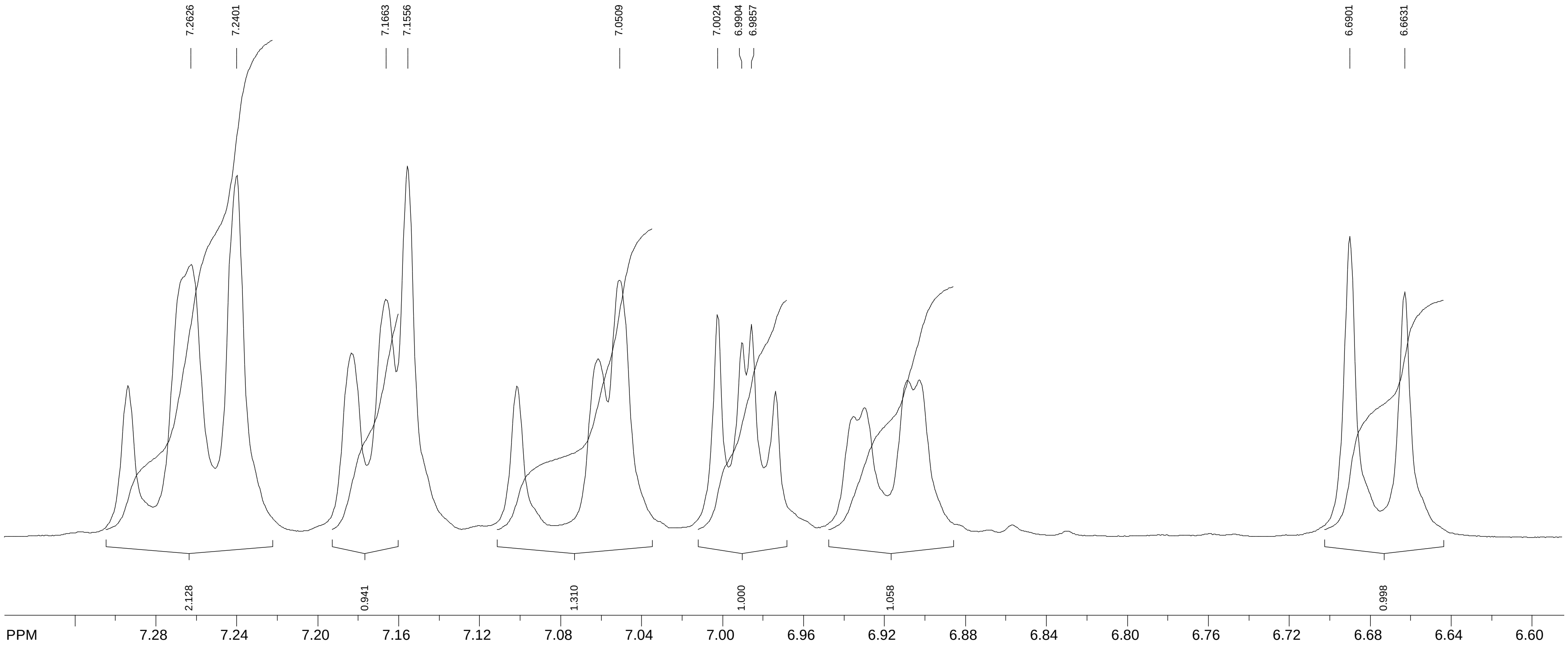


**Figure S16. A**romatic part of $^{1}$H NMR ($CDCl_3$) spectrum of **4**.

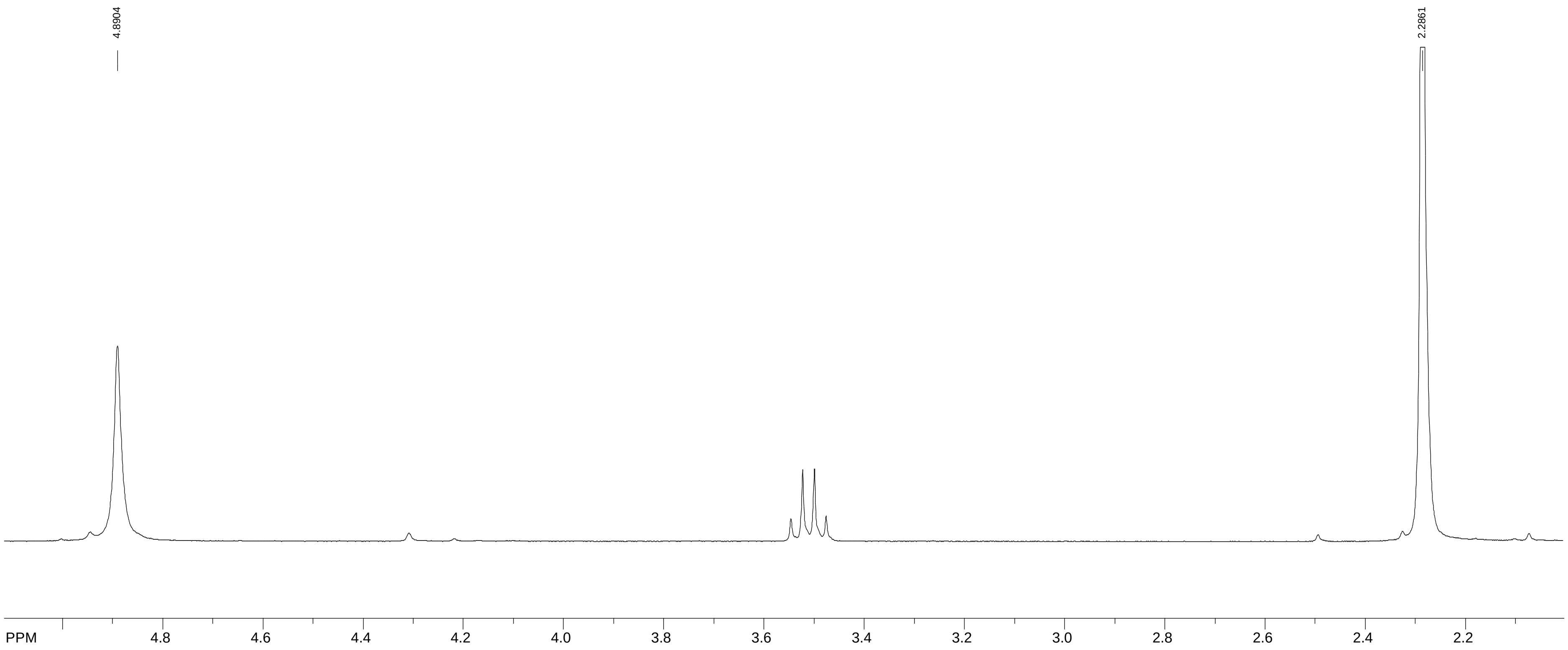


**Figure S17. A**liphatic part of $^1$H NMR ($CDCl_3$) spectrum of **4** (with a small amount of solvent ethanol).

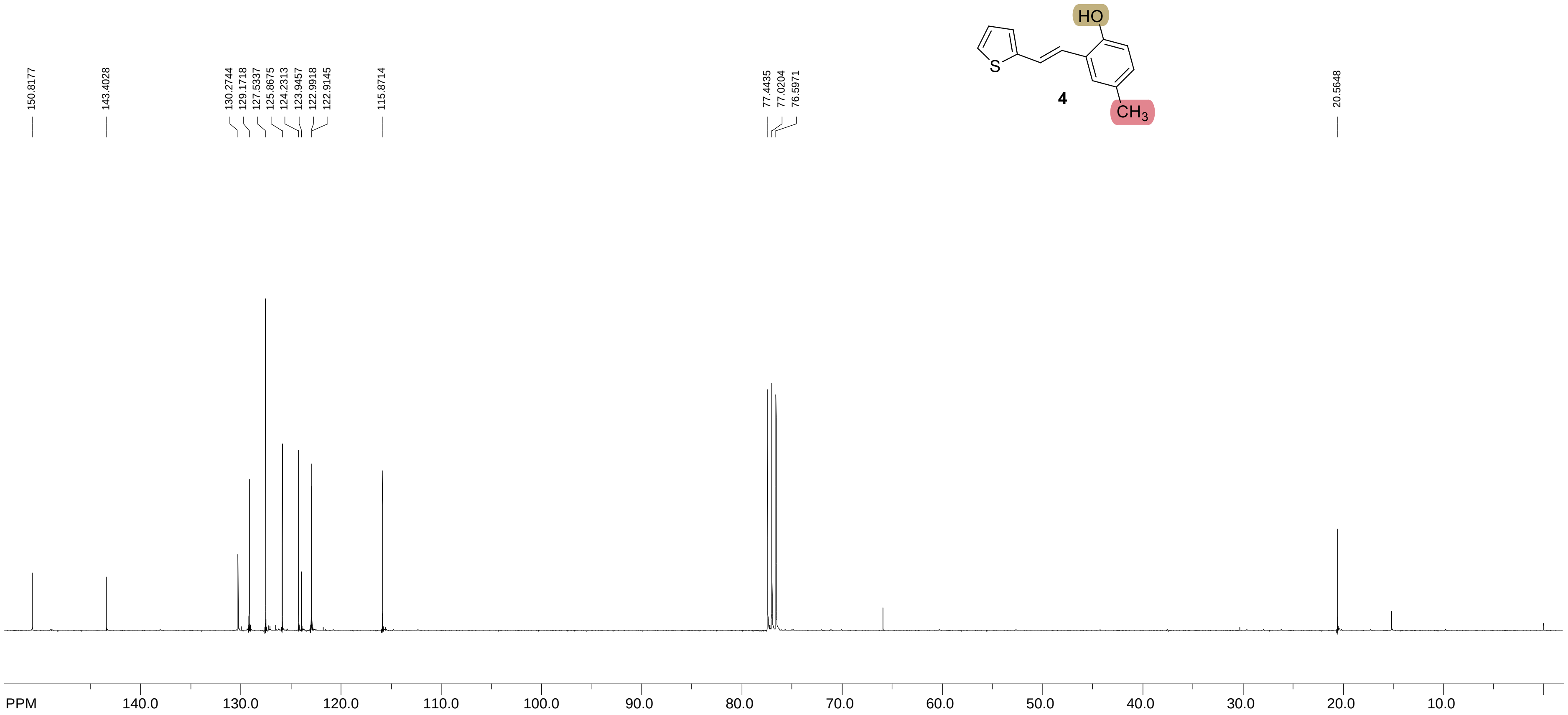


**Figure S18.** $^{13}$C NMR ($CDCl_3$) spectrum of **4**.

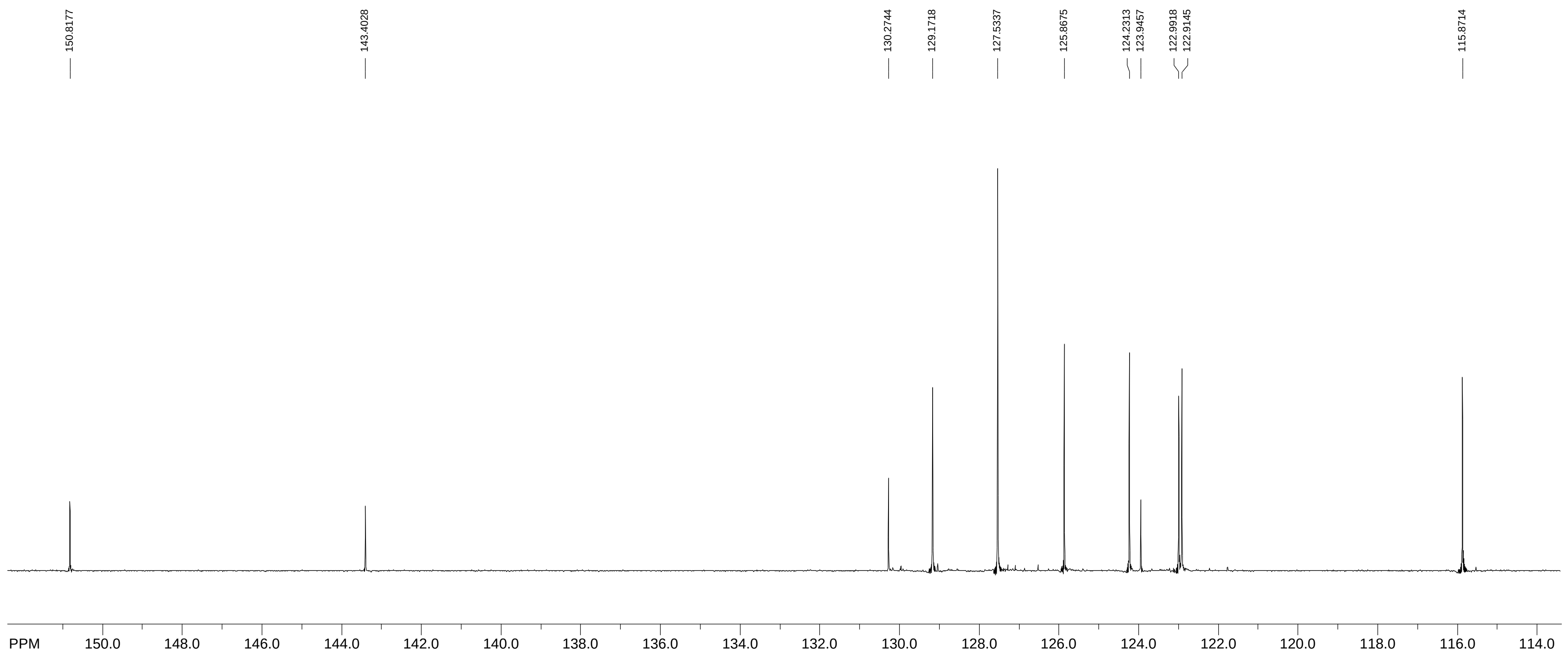


**Figure S19.** Aromatic part of $^{13}C$ NMR ($CDCl_3$) spectrum of **4**.

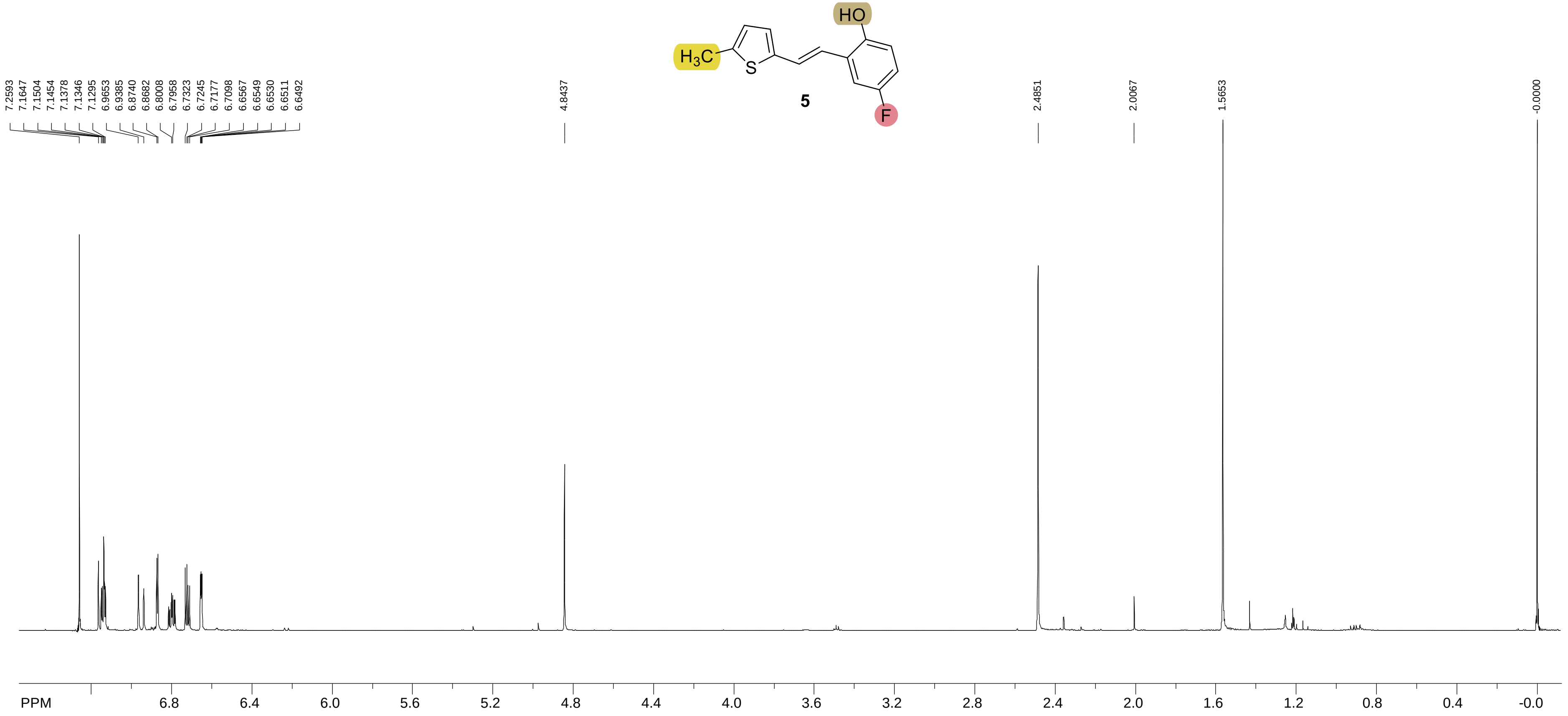


**Figure S20.** $^{1}H$ NMR ($CDCl_3$) spectrum of **5**.

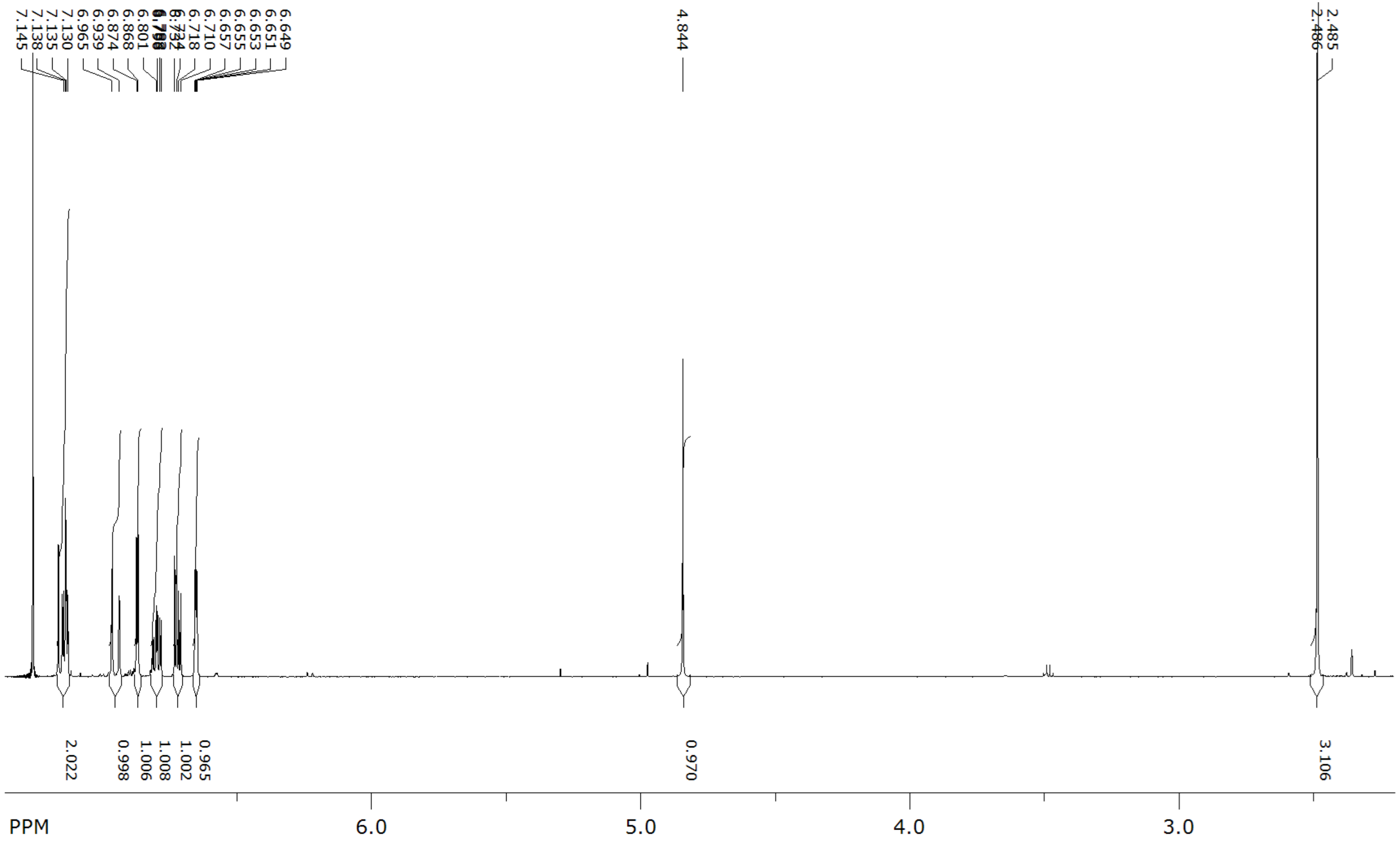


**Figure S21.** Part of $^{1}$H NMR ($CDCl_3$) spectrum of **5**.

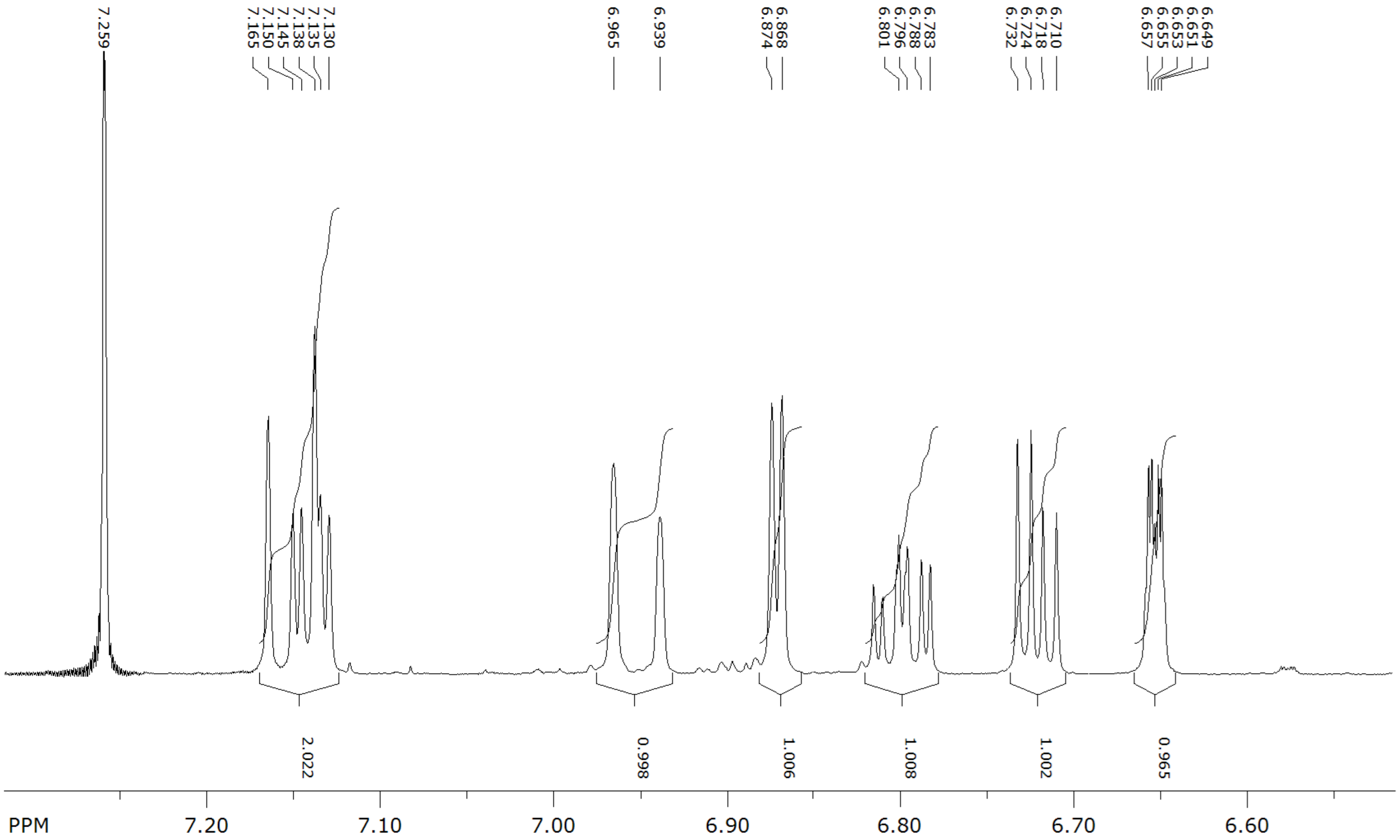


**Figure S22.** Aromatic part of $^{1}$H NMR ($CDCl_3$) spectrum of **5**.

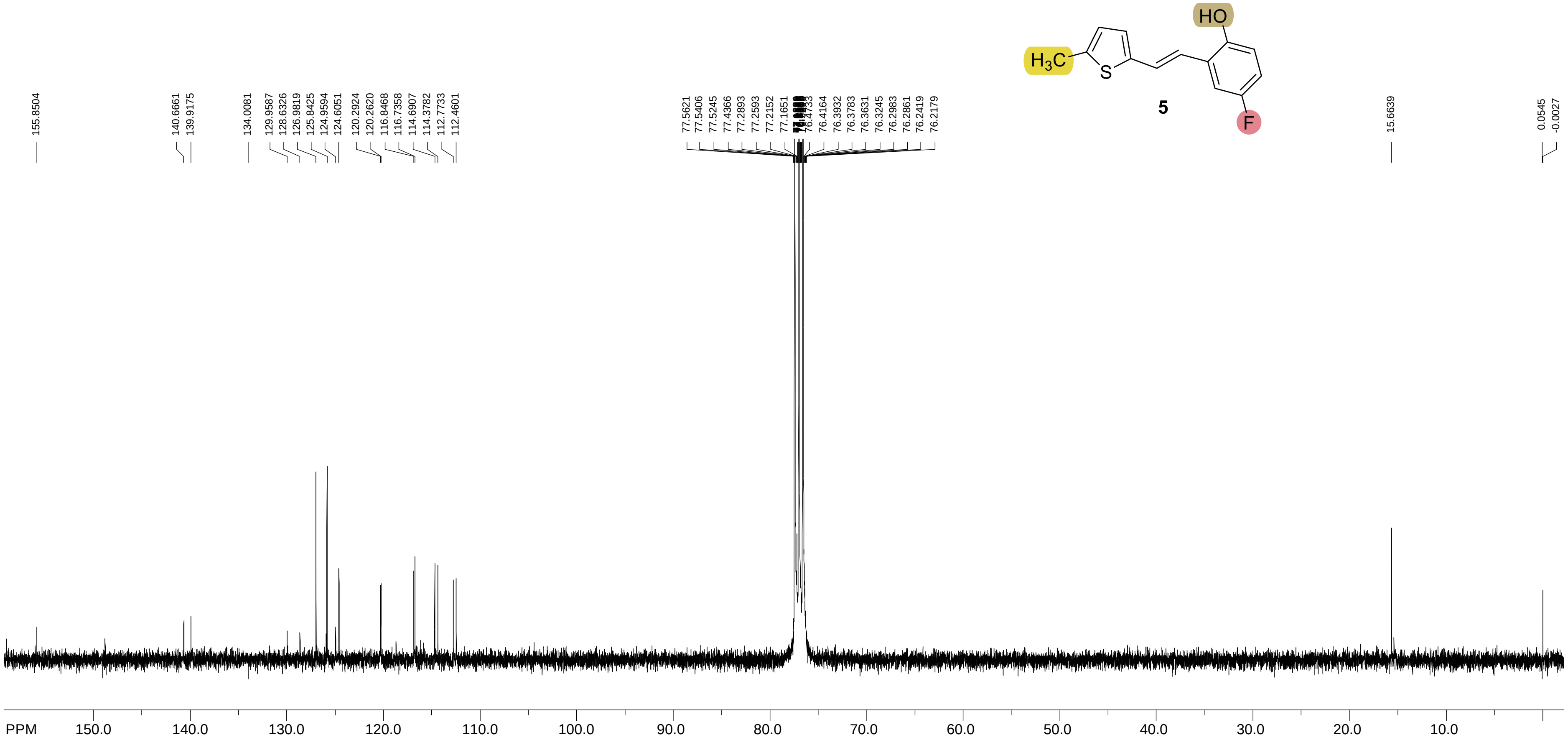


**Figure S23.** $^{13}C$ NMR ($CDCl_3$) spectrum of **5**.

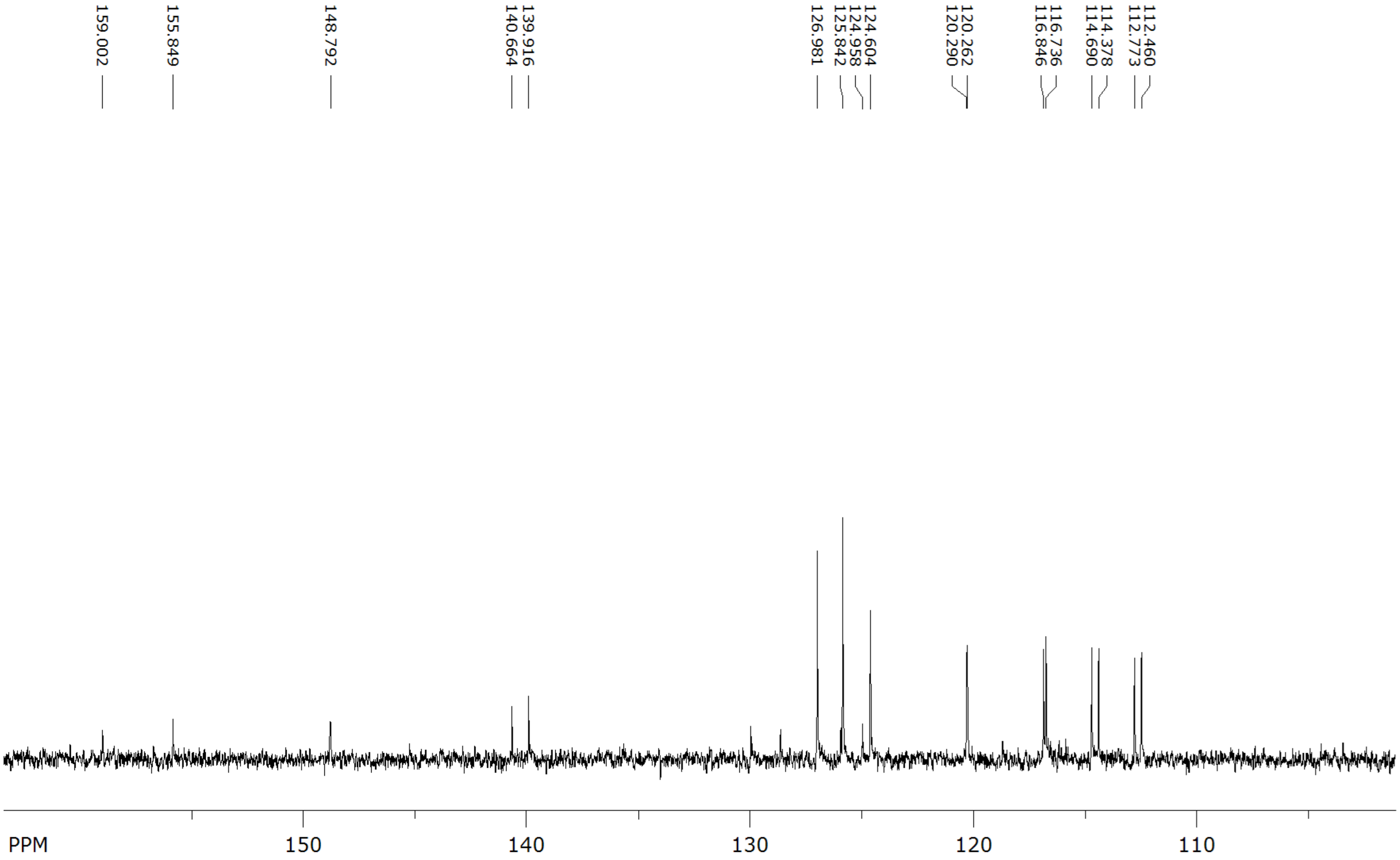


**Figure S24.** $^{13}C$ NMR ($CDCl_3$) aromatic part of spectrum of **5**.

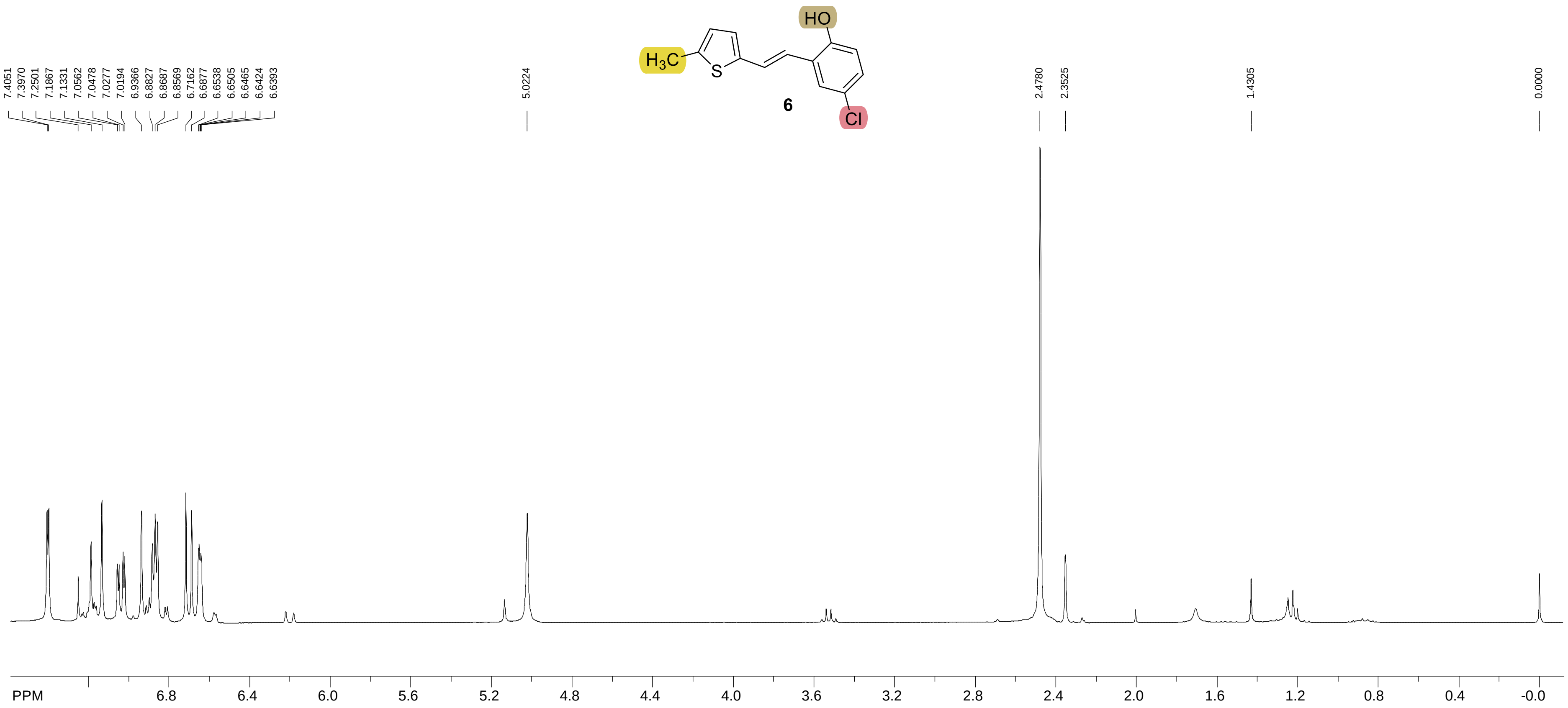


**Figure S25.** $^{1}H$ NMR ($CDCl_3$) spectrum of **6**.

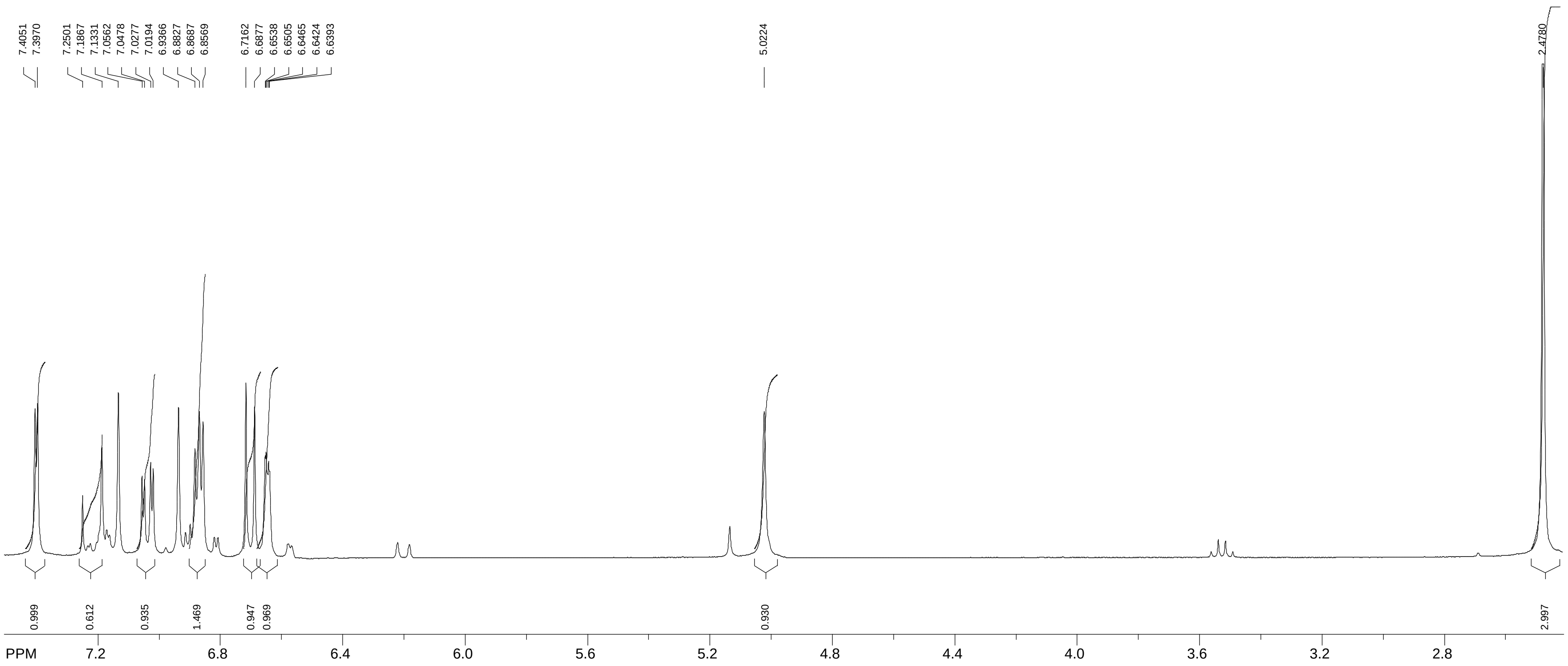


**Figure S26.** Part of $^{1}$H NMR ($CDCl_3$) spectrum of **6**.

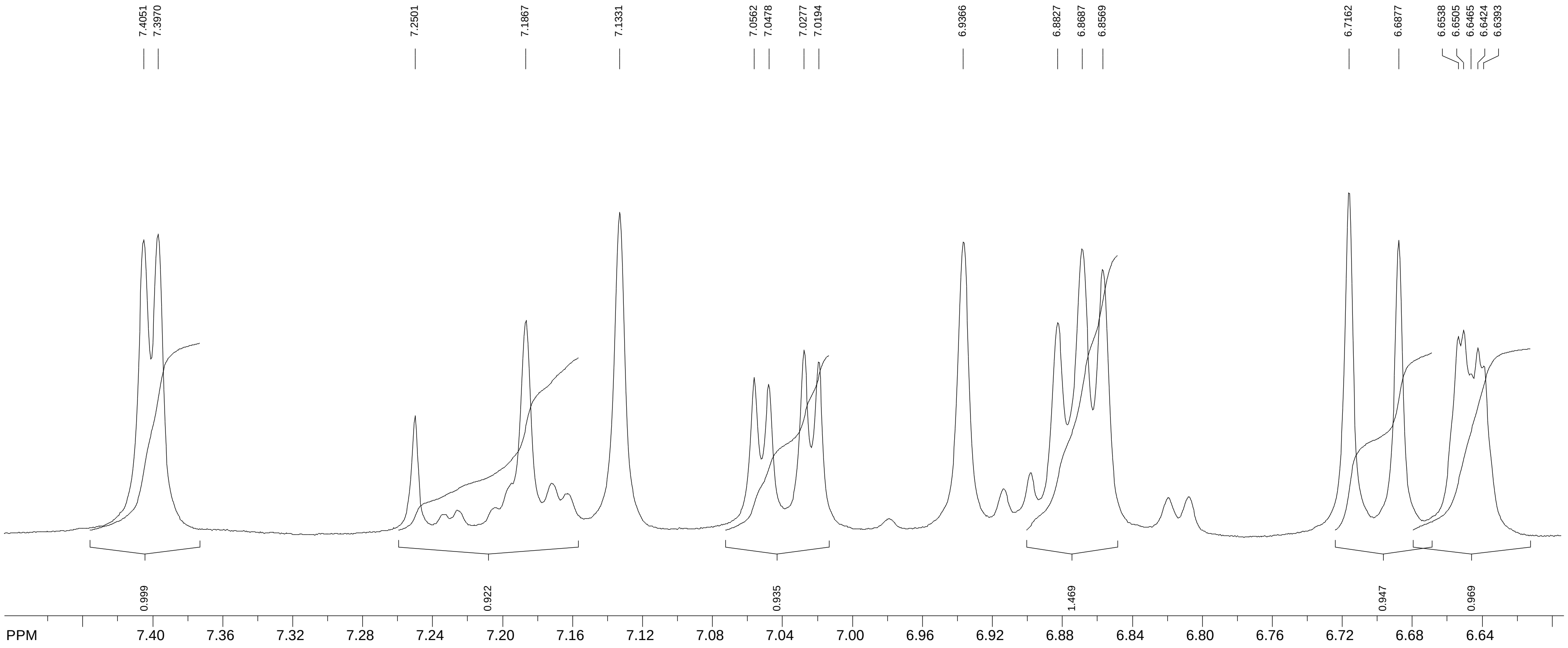


**Figure S27.** Aromatic part of $^1$H NMR ($CDCl_3$) spectrum of **6**.

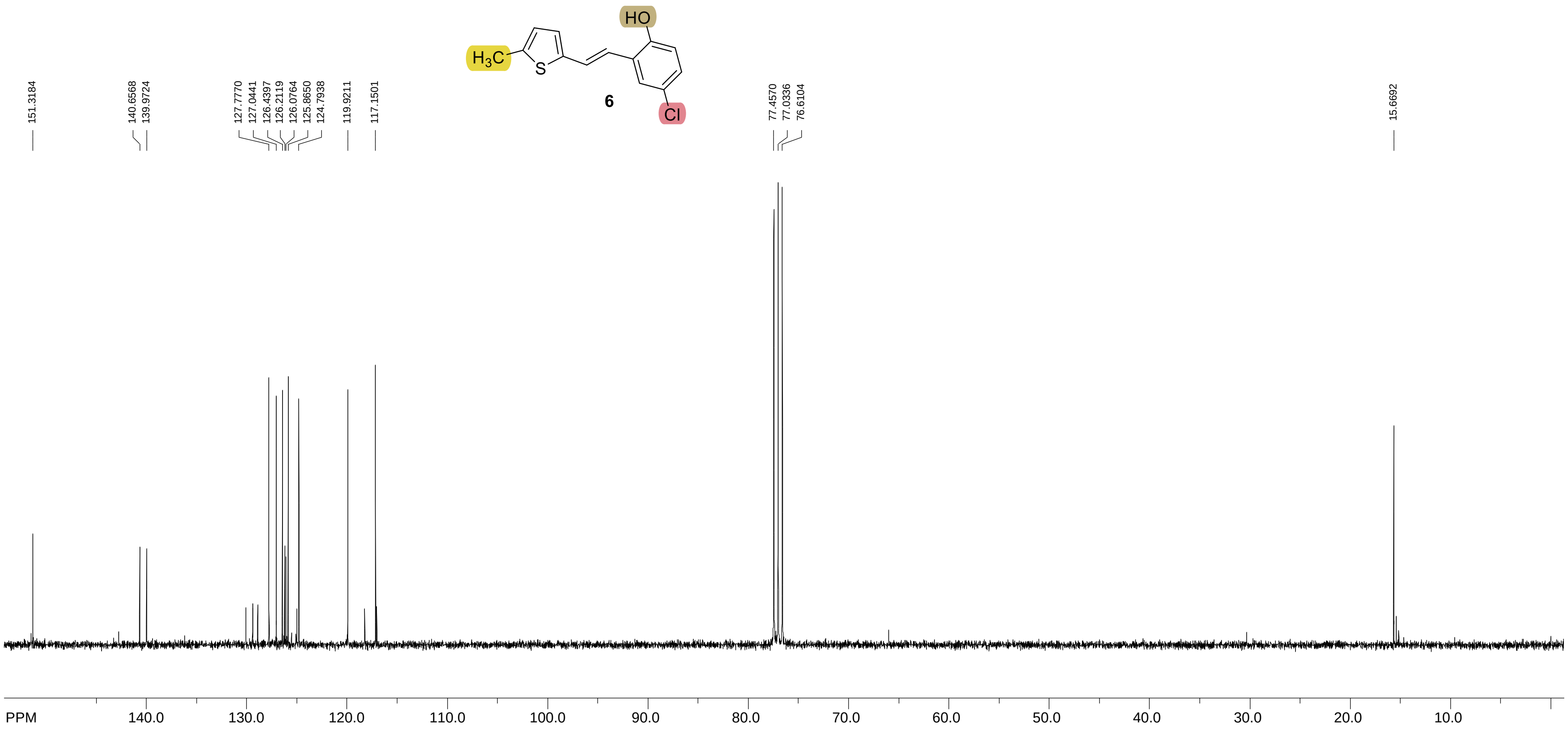


**Figure S28.** $^{13}C$ NMR ($CDCl_3$) spectrum of **6**.

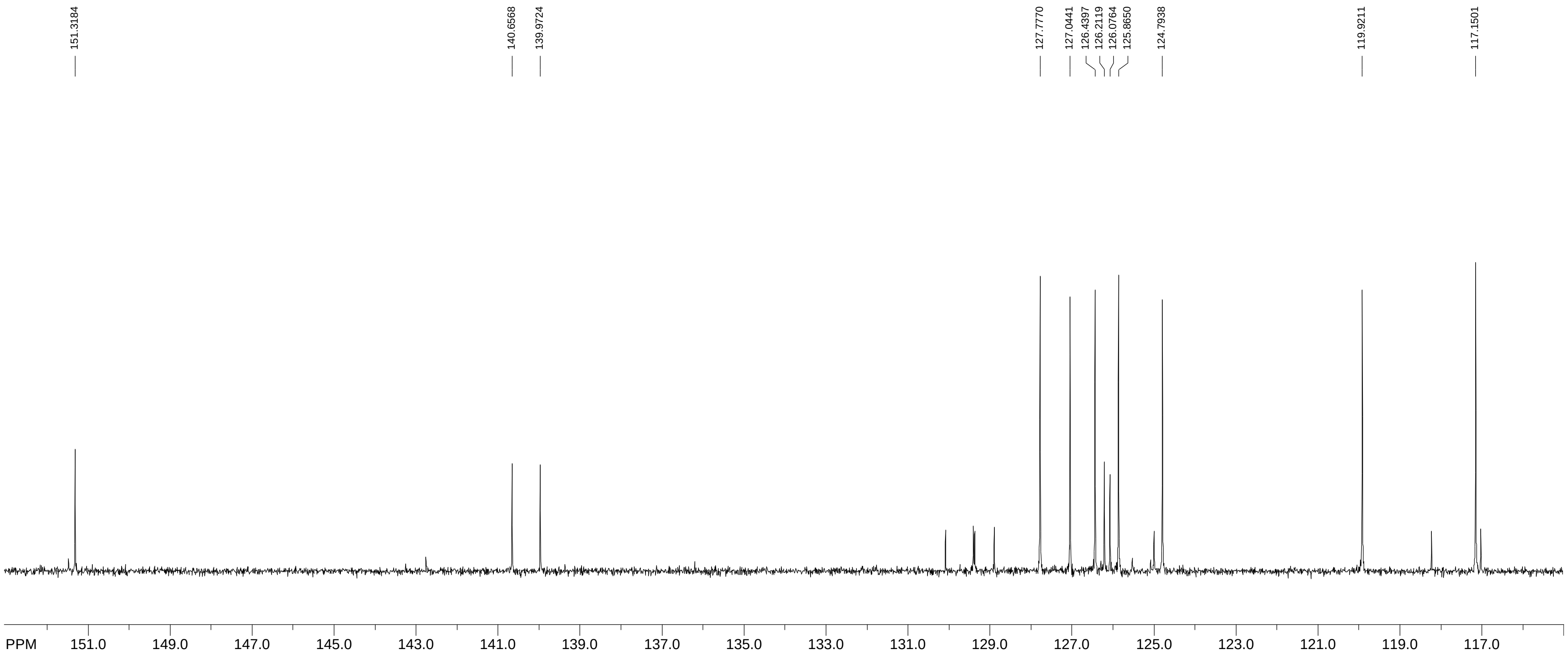


**Figure S29.** Aromatic part of $^{13}C$ NMR ($CDCl_3$) spectrum of **6**.

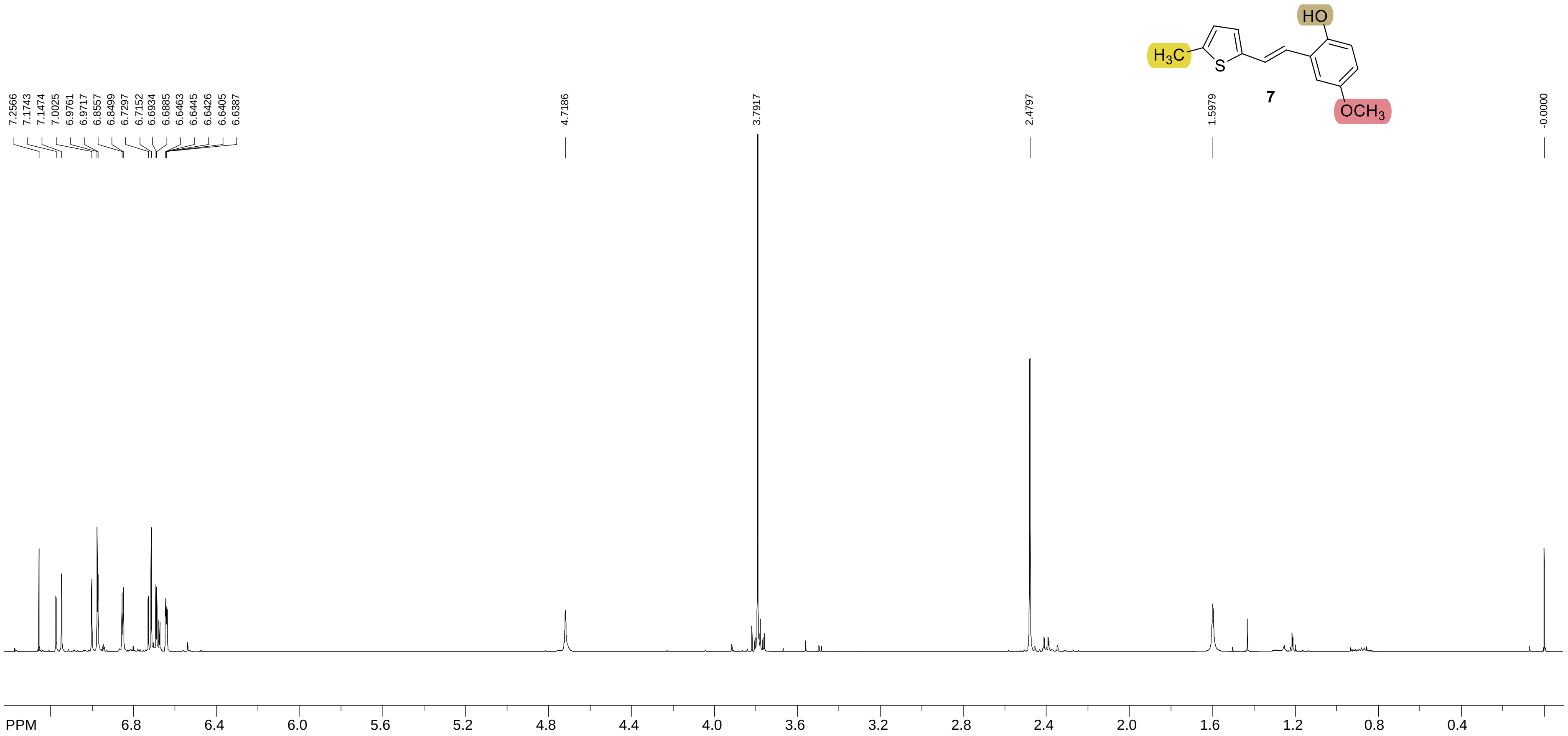


**Figure S30.** $^1$H NMR ($CDCl_3$) spectrum of **7**.

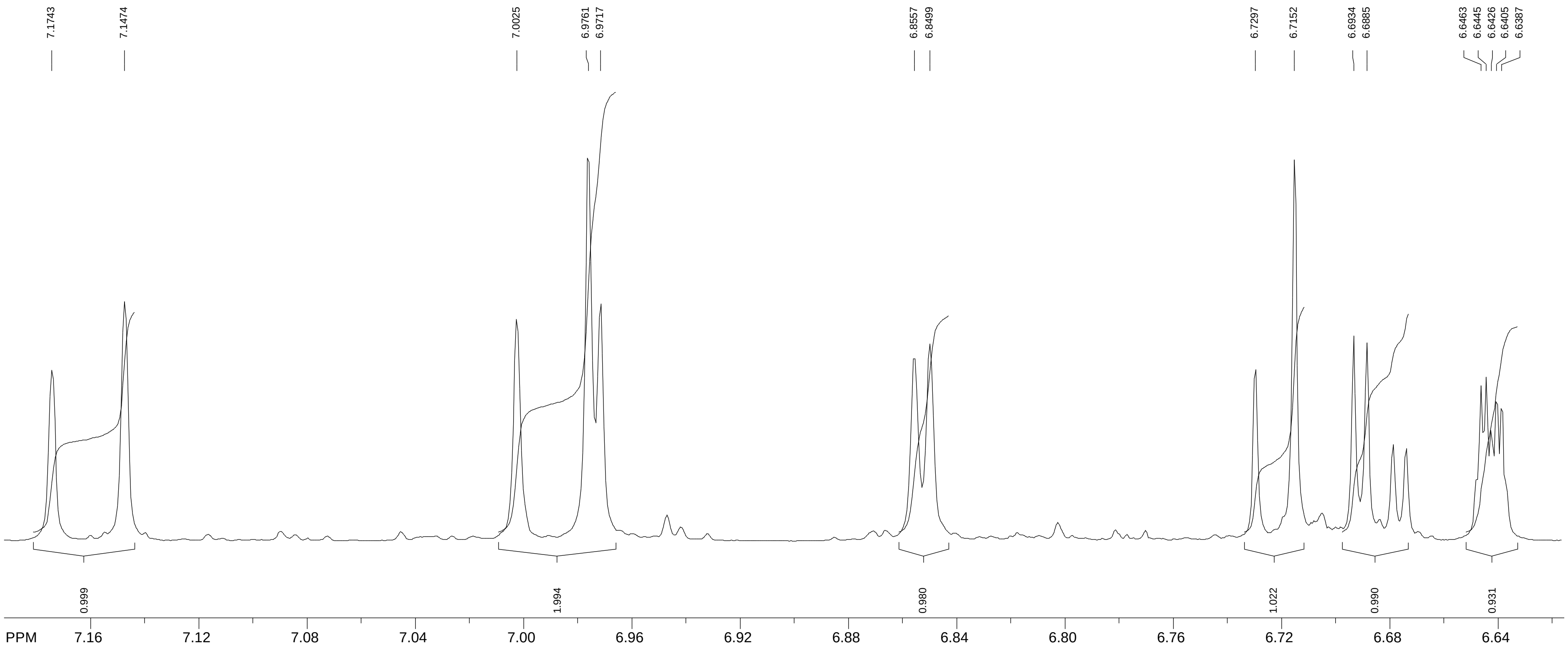


**Figure S31.** Aromatic part of $^{1}$H NMR ($CDCl_3$) spectrum of **7**.

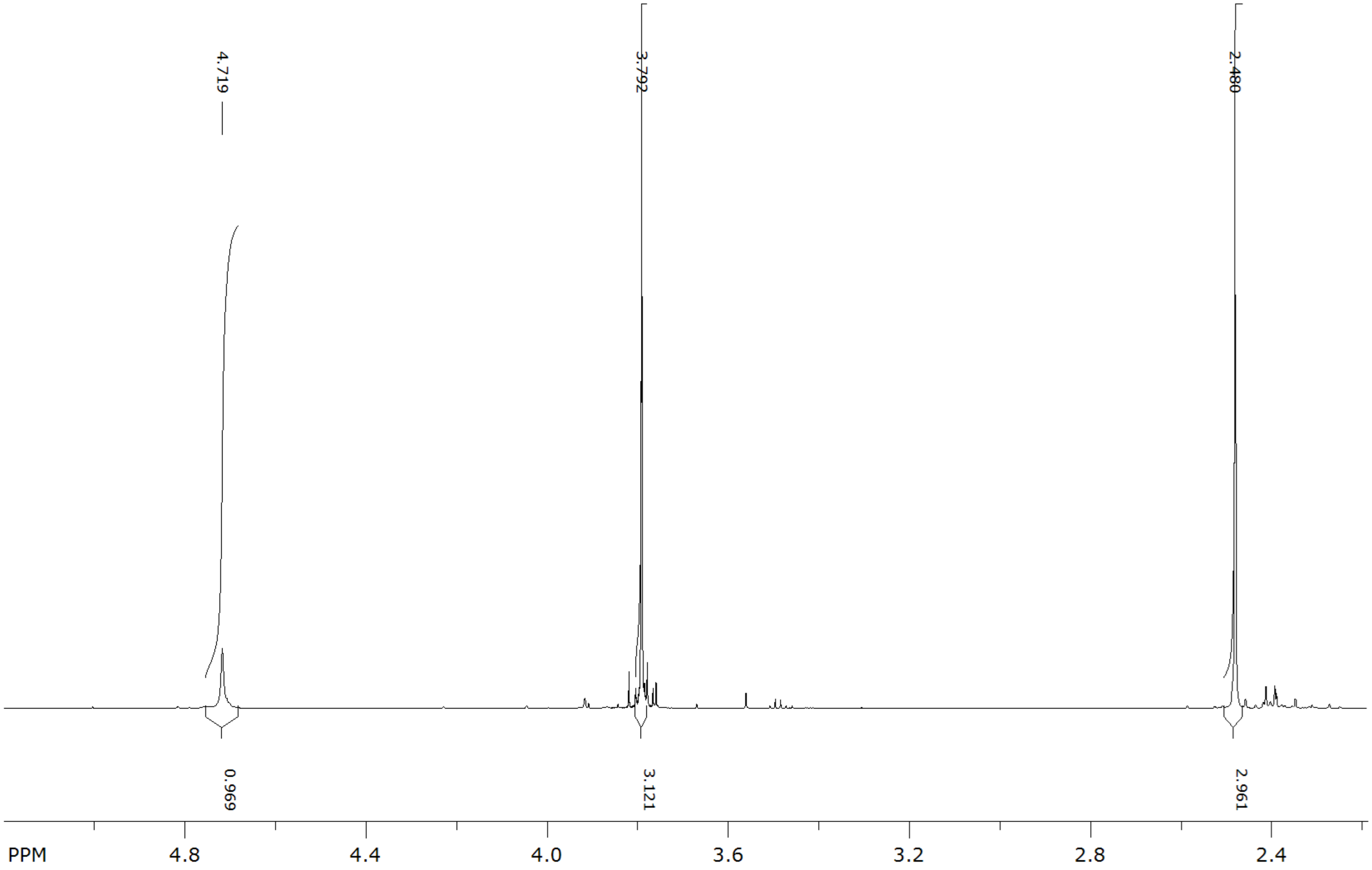


**Figure S32.** Aliphatic part of $^{1}H$ NMR ($CDCl_3$) spectrum of **7**.

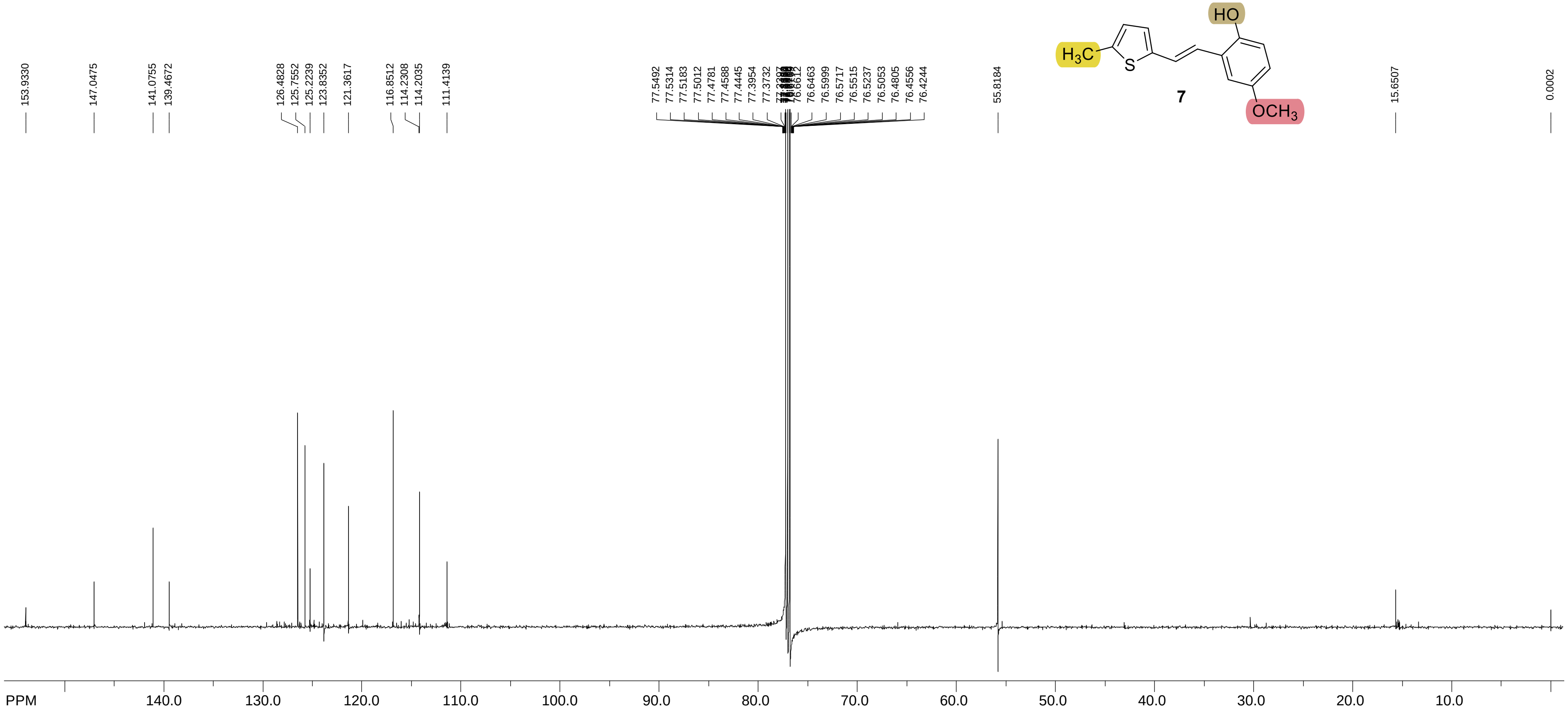


**Figure S33.** $^{13}C$ NMR ($CDCl_3$) spectrum of **7**.

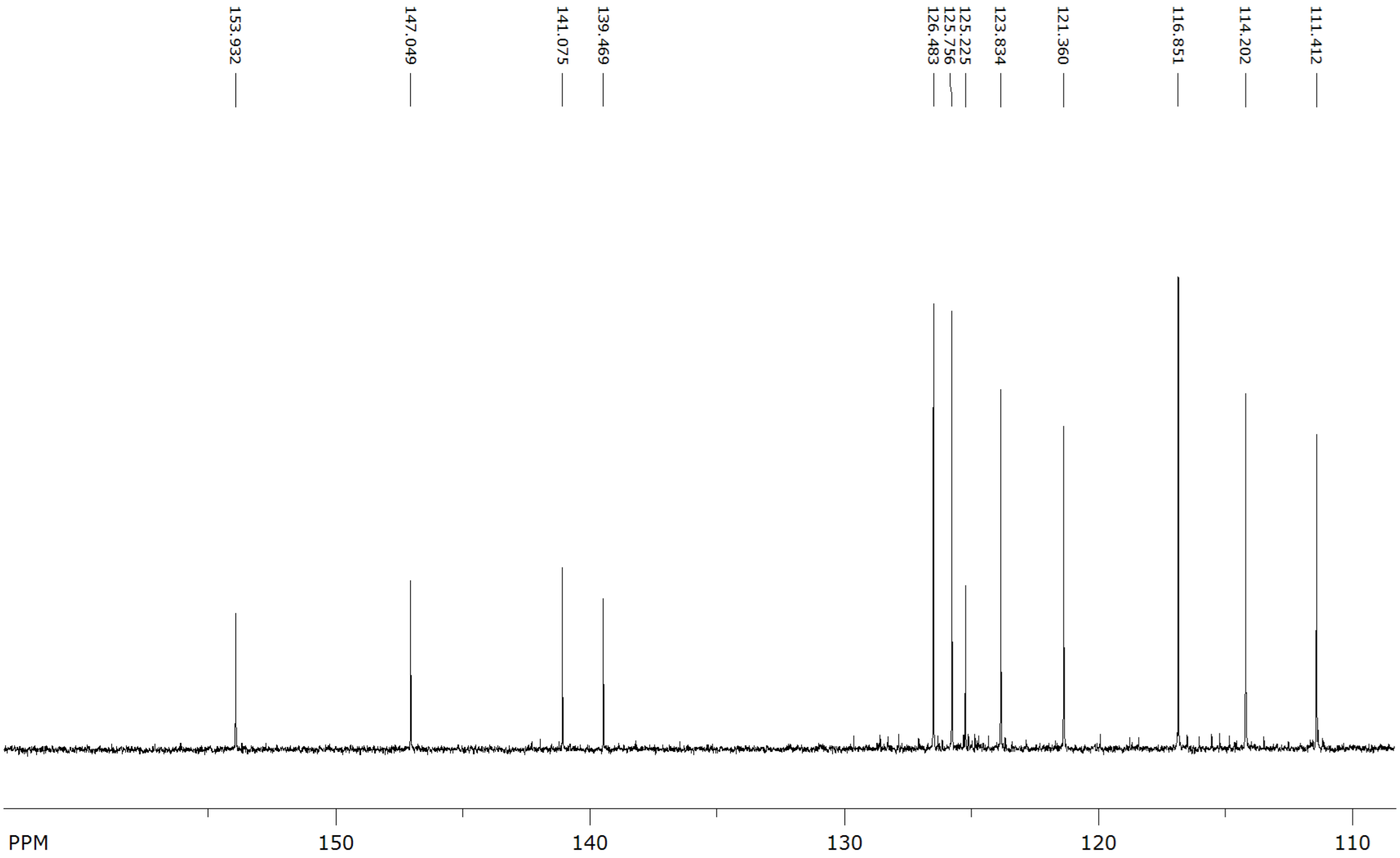


**Figure S34.** Aromatic part of $^{13}C$ NMR ($CDCl_3$) spectrum of **7**.

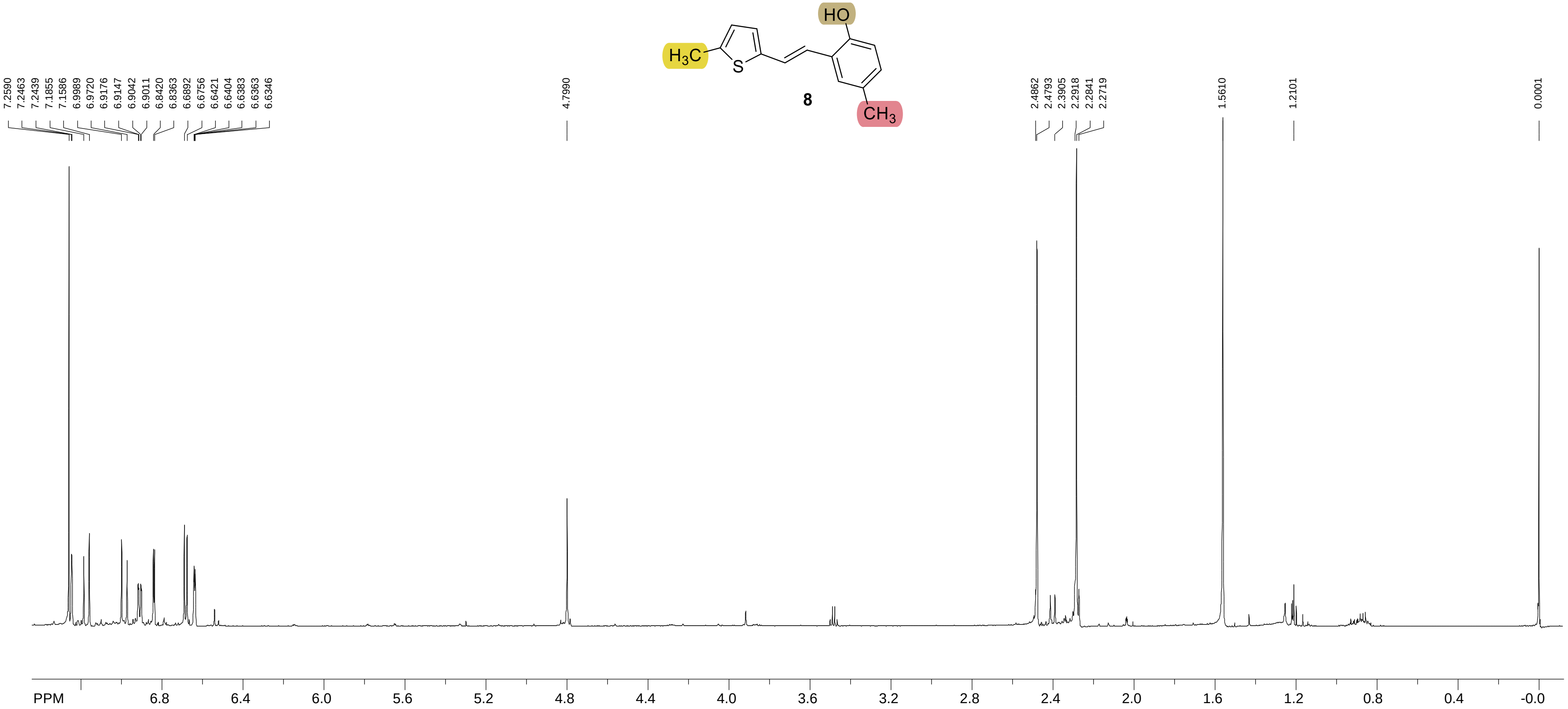


**Figure S35.** $^{1}$H NMR ($CDCl_3$) spectrum of **8**.

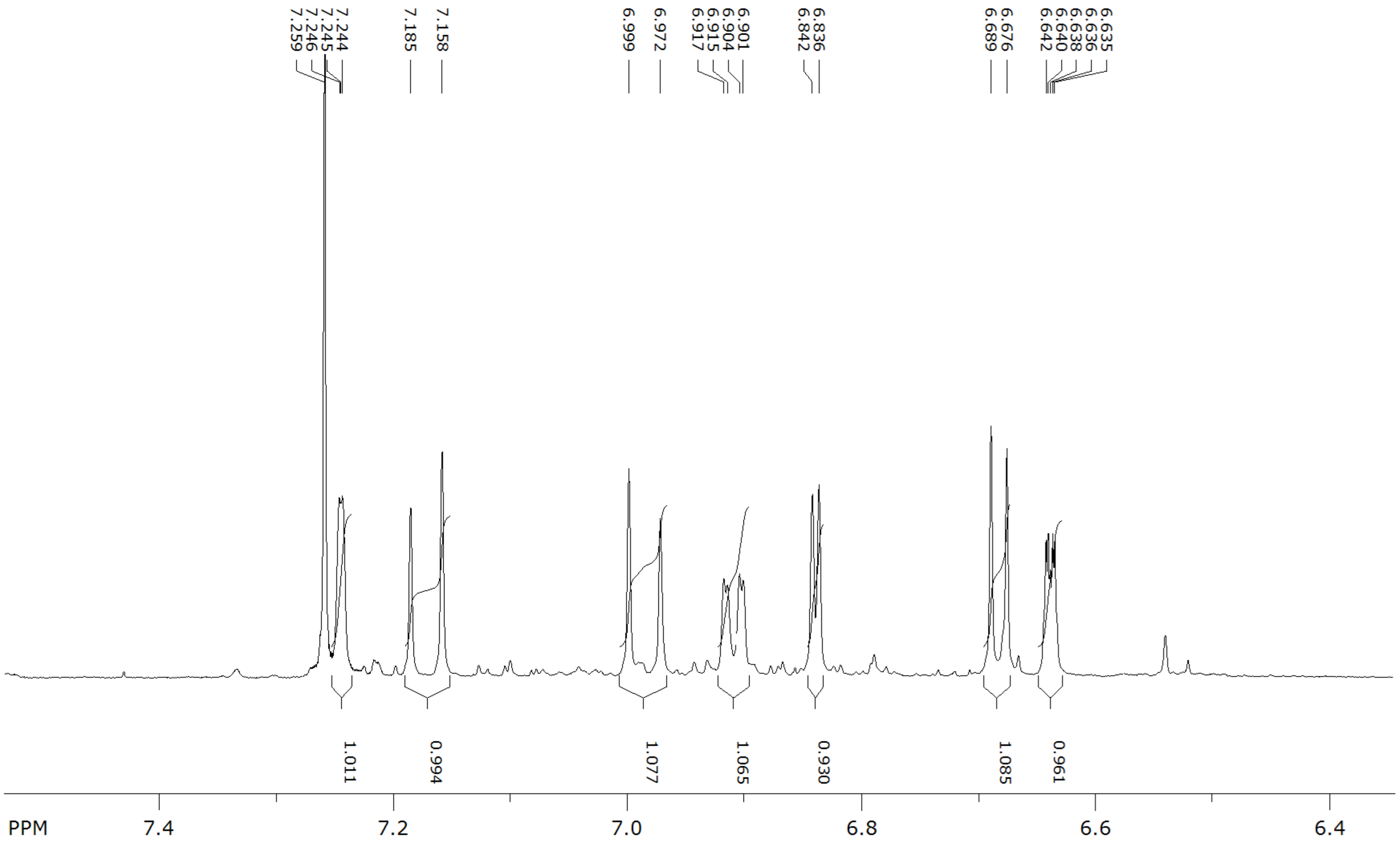


**Figure S36.** Aromatic part of $^1$H NMR ($CDCl_3$) spectrum of **8**.

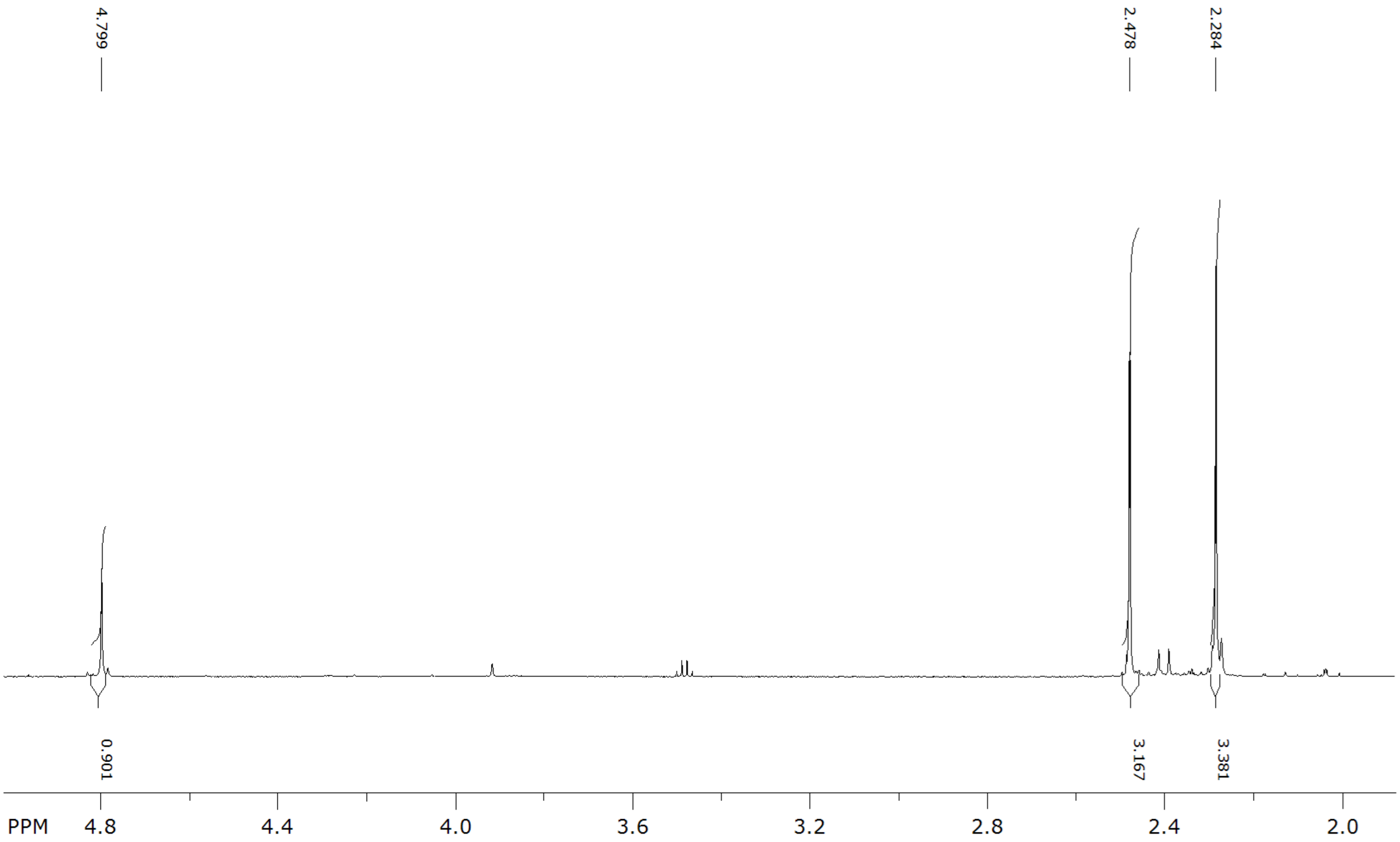


**Figure S37.** Aliphatic part of $^{1}$H NMR ($CDCl_3$) spectrum of **8** (with a small amount of solvent ethanol).

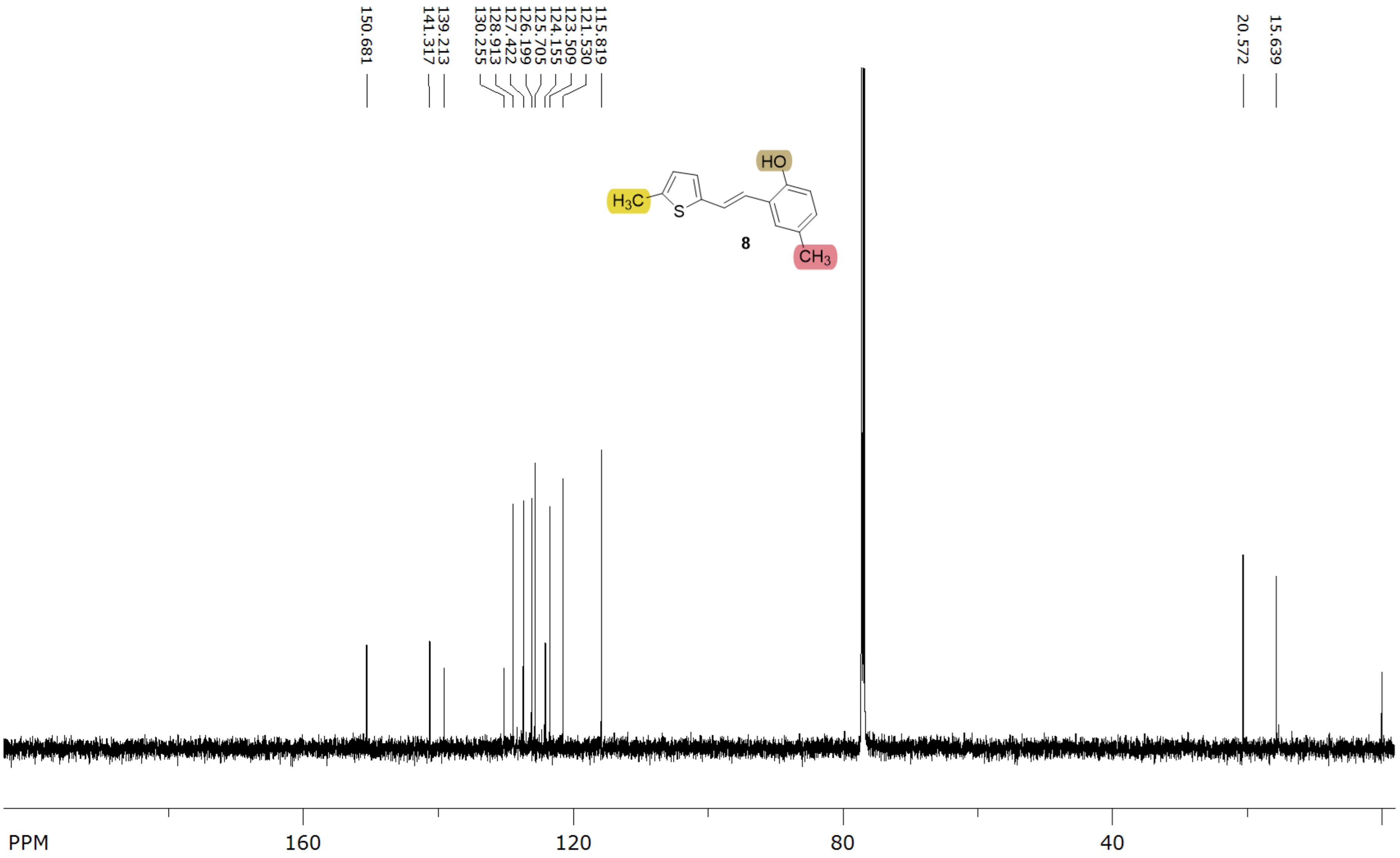


**Figure S38.** $^{13}C$ NMR ($CDCl_3$) spectrum of **8**.

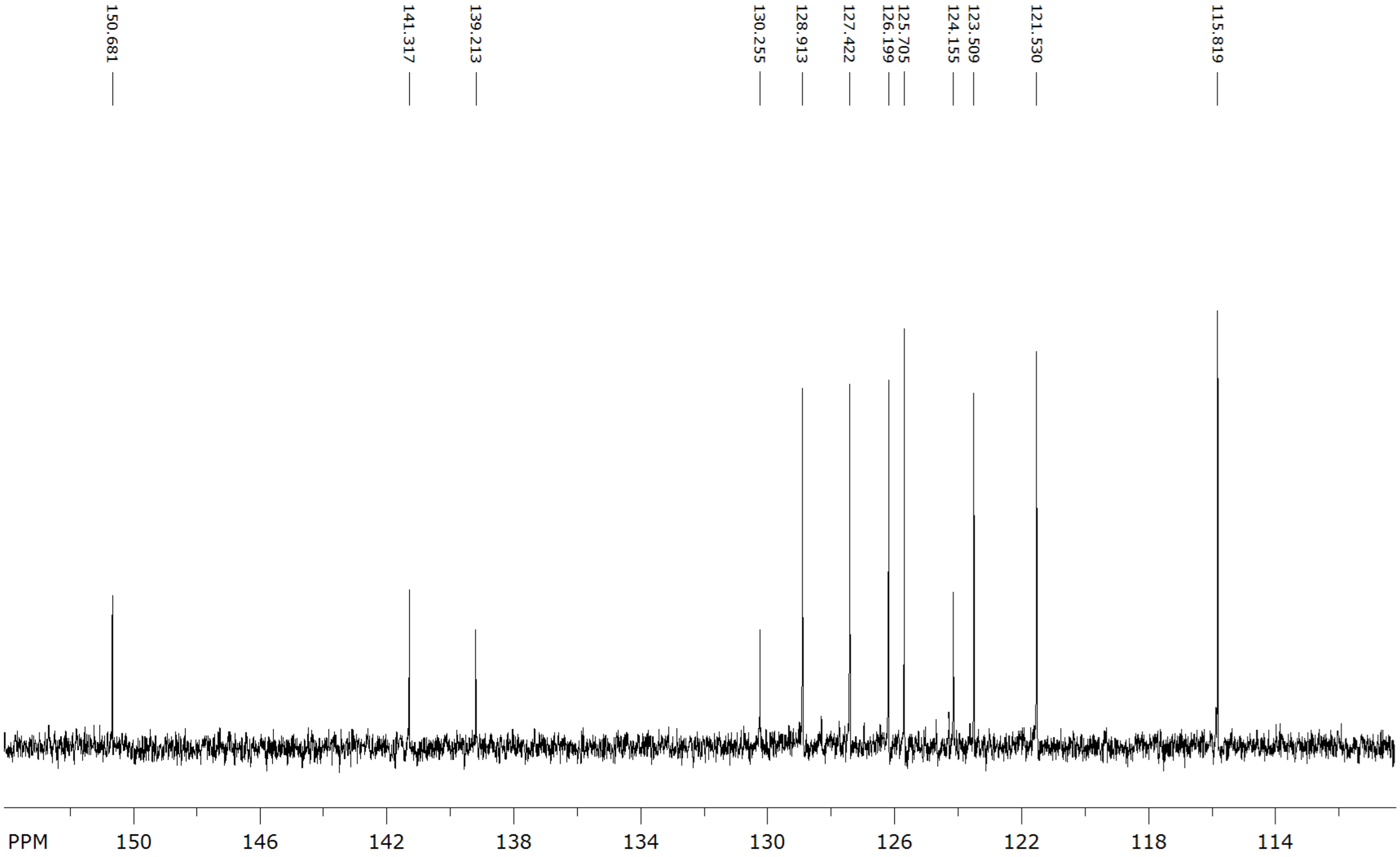


**Figure S39.** Aromatic part of $^{13}C$ NMR ($CDCl_3$) spectrum of **8**.

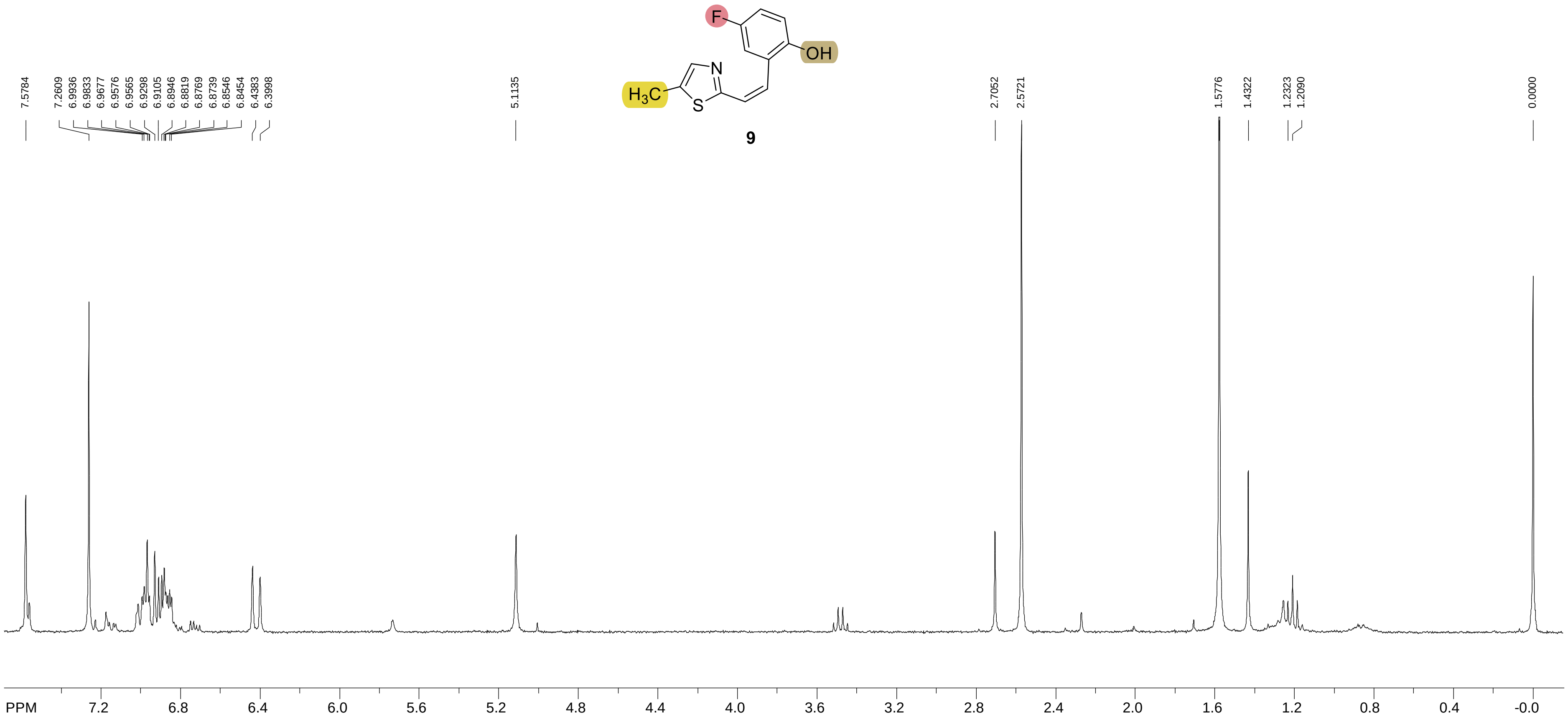


**Figure S40.** $^1H$ NMR ($CDCl_3$) spectrum of **9**.

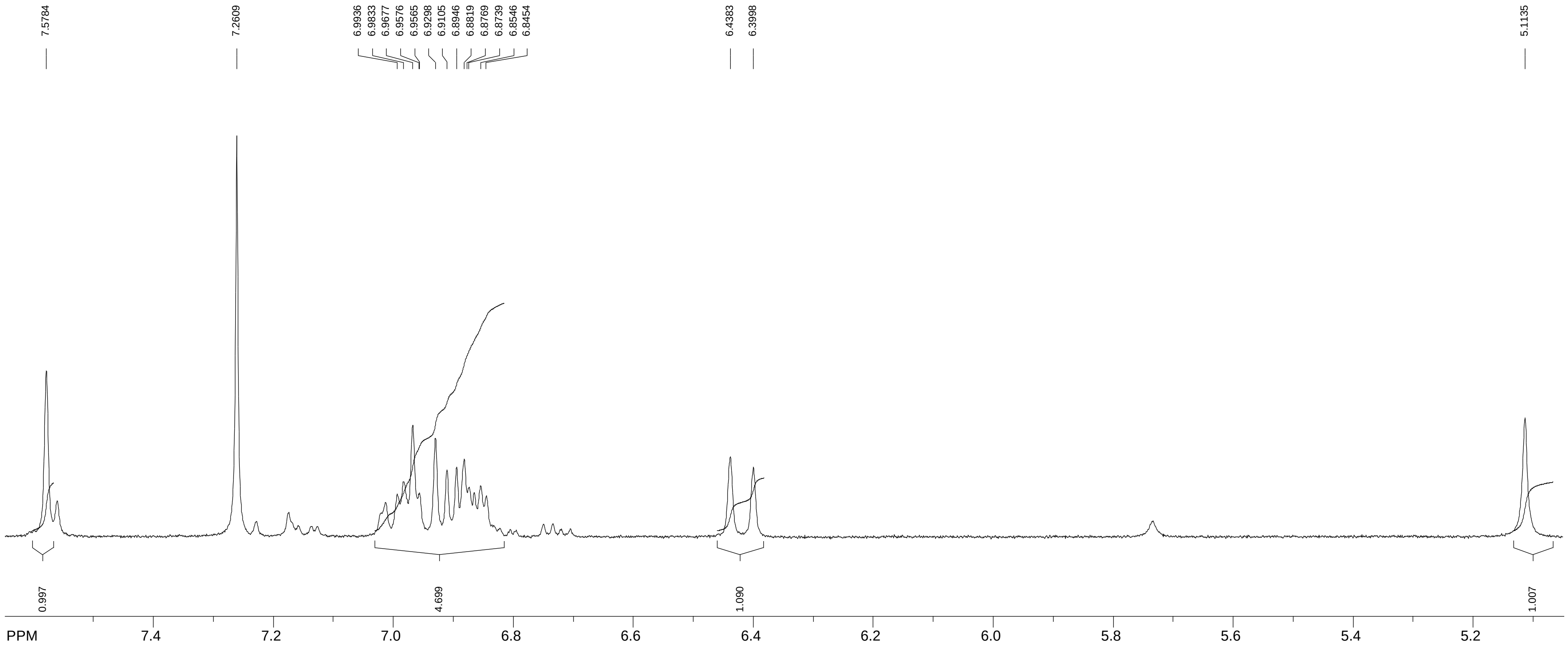


**Figure S41.** Aromatic part of $^{1}H$ NMR ($CDCl_3$) spectrum of **9**.

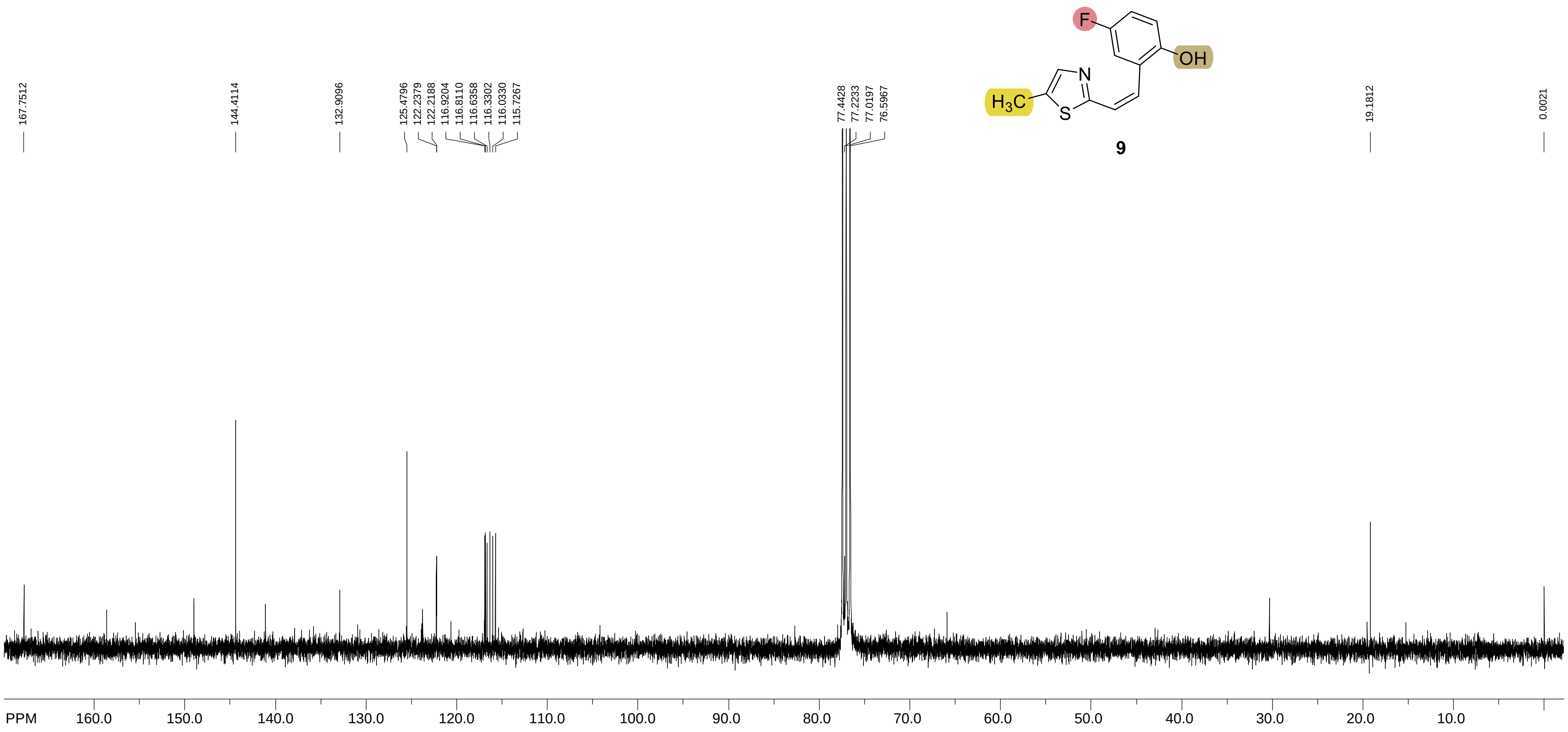


**Figure S42.** $^{13}$C NMR ($CDCl_3$) spectrum of **9**.

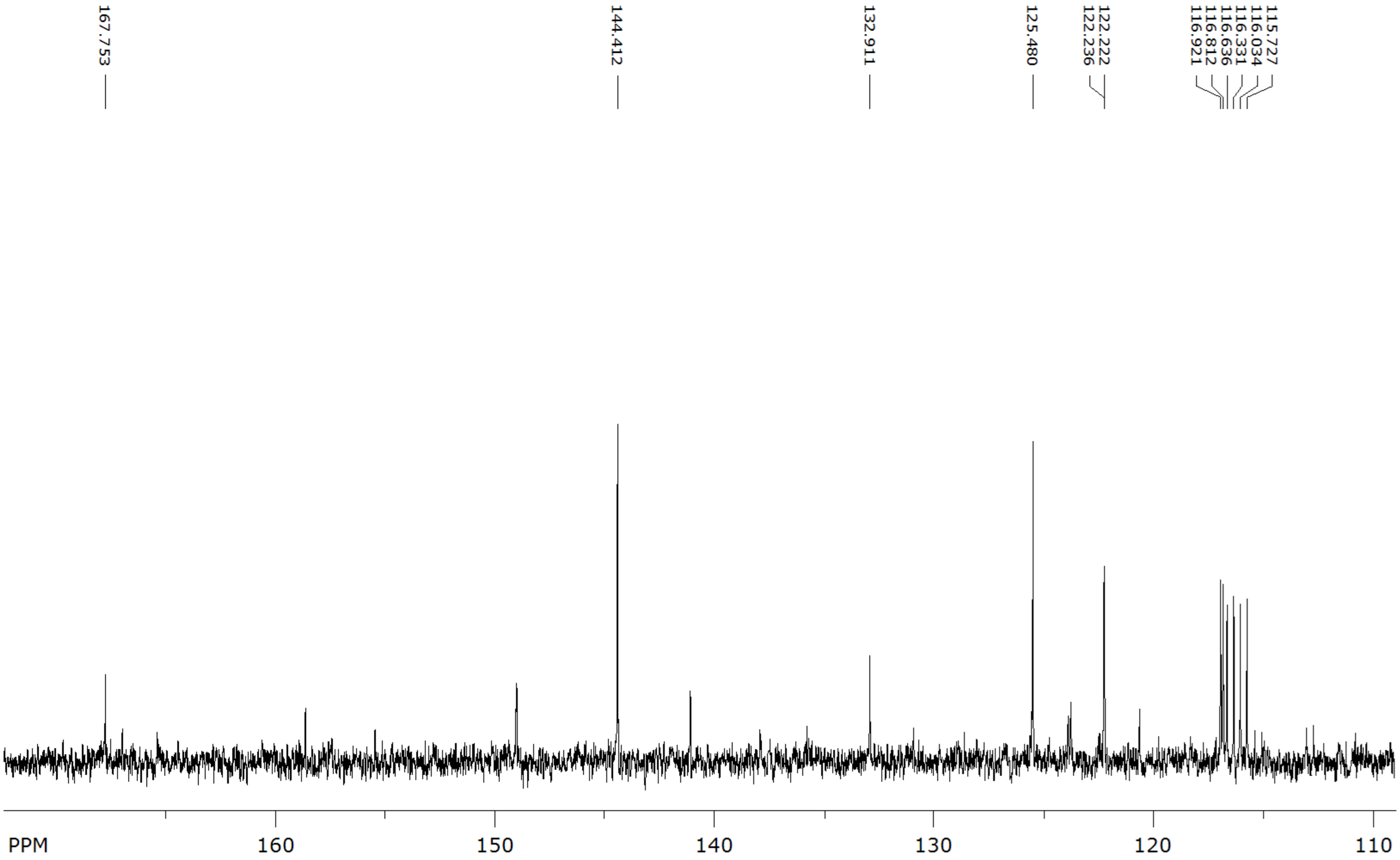


**Figure S43.** $^{13}C$ NMR ($CDCl_3$) aromatic part of spectrum of **9**.

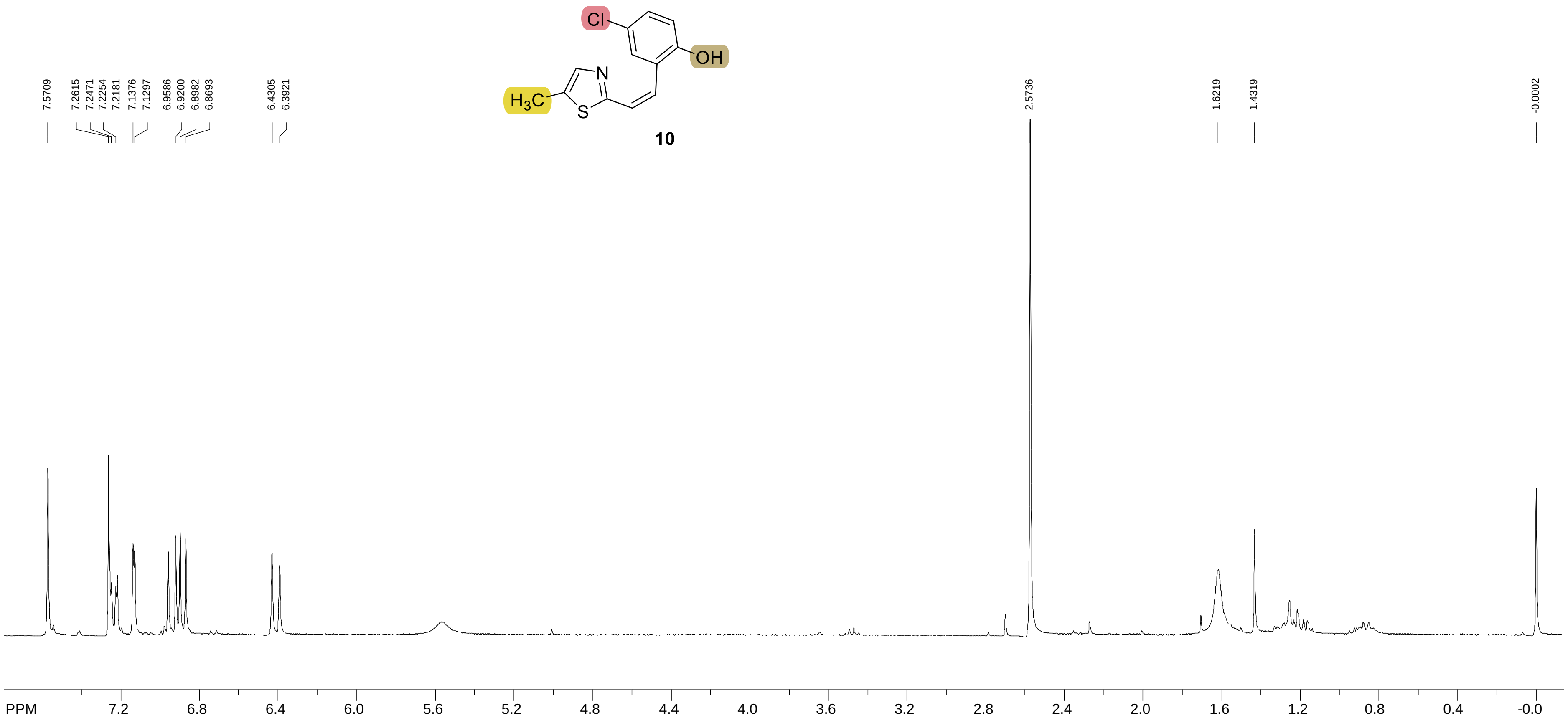


**Figure S44.** $^{1}$H NMR ($CDCl_3$) spectrum of **10**.

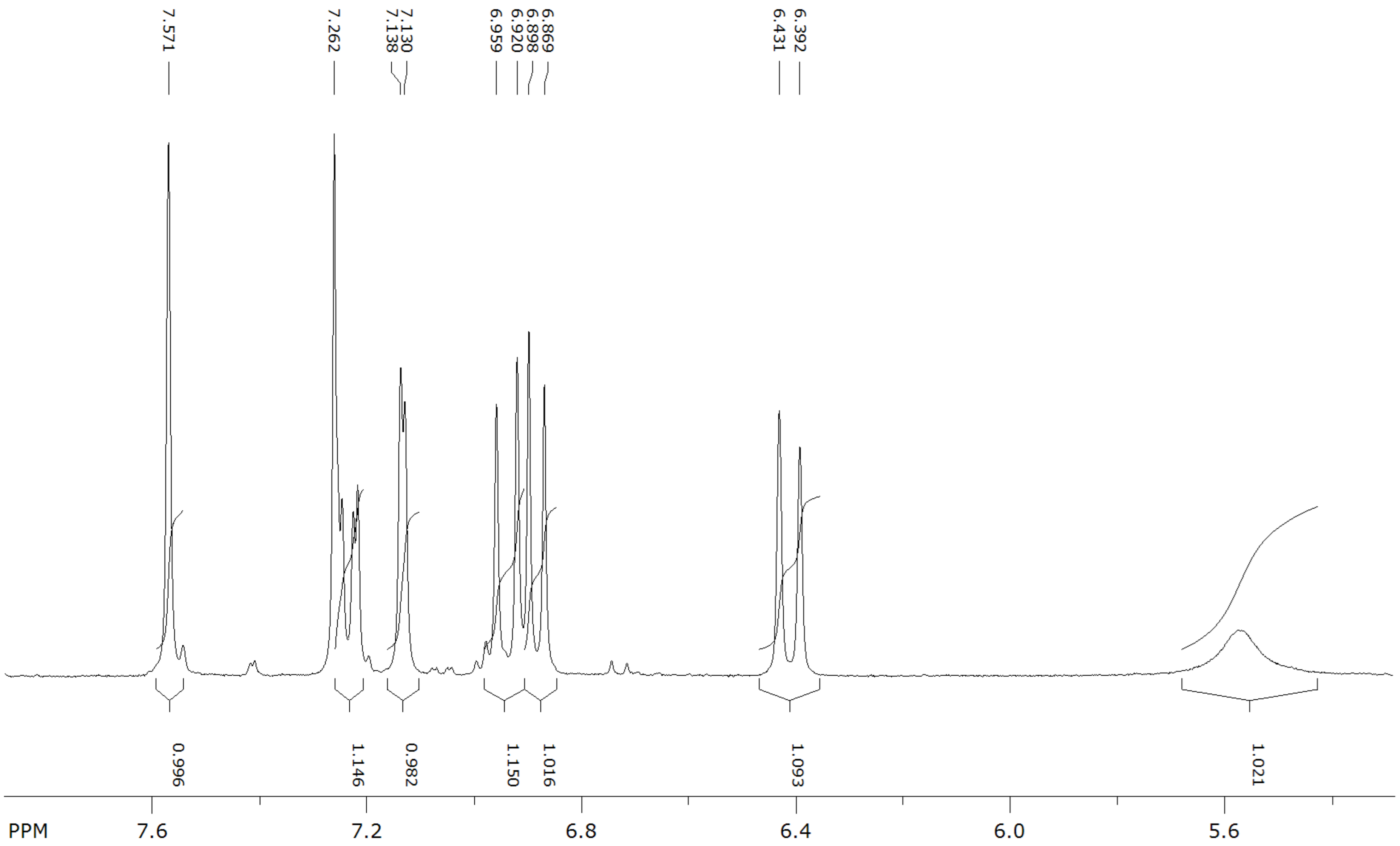


**Figure S45.** Aromatic part of $^{1}H$ NMR ($CDCl_3$) spectrum of **10**.

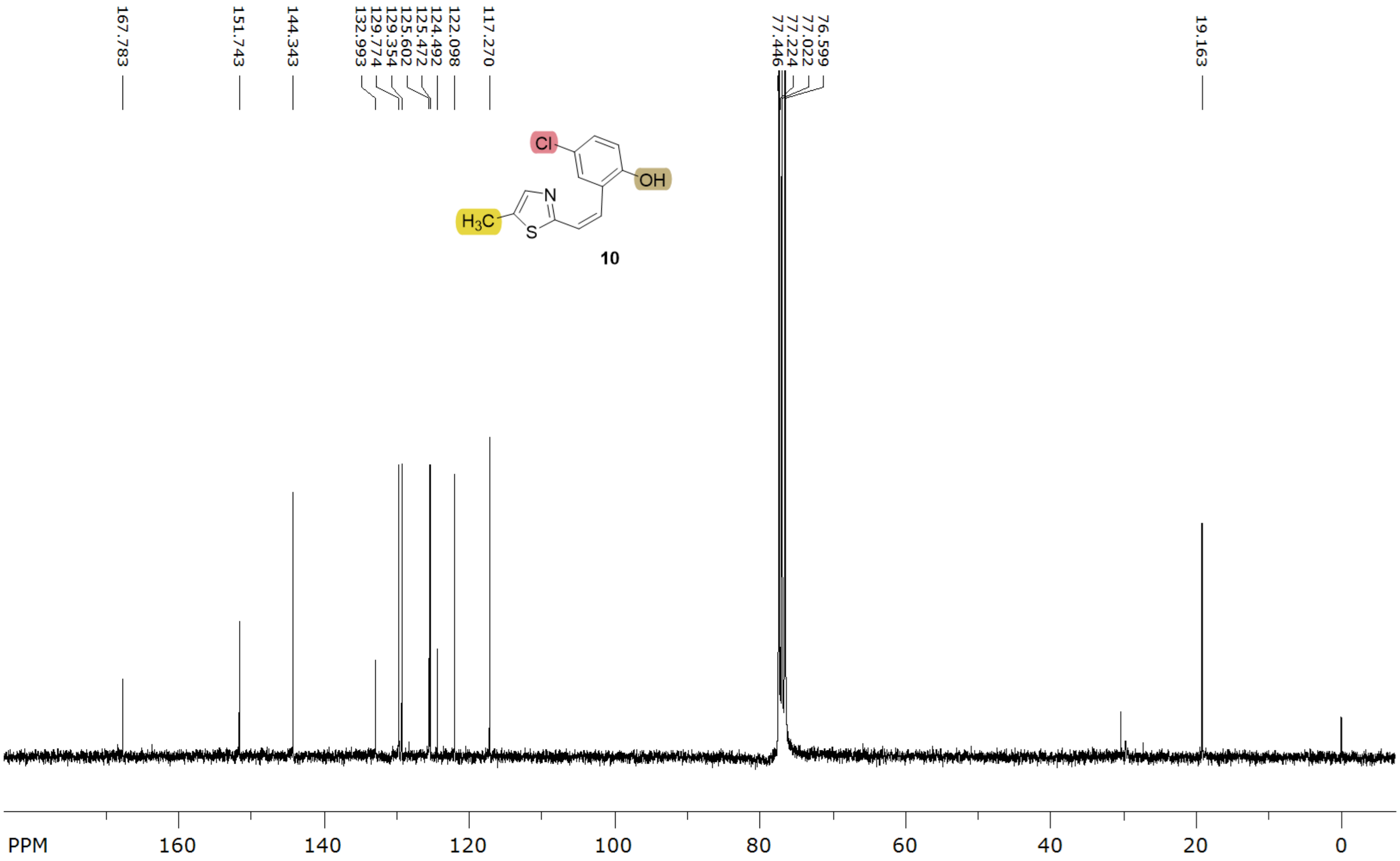


**Figure S46.** $^{13}C$ NMR ($CDCl_3$) spectrum of **10**.

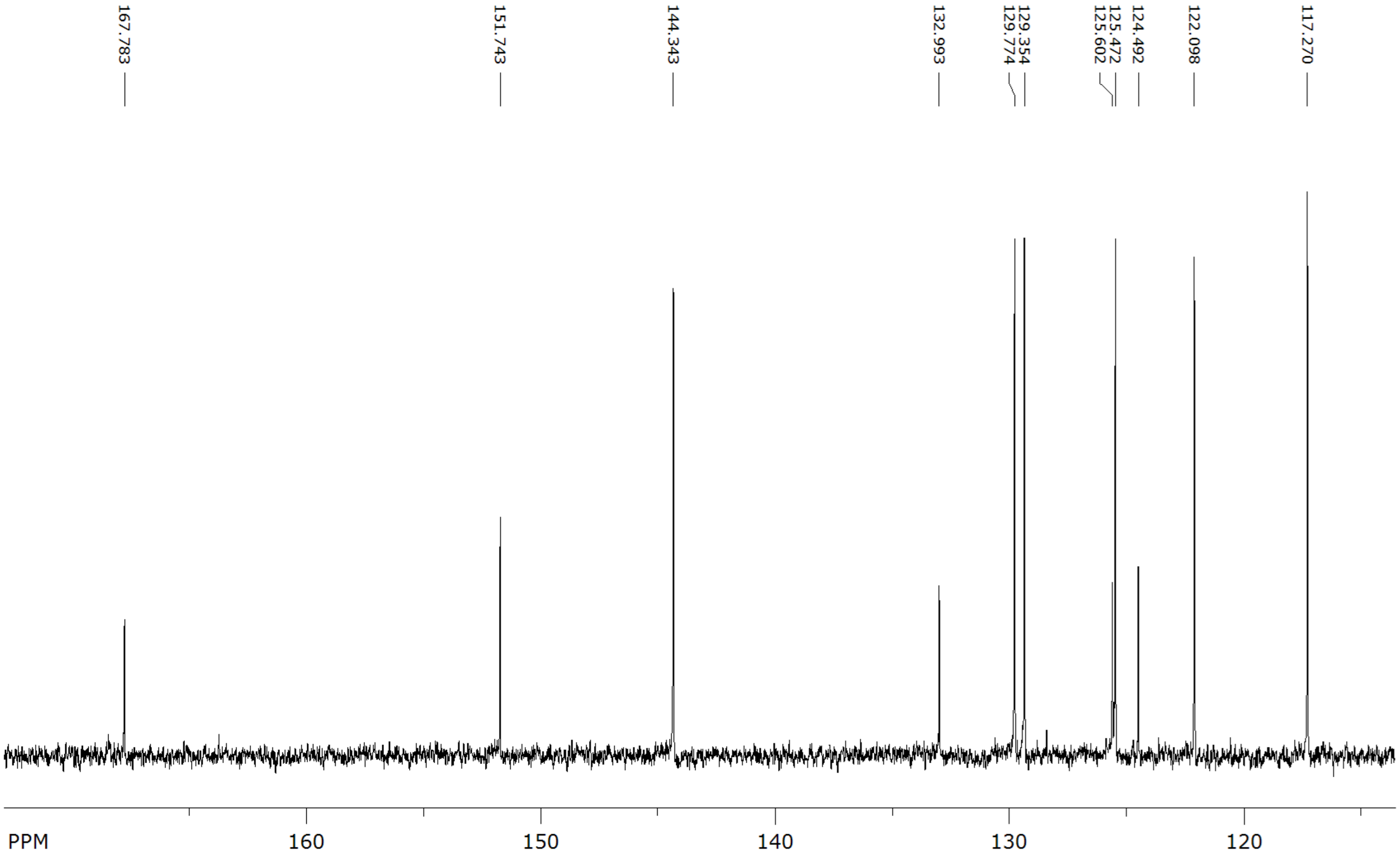


**Figure S47.** Aromatic part of $^{13}C$ NMR ($CDCl_3$) spectrum of **10**.

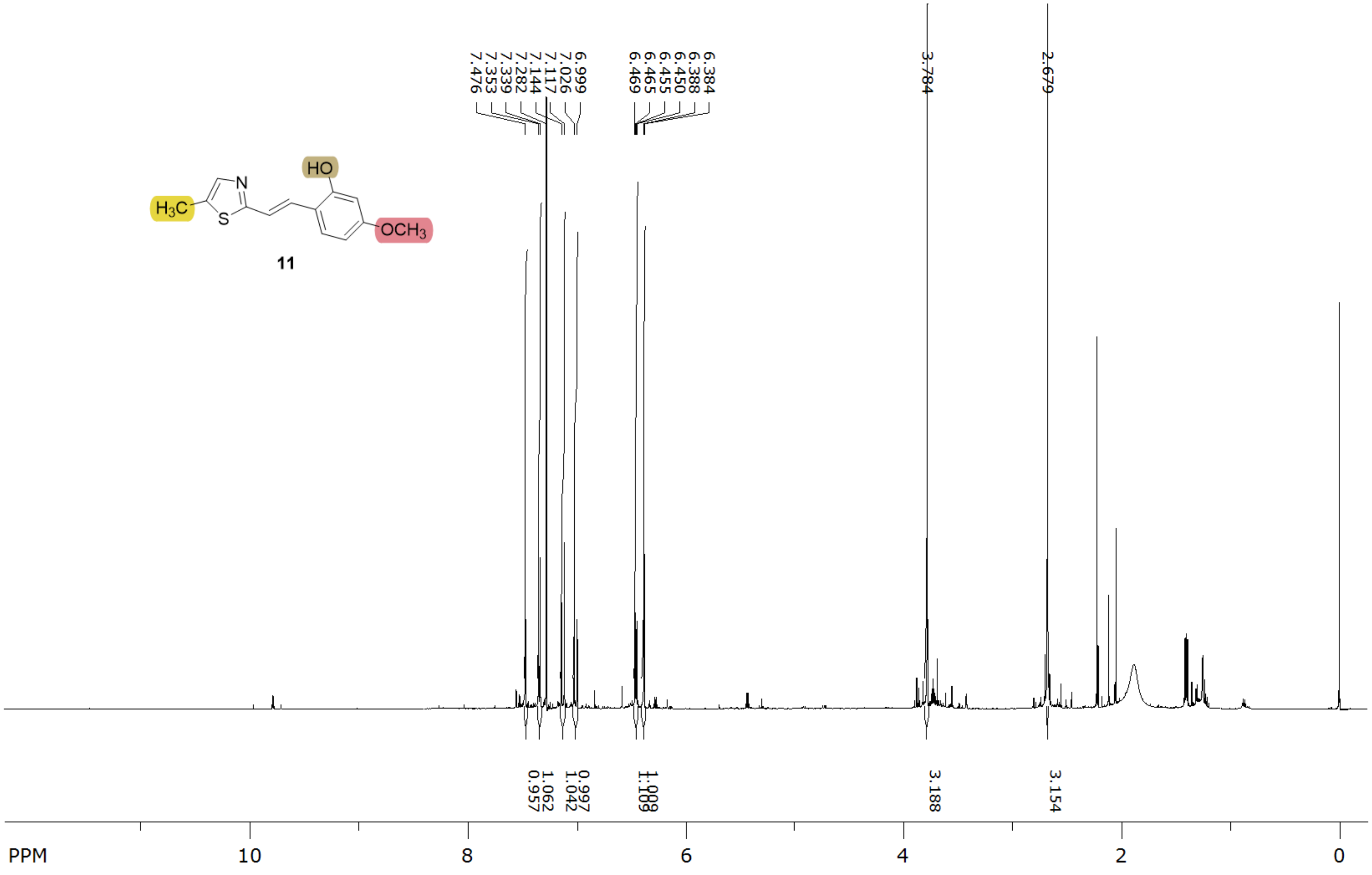


**Figure S48.** $^{1}$H NMR ($CDCl_3$) spectrum of **11**.

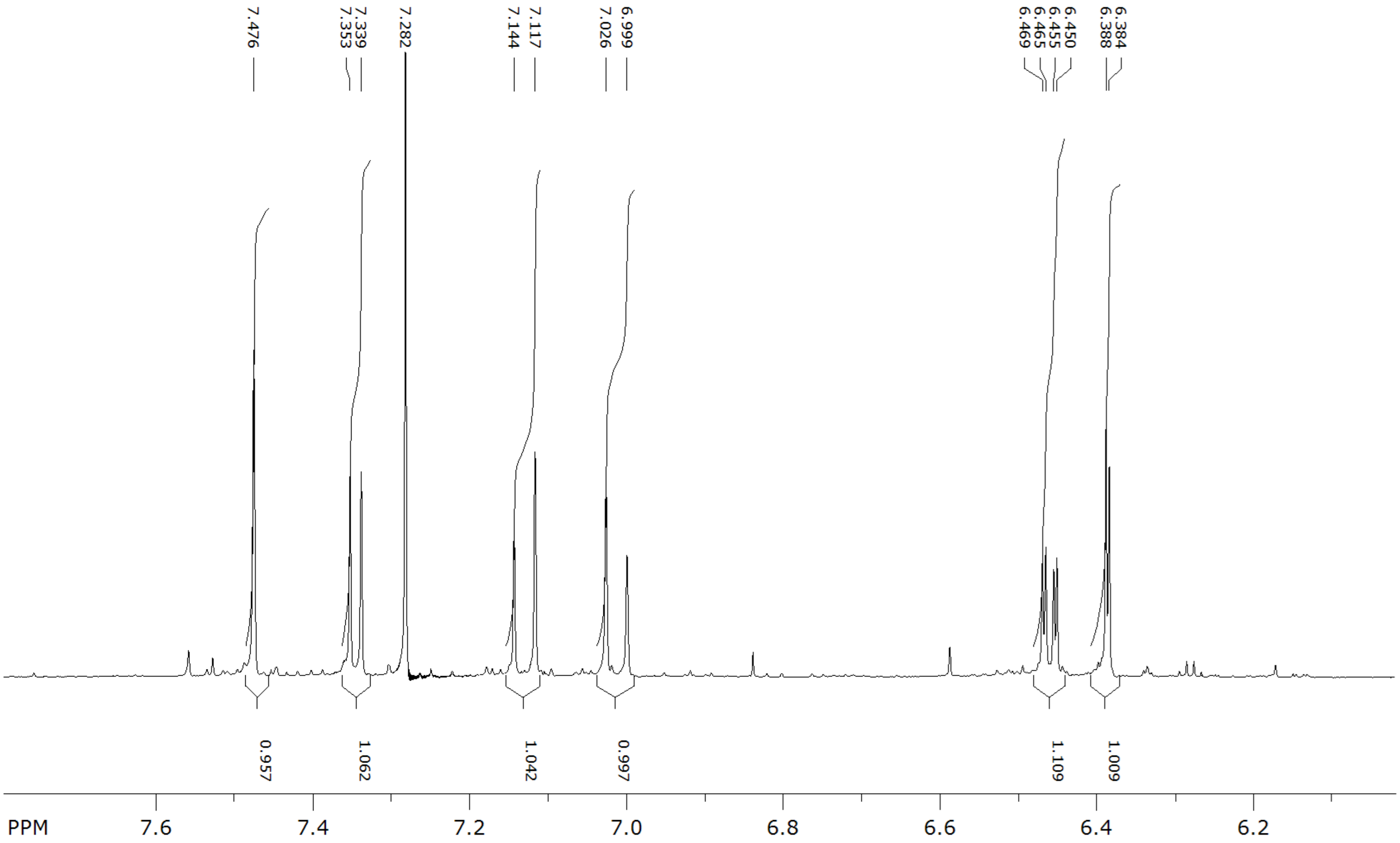


**Figure S49.** Aromatic part of $^{1}$H NMR ($CDCl_3$) spectrum of **11**.

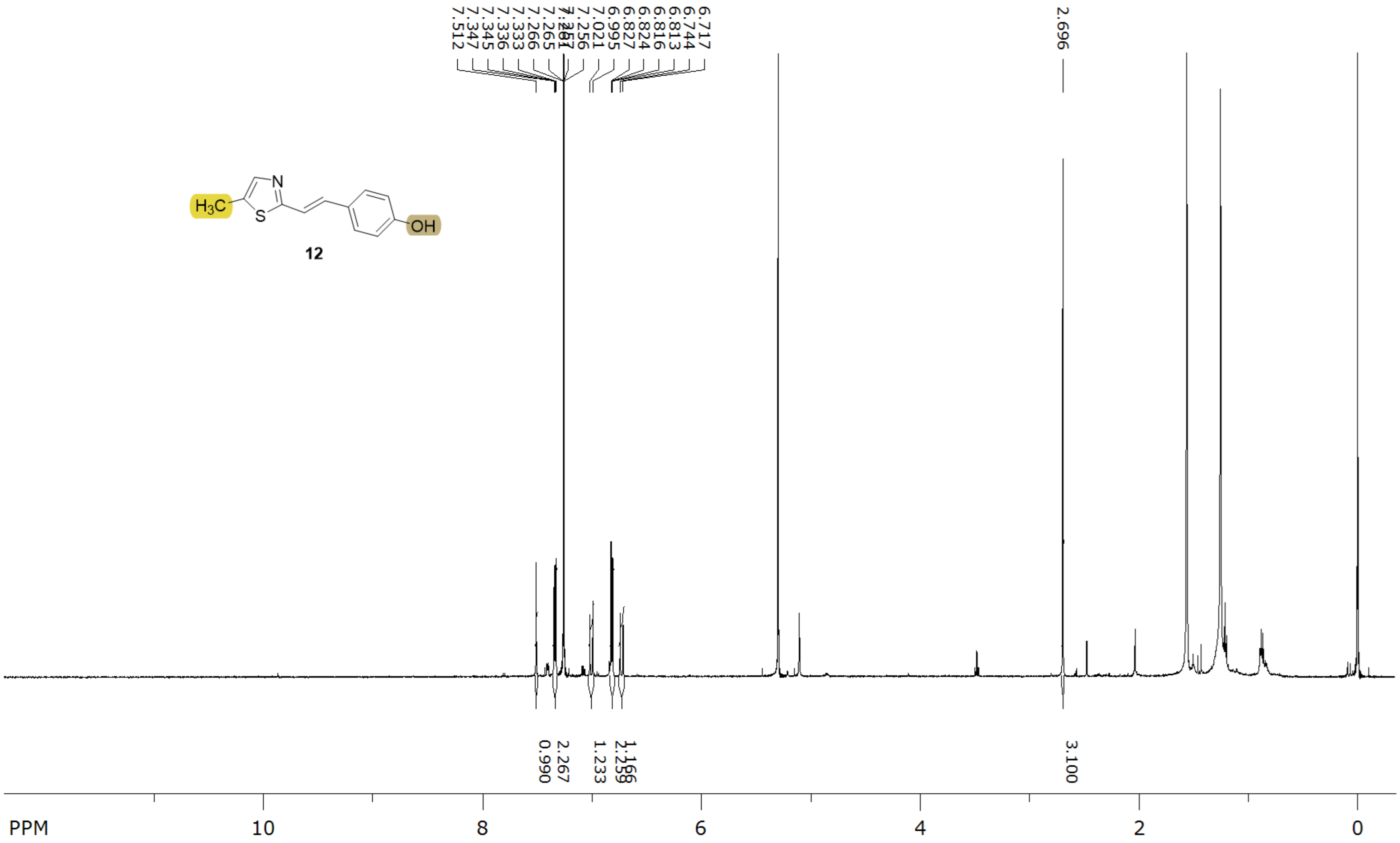


**Figure S50.** $^{1}$H NMR ($CDCl_3$) spectrum of **12**.

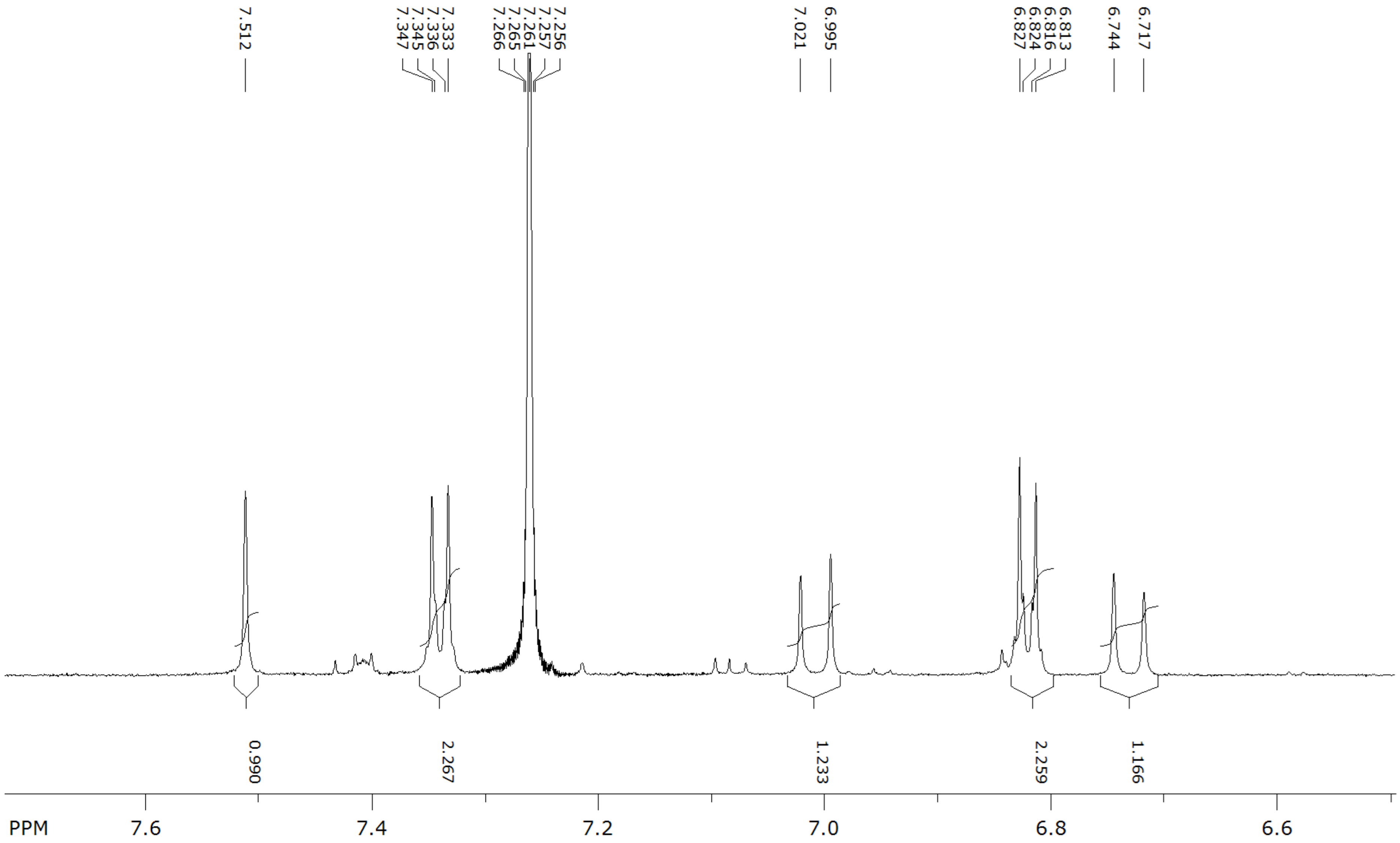


**Figure S51.** Aromatic part of $^{1}$H NMR ($CDCl_3$) spectrum of **12**.

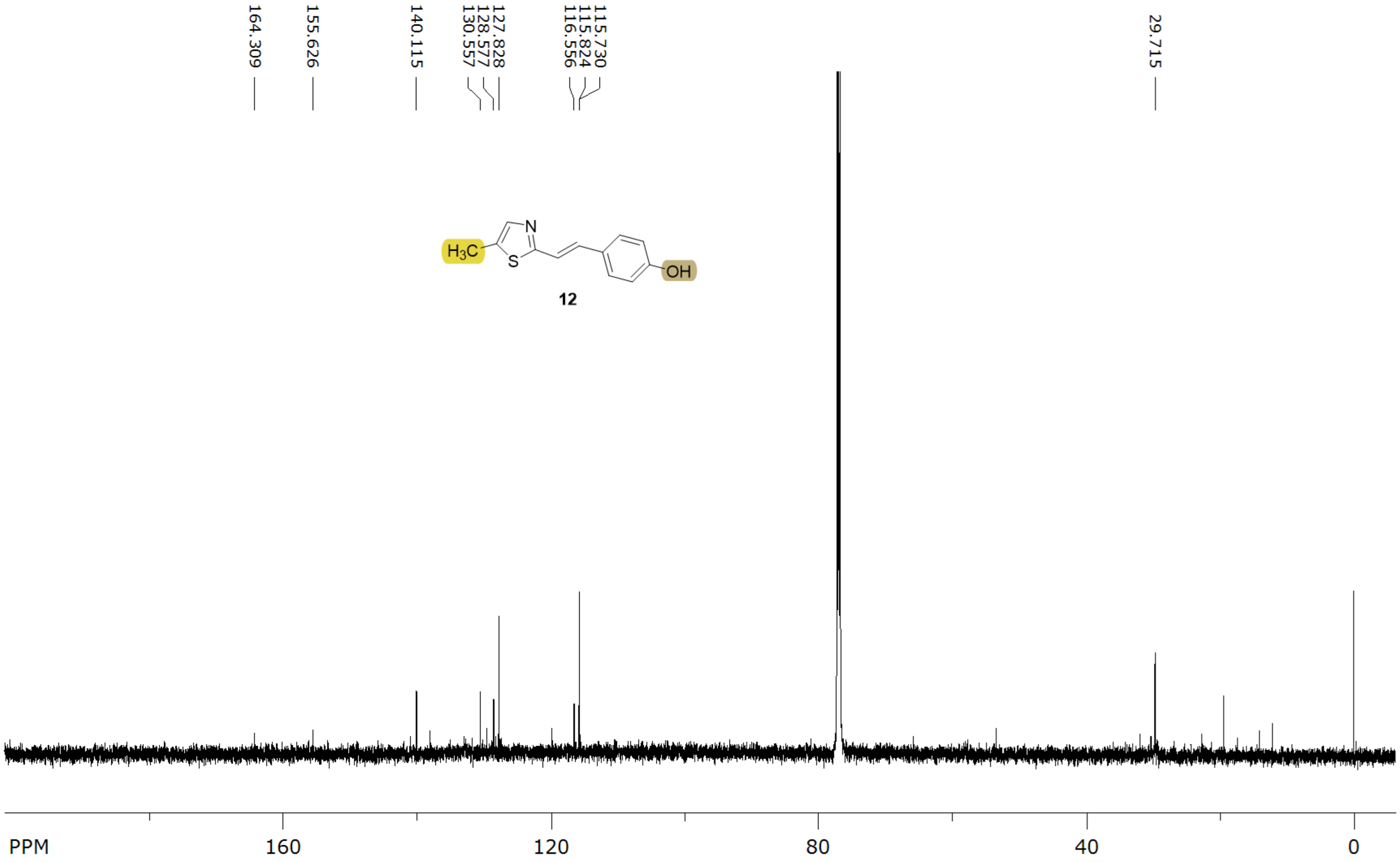


**Figure S52.** $^{13}C$ NMR ($CDCl_3$) spectrum of **12**.

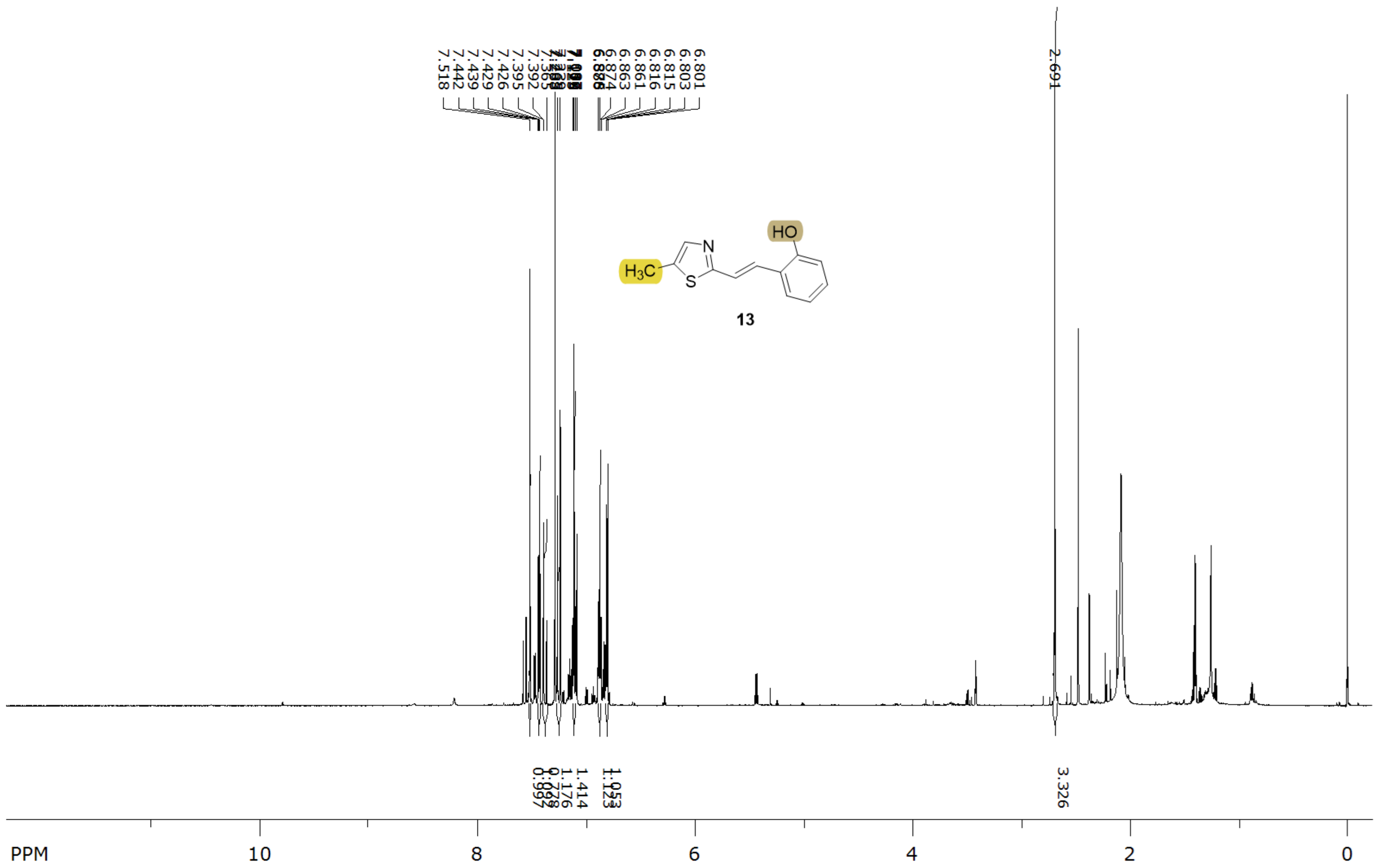


**Figure S53.** $^{1}$H NMR (CDCl3) spectrum of **13**.

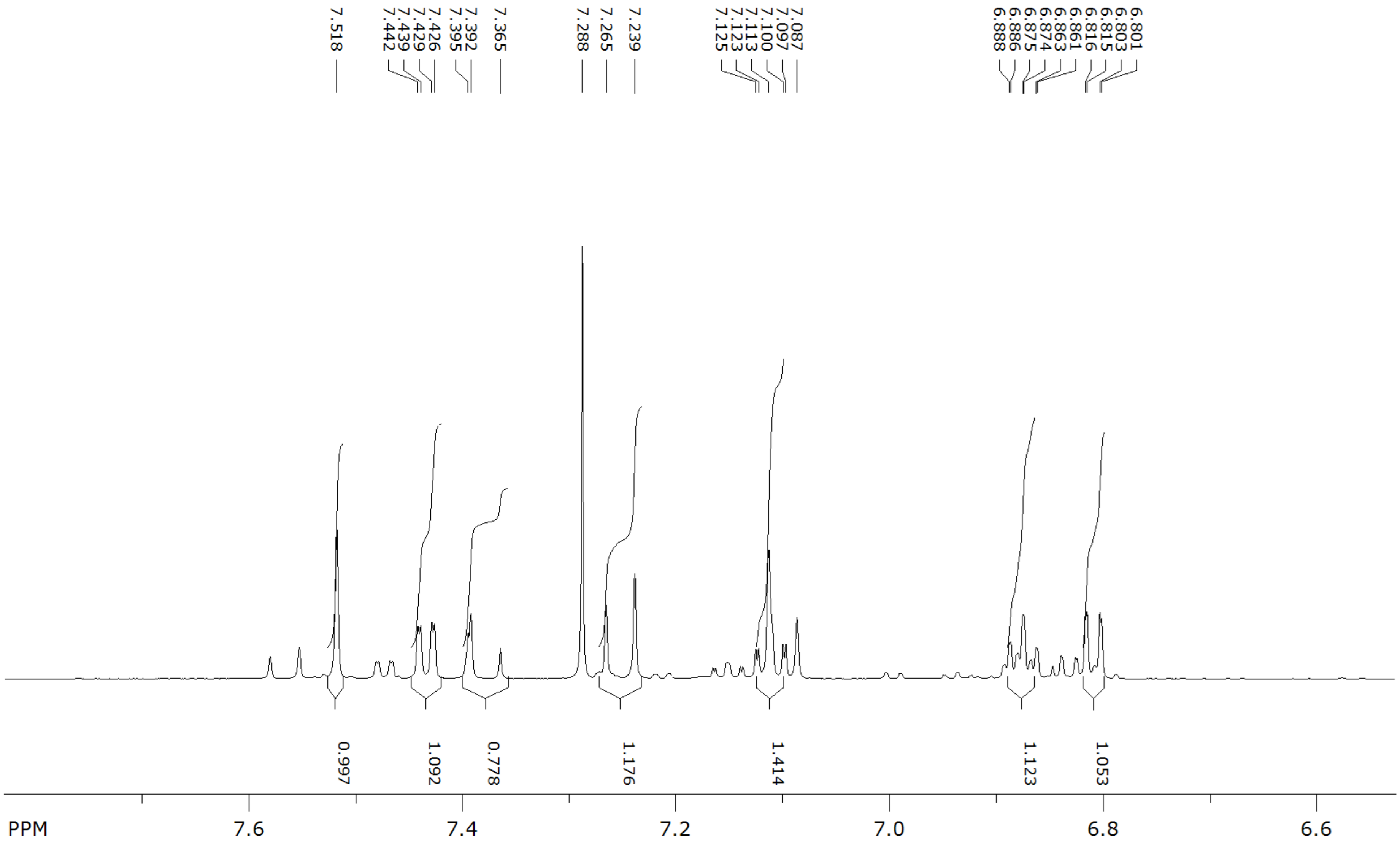


**Figure S54.** Aromatic part of $^{1}$H NMR ($CDCl_3$) spectrum of **13** (with a small amount of *cis*-isomer).

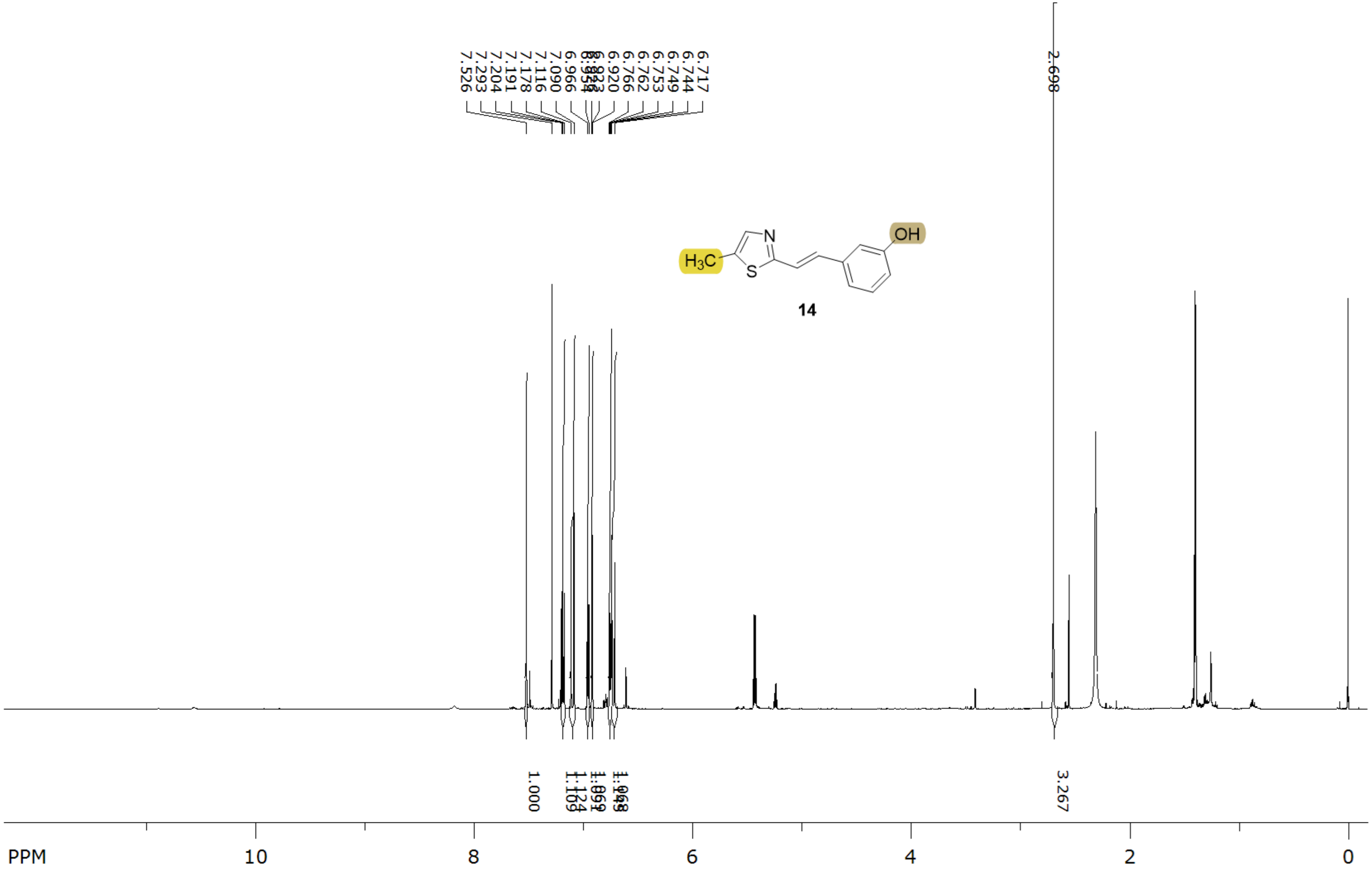


**Figure S55.** $^{1}$H NMR ($CDCl_3$) spectrum of **14**.

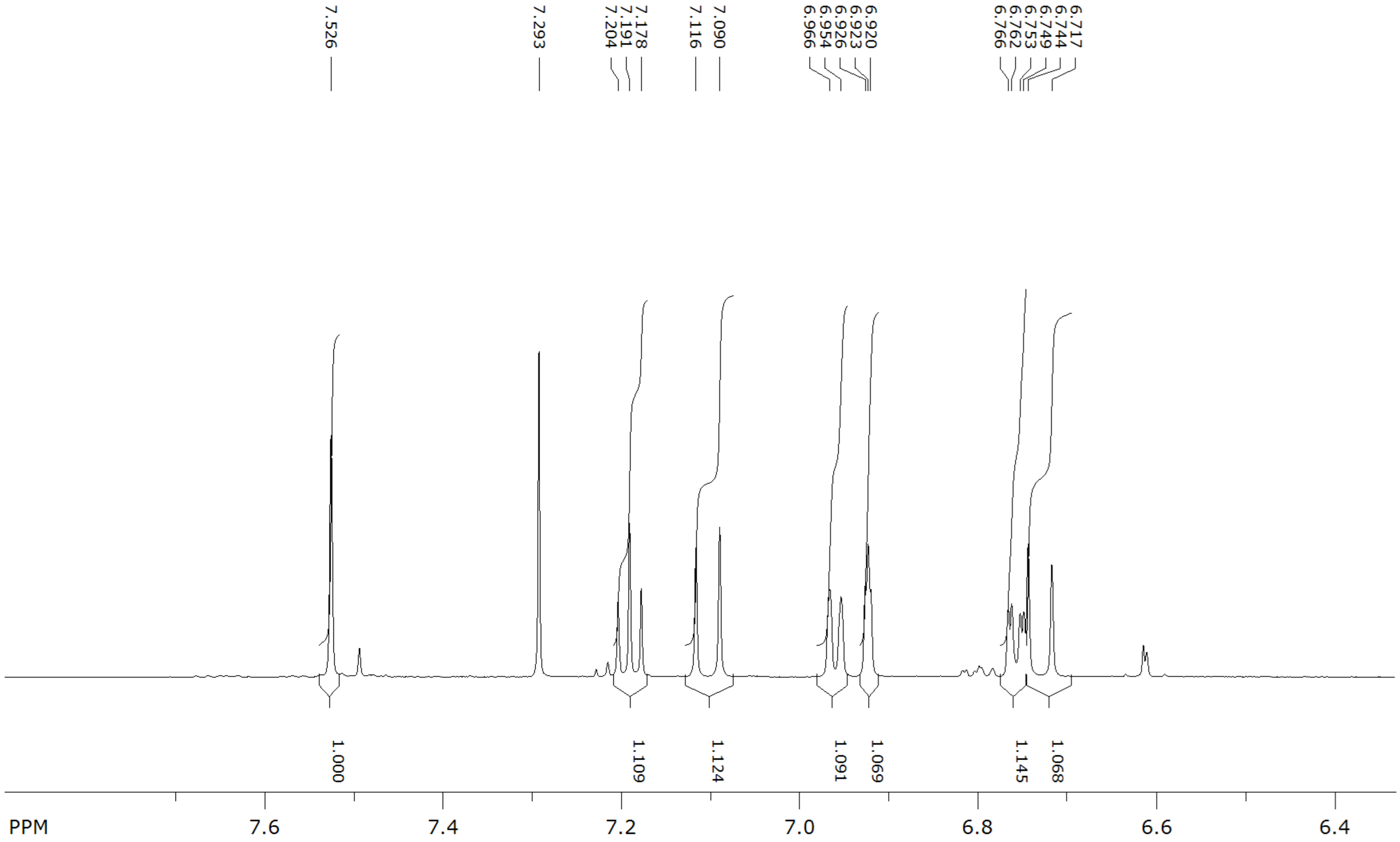


**Figure S56.** Aromatic part of $^1$H NMR ($CDCl_3$) spectrum of **14**.

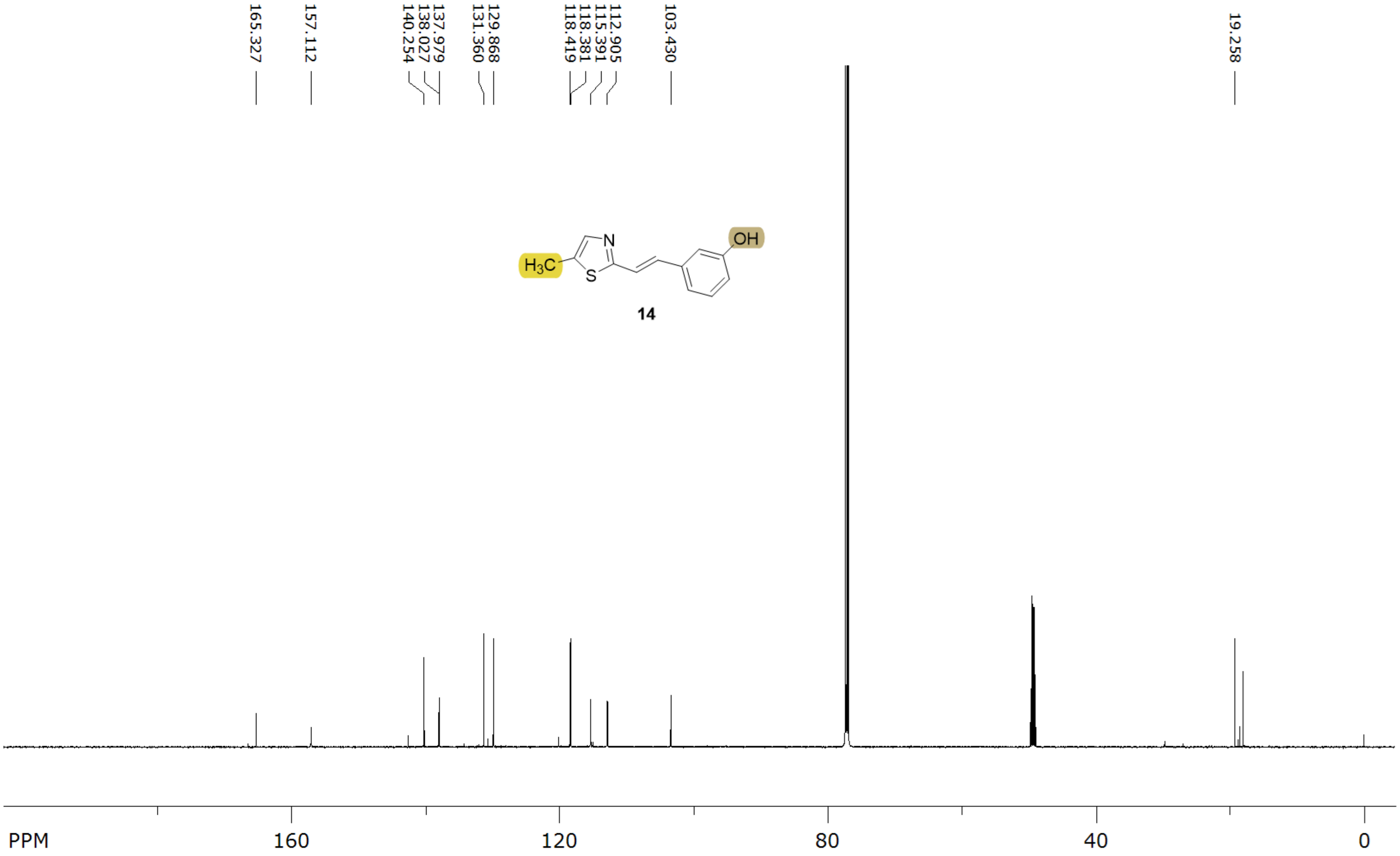


**Figure S57.** $^{13}$C NMR ($CDCl_3$) spectrum of **14**.

**2.** Mass spektra and HRMS analyses of compounds **1–14**

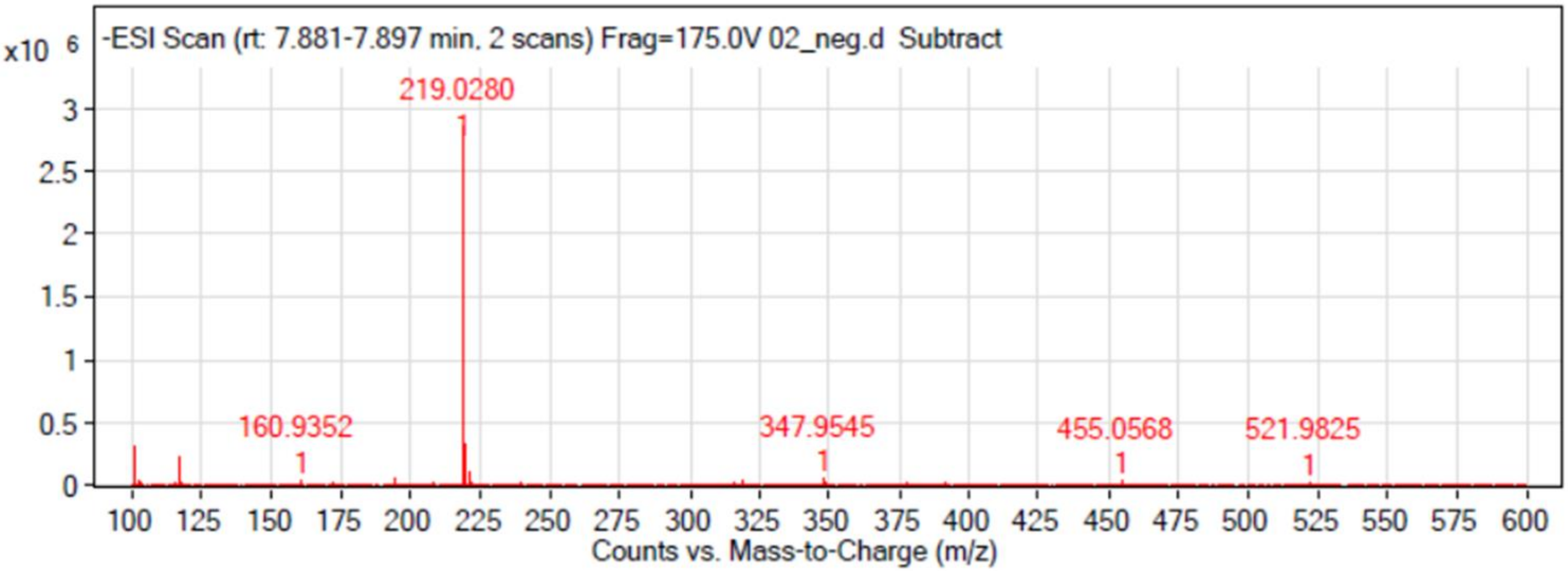


**Formula Calculator Results**

| Formula | Best | Mass | Tgt Mass | Diff (ppm) | Ion Species | Score |
|---|---|---|---|---|---|---|
| C12 H9 F O S | True | 220.0353 | 220.0358 | 2.47 | C12 H8 F O S | 96.28 |

**Figure S58.** Mass spectrum and HRMS analysis of compound **1**.

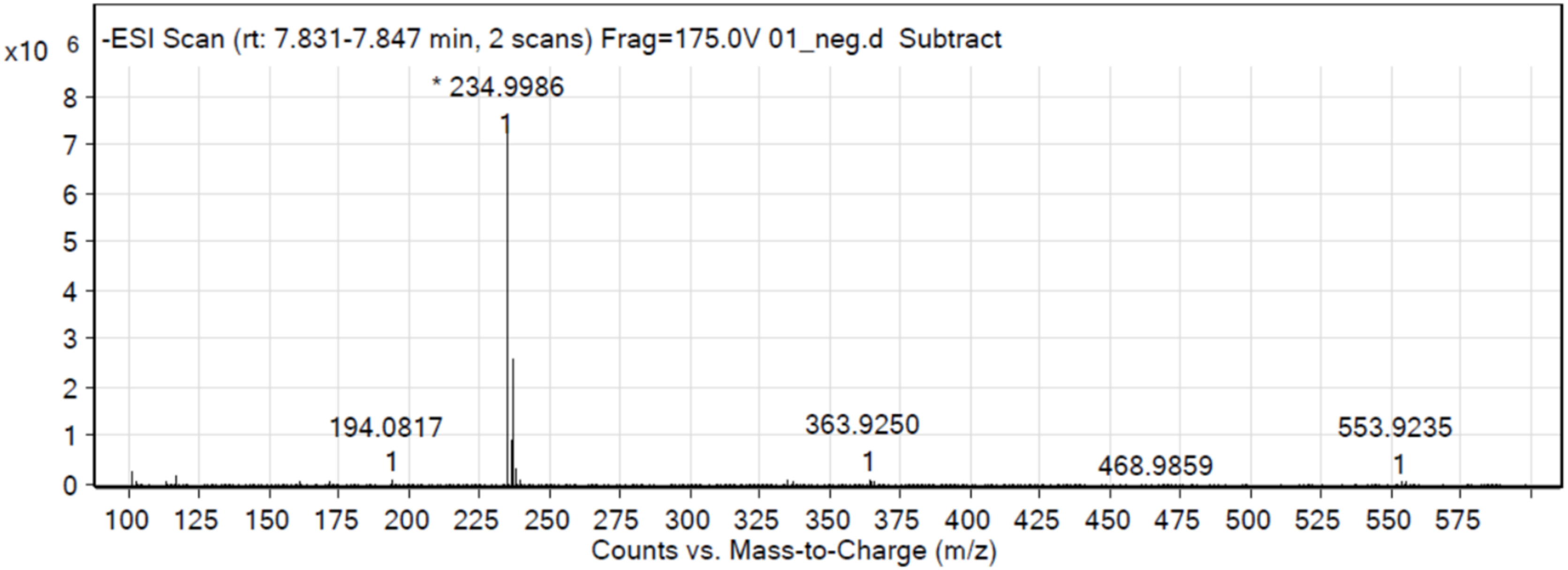


**Formula Calculator Results**

| Formula | Best | Mass | Tgt Mass | Diff (ppm) | Ion Species | Score |
|---|---|---|---|---|---|---|
| C12 H9 Cl O S | True | 236.0057 | 236.0063 | 2.24 | C12 H8 Cl O S | 97.44 |

**Figure S59.** Mass spectrum and HRMS analysis of compound **2**.

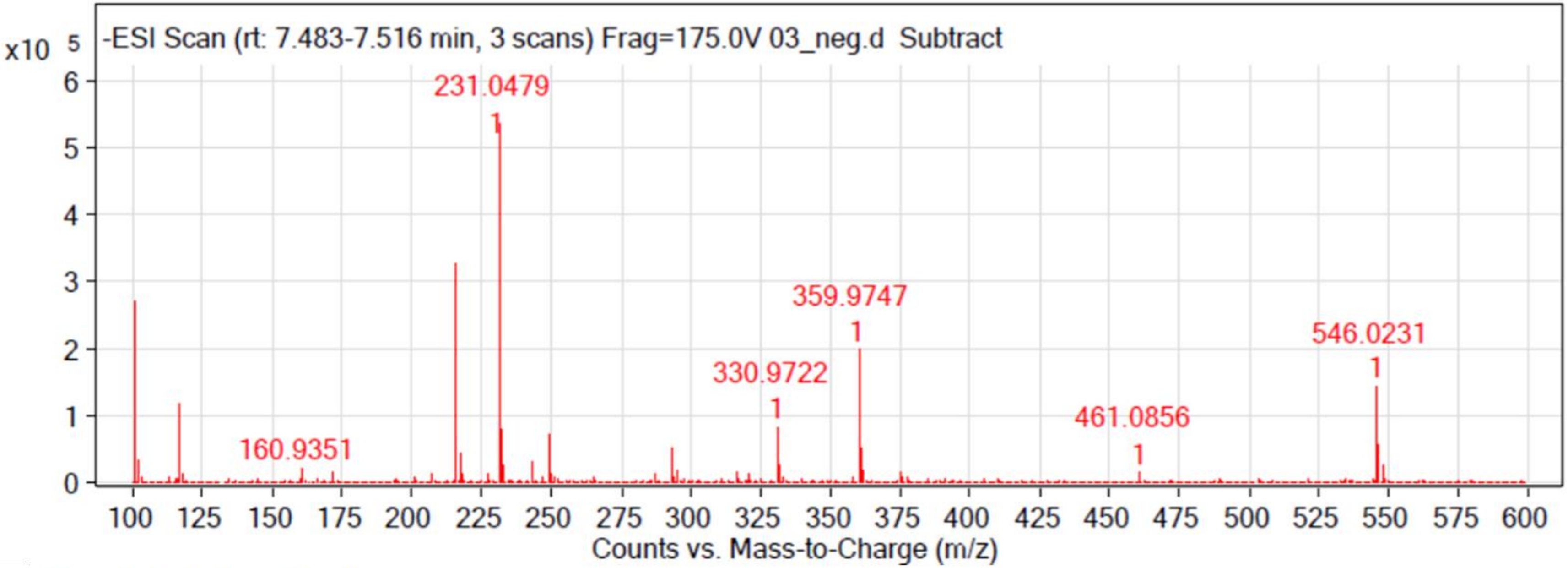


**Formula Calculator Results**

| Formula | Best | Mass | Tgt Mass | Diff (ppm) | Ion Species | Score |
|---|---|---|---|---|---|---|
| C13 H12 O2 S | True | 232.0551 | 232.0558 | 2.85 | C13 H11 O2 S | 97.16 |

**Figure S60.** Mass spectrum and HRMS analysis of compound **3**.

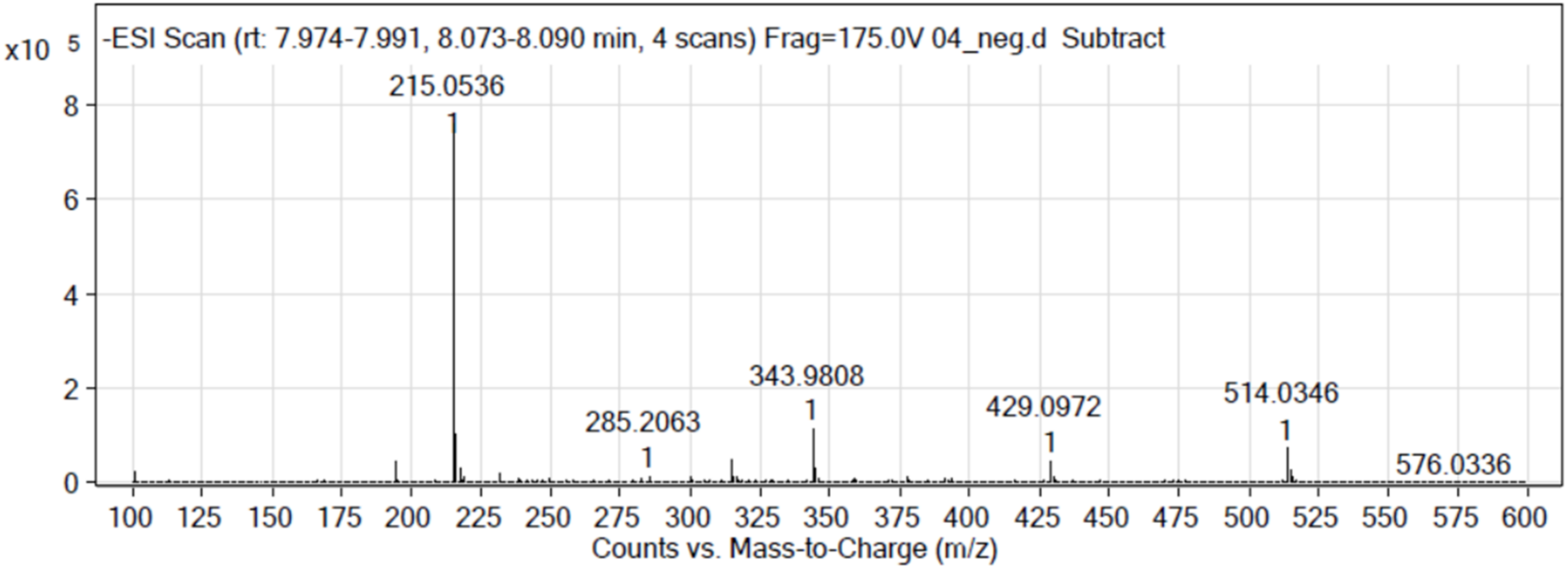


**Formula Calculator Results**

| Formula | Best | Mass | Tgt Mass | Diff (ppm) | Ion Species | Score |
|---|---|---|---|---|---|---|
| C13 H12 O S | True | 216.0609 | 216.0609 | 0.16 | C13 H11 O S | 98.74 |

**Figure S61.** Mass spectrum and HRMS analysis of compound **4**.

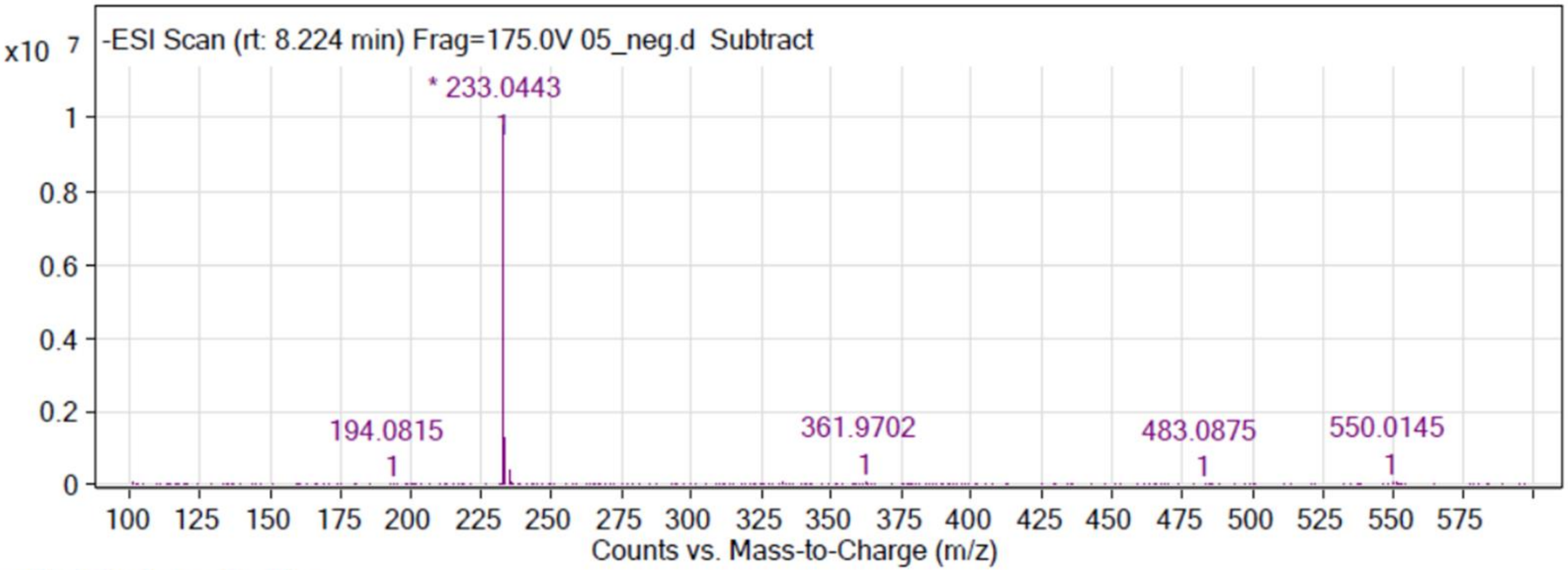


**Formula Calculator Results**

| Formula | Best | Mass | Tgt Mass | Diff (ppm) | Ion Species | Score |
|---|---|---|---|---|---|---|
| C13 H11 F O S | True | 234.0515 | 234.0515 | 0.06 | C13 H10 F O S | 98.06 |

**Figure S62.** Mass spectrum and HRMS analysis of compound **5**.

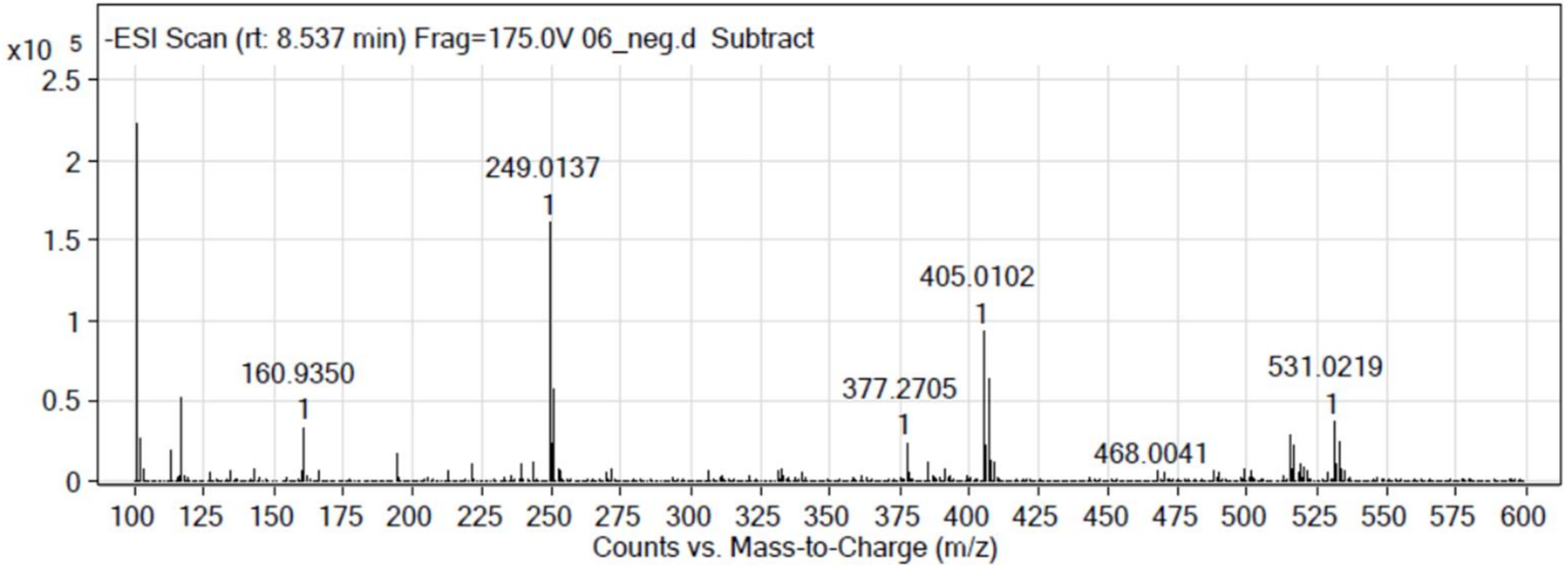


**Formula Calculator Results**

| Formula | Best | Mass | Tgt Mass | Diff (ppm) | Ion Species | Score |
|---|---|---|---|---|---|---|
| C13 H11 Cl O S | True | 250.0216 | 250.0219 | 1.14 | C13 H10 Cl O S | 94.7 |

**Figure S63.** Mass spectrum and HRMS analysis of compound **6**.

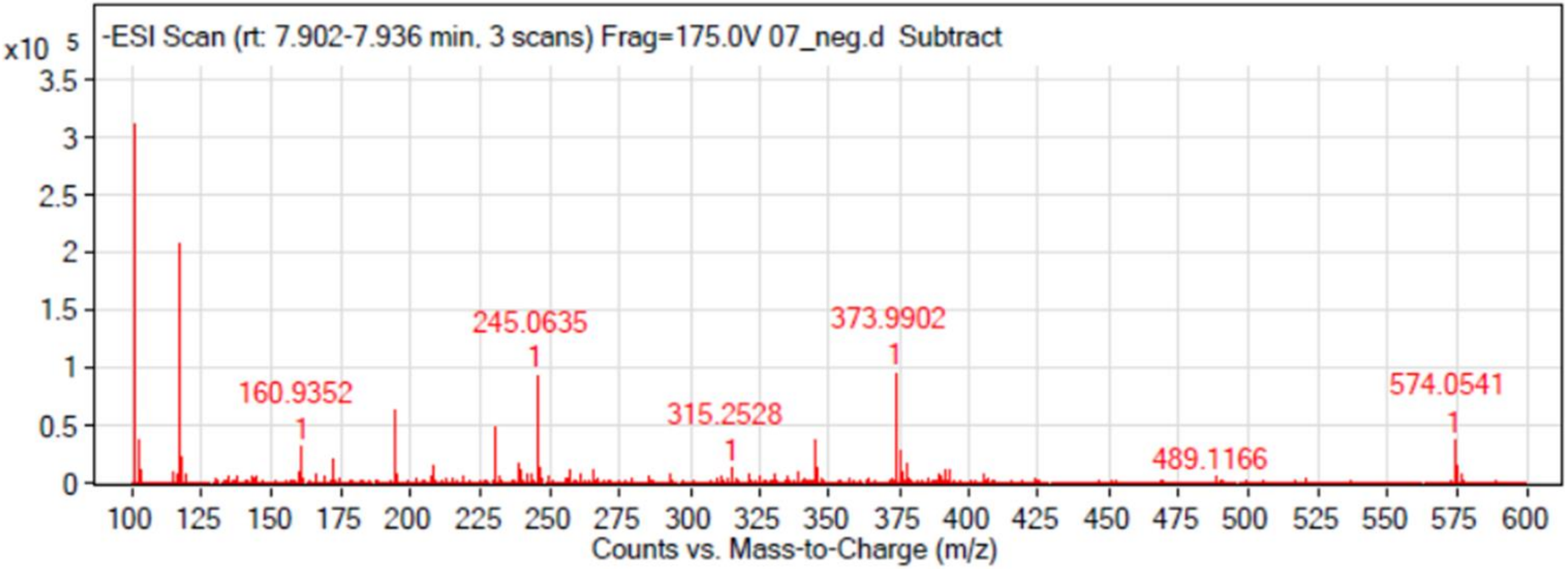


**Formula Calculator Results**

| Formula | Best | Mass | Tgt Mass | Diff (ppm) | Ion Species | Score |
|---|---|---|---|---|---|---|
| C14 H14 O2 S | True | 246.0708 | 246.0715 | 2.68 | C14 H13 O2 S | 96.34 |

**Figure S64.** Mass spectrum and HRMS analysis of compound **7**.

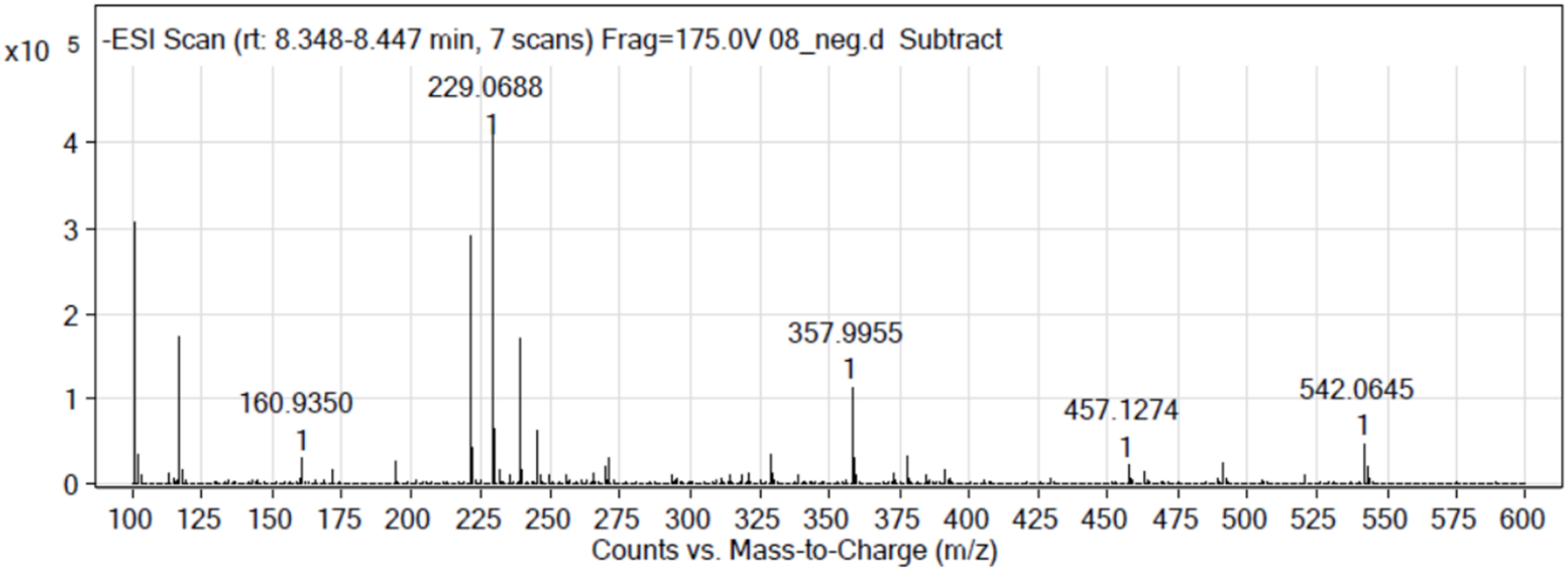


**Formula Calculator Results**

| Formula | Best | Mass | Tgt Mass | Diff (ppm) | Ion Species | Score |
|---|---|---|---|---|---|---|
| C14 H14 O S | True | 230.076 | 230.0765 | 2.36 | C14 H13 O S | 97.4 |

**Figure S65.** Mass spectrum and HRMS analysis of compound **8**.

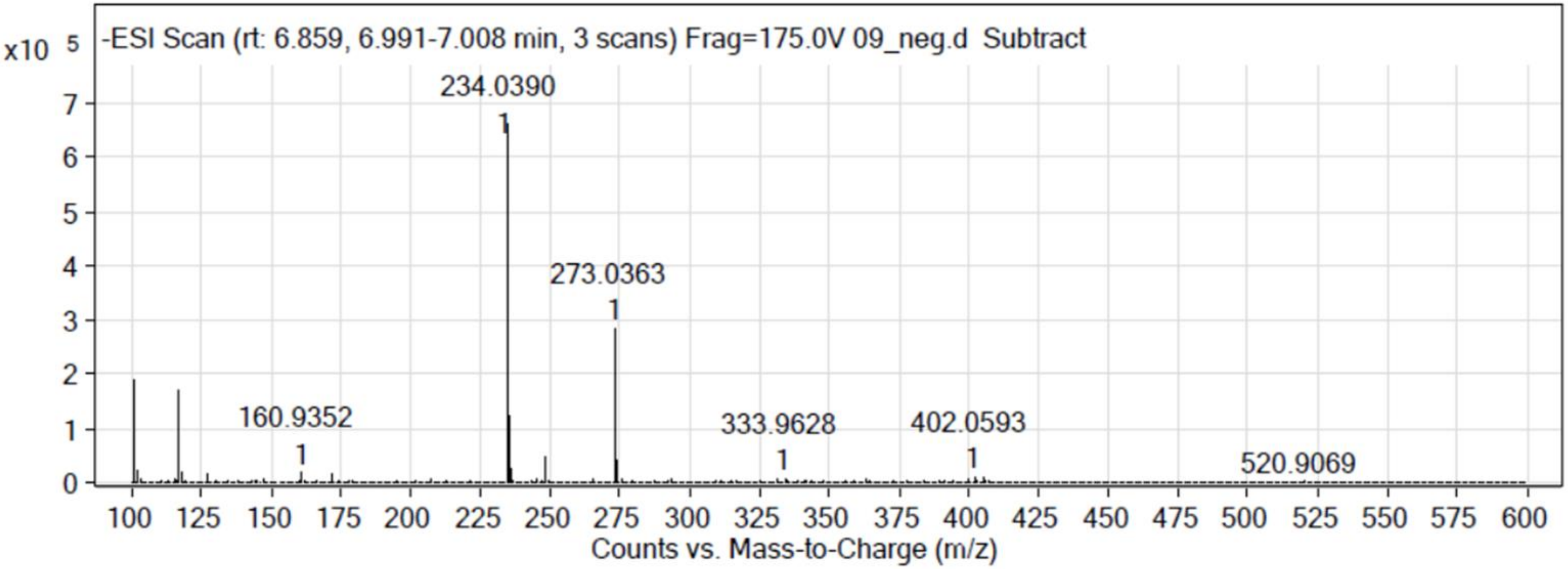


**Formula Calculator Results**

| Formula | Best | Mass | Tgt Mass | Diff (ppm) | Ion Species | Score |
|---|---|---|---|---|---|---|
| C12 H10 F N O S | True | 235.0469 | 235.0467 | -0.93 | C12 H9 F N O S | 84.55 |

**Figure S66.** Mass spectrum and HRMS analysis of compound **9**.

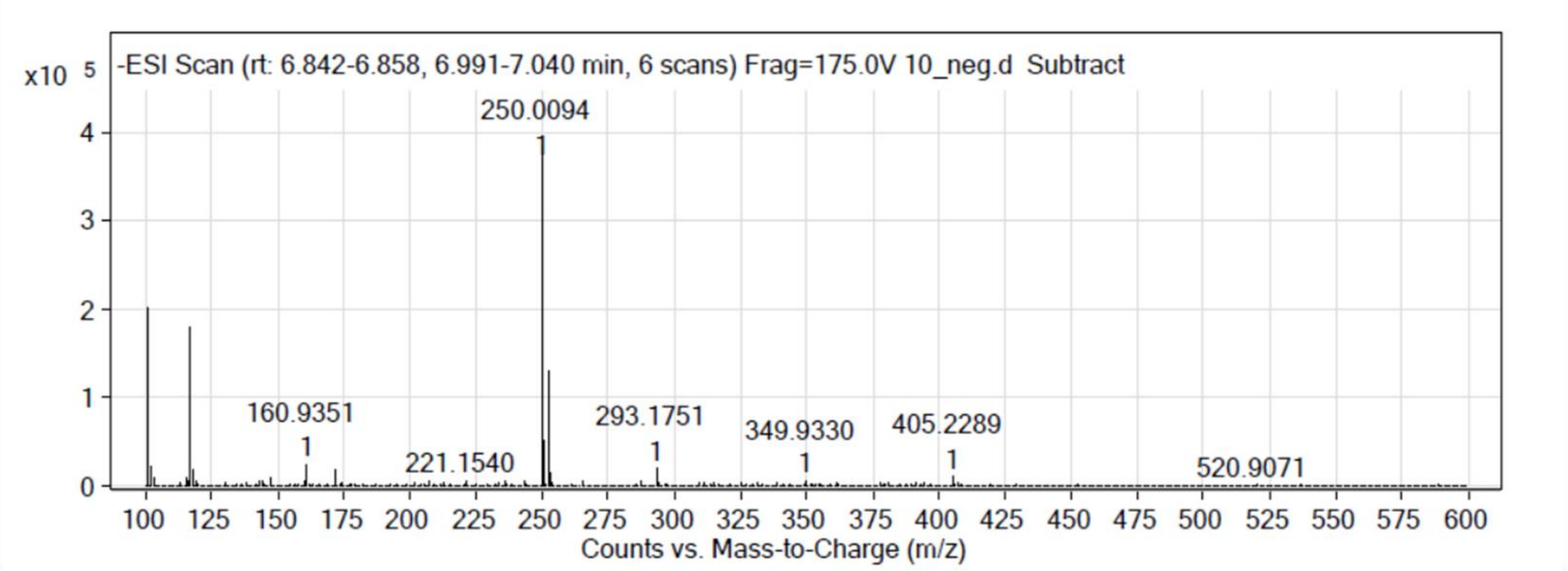


**Formula Calculator Results**

| Formula | Best | Mass | Tgt Mass | Diff (ppm) | Ion Species | Score |
|---|---|---|---|---|---|---|
| C12 H10 Cl N O S | True | 251.0165 | 251.0172 | 2.62 | C12 H9 Cl N O S | 96.98 |

**Figure S67.** Mass spectrum and HRMS analysis of compound **10**.

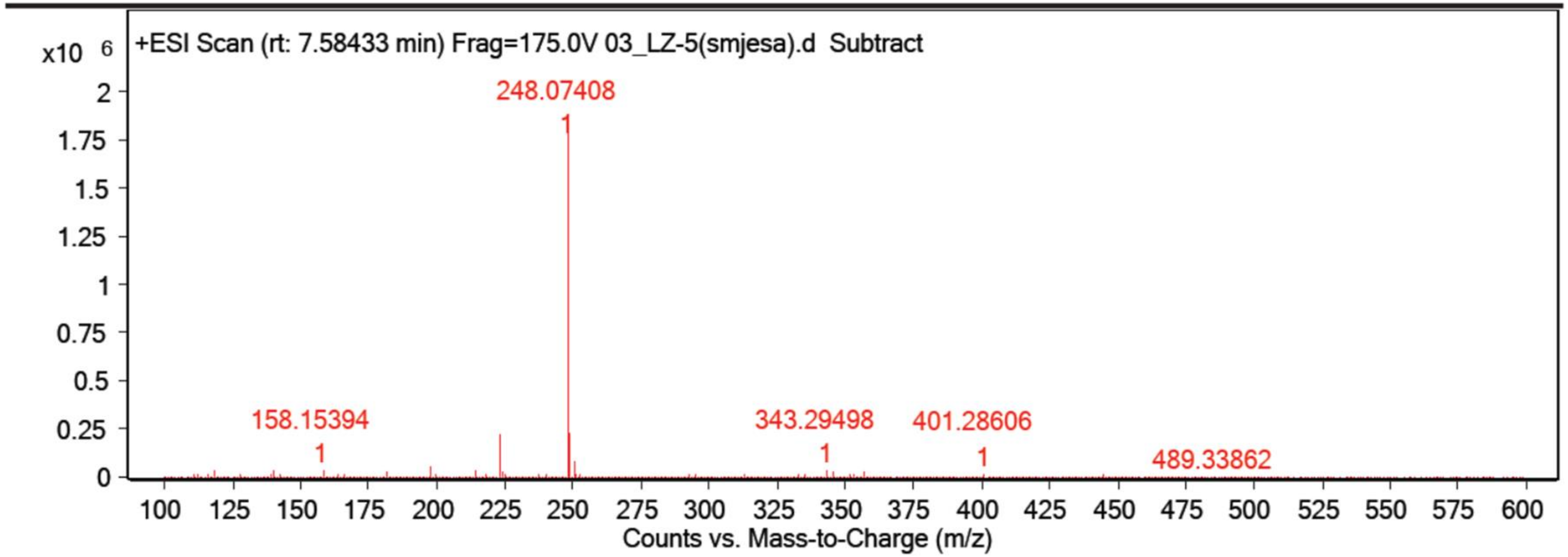


**Formula Calculator Results**

| Formula | Best | Mass | Tgt Mass | Diff (ppm) | Ion Species | Score |
|---|---|---|---|---|---|---|
| C13 H13 N O2 S | True | 247.06685 | 247.0667 | -0.6 | C13 H14 N O2 S | 96.67 |

**Figure S68.** Mass spectrum and HRMS analysis of compound **11**.

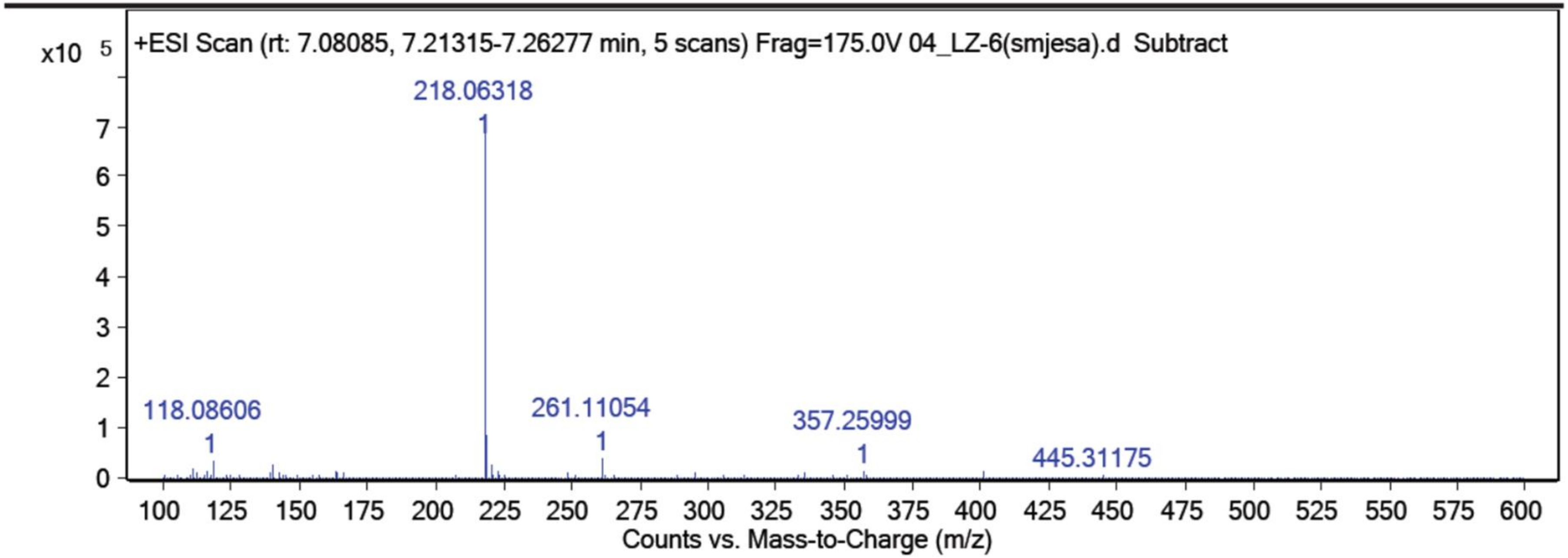


**Formula Calculator Results**

| Formula | Best | Mass | Tgt Mass | Diff (ppm) | Ion Species | Score |
|---|---|---|---|---|---|---|
| C12 H11 N O S | True | 217.05589 | 217.05613 | 1.14 | C12 H12 N O S | 97.45 |

**Figure S69.** Mass spectrum and HRMS analysis of compound **12**.

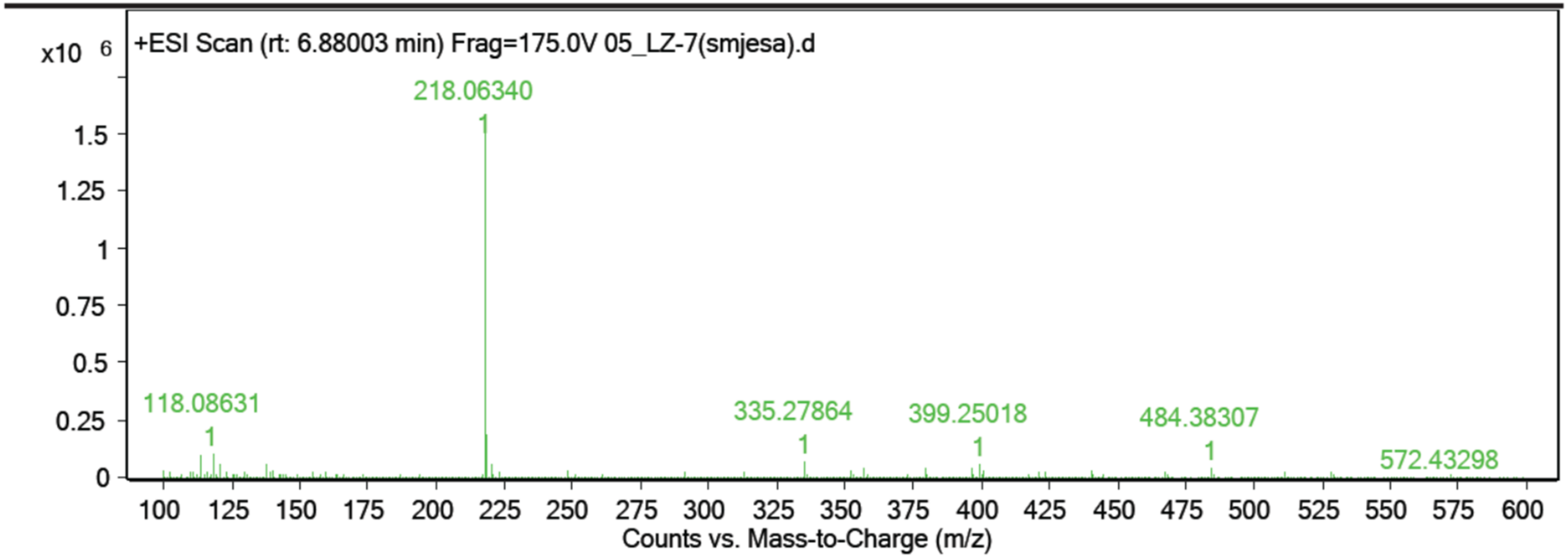


**Formula Calculator Results**

| Formula | Best | Mass | Tgt Mass | Diff (ppm) | Ion Species | Score |
|---|---|---|---|---|---|---|
| C12 H11 N O S | True | 217.05615 | 217.05613 | -0.06 | C12 H12 N O S | 97.57 |

**Figure S70.** Mass spectrum and HRMS analysis of compound **13**.

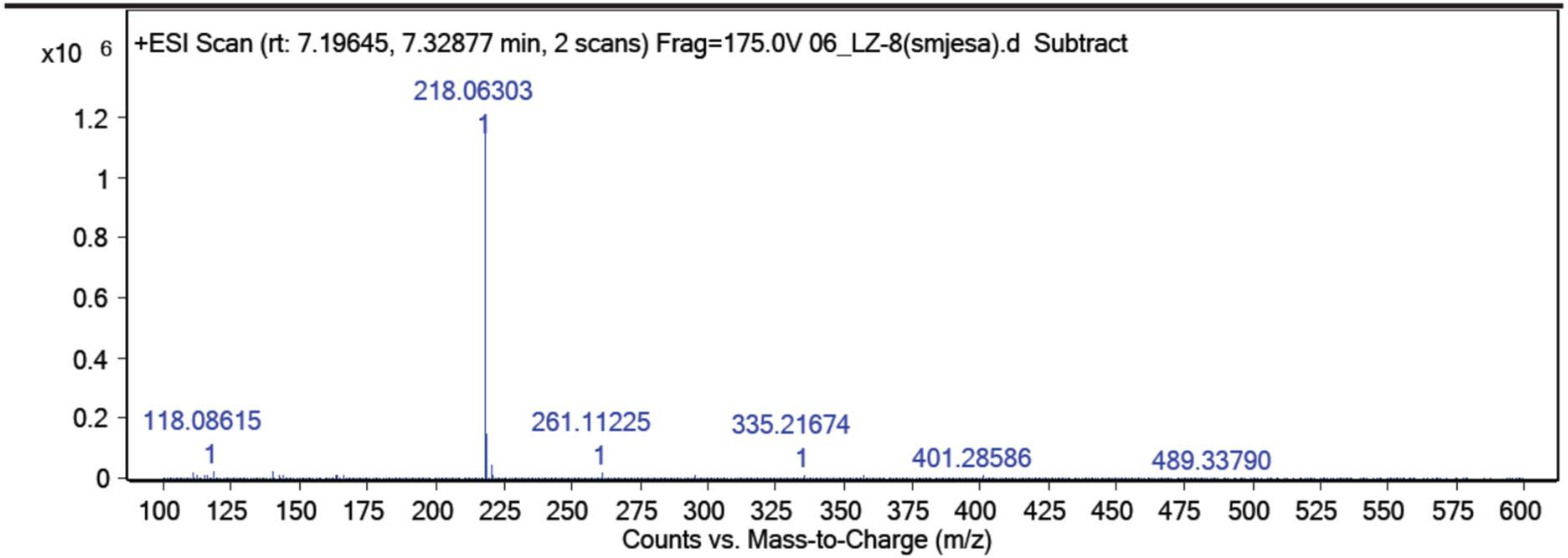


**Formula Calculator Results**

| Formula | Best | Mass | Tgt Mass | Diff (ppm) | Ion Species | Score |
|---|---|---|---|---|---|---|
| C12 H11 N O S | True | 217.05578 | 217.05613 | 1.64 | C12 H12 N O S | 97.44 |

**Figure S71.** Mass spectrum and HRMS analysis of compound **14**.

**3.** UV spectra of compounds

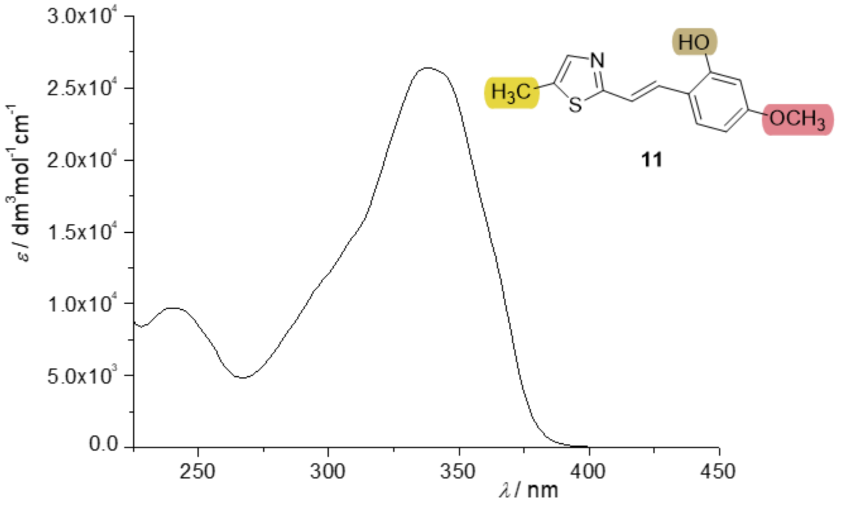


**Figure S72.** UV (ACN) spectrum of **11**.

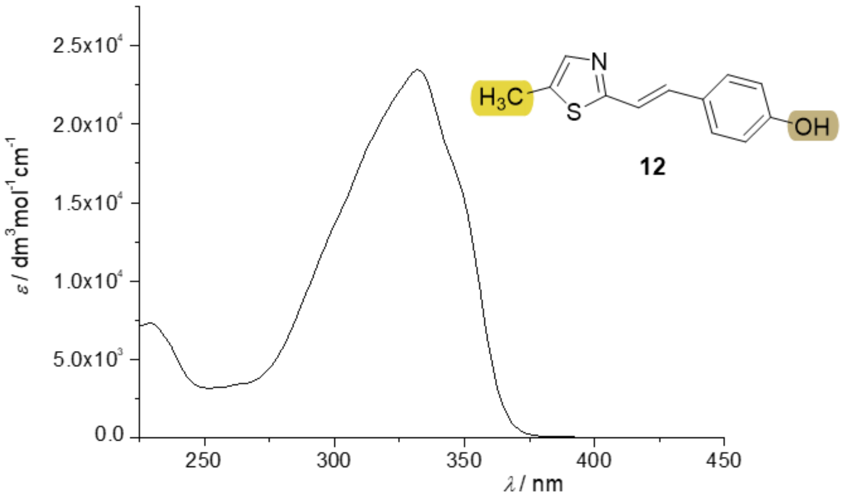


**Figure S73.** UV (ACN) spectrum of **12**.

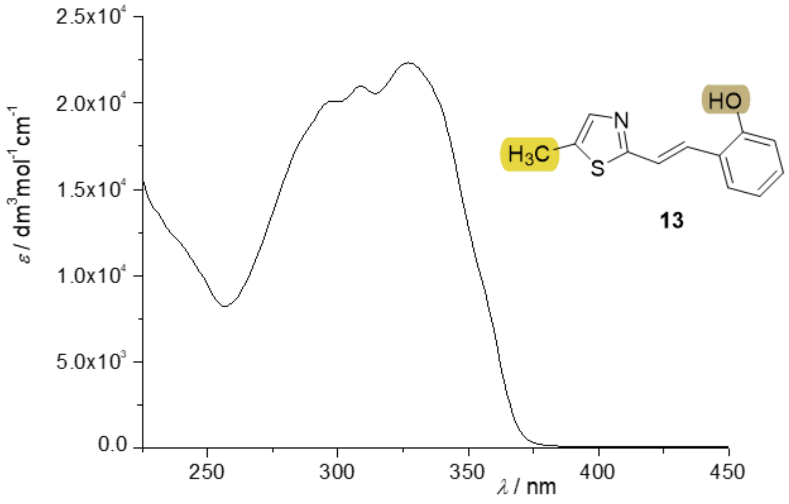


**Figure S74.** UV (ACN) spectrum of **13**.

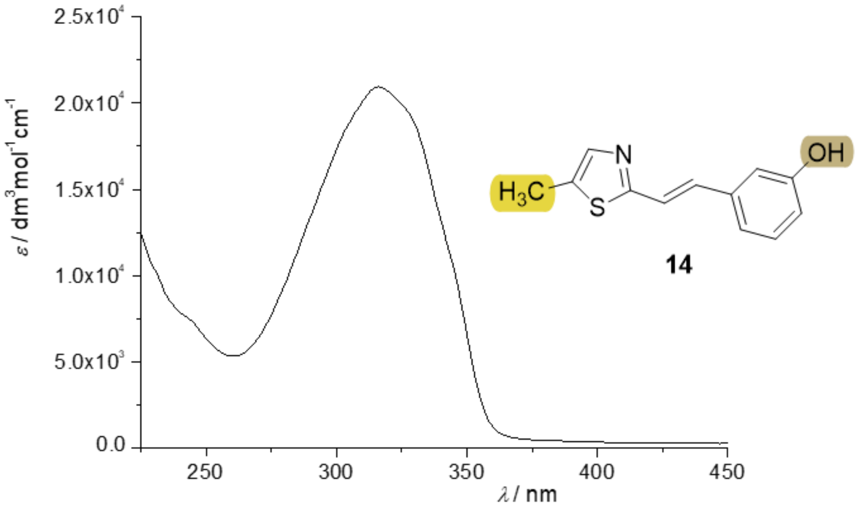


**Figure S75.** UV (ACN) spectrum of **14**.

## 4. Cartesian coordinates of docked ligands

**Cartesian coordinates of docked ligands**

Molecule **6** docked to AChE

| | | | |
|---|---|---|---|
| S | -14.706000 | -41.403000 | 23.885000 |
| C | -14.932000 | -42.979000 | 24.636000 |
| C | -15.977000 | -43.639000 | 24.023000 |
| H | -16.309000 | -44.648000 | 24.321000 |
| C | -16.587000 | -42.901000 | 22.972000 |
| H | -17.435000 | -43.284000 | 22.380000 |
| C | -16.023000 | -41.669000 | 22.763000 |
| C | -16.402000 | -40.630000 | 21.752000 |
| H | -17.241000 | -40.799000 | 21.037000 |
| H | -15.491000 | -40.375000 | 21.161000 |
| H | -16.598000 | -39.676000 | 22.294000 |
| C | -14.128000 | -43.478000 | 25.727000 |
| H | -13.129000 | -43.030000 | 25.861000 |
| C | -14.492000 | -44.447000 | 26.601000 |
| H | -15.497000 | -44.873000 | 26.440000 |
| C | -13.749000 | -45.009000 | 27.724000 |
| C | -12.516000 | -45.632000 | 27.442000 |
| H | -12.162000 | -45.672000 | 26.398000 |
| C | -11.735000 | -46.197000 | 28.442000 |
| Cl | -10.205000 | -46.961000 | 28.020000 |
| C | -12.147000 | -46.167000 | 29.772000 |
| H | -11.526000 | -46.616000 | 30.565000 |
| C | -13.361000 | -45.557000 | 30.077000 |
| H | -13.701000 | -45.525000 | 31.126000 |
| C | -14.157000 | -44.985000 | 29.082000 |
| O | -15.348000 | -44.388000 | 29.394000 |
| H | -15.839000 | -44.967000 | 29.998000 |

Molecule **5** docked to BChE

| | | | |
|---|---|---|---|
| C | 133.326000 | 117.379000 | 37.076000 |
| H | 133.541000 | 117.497000 | 36.001000 |
| C | 133.127000 | 116.154000 | 37.674000 |
| S | 132.802000 | 116.371000 | 39.390000 |
| C | 132.951000 | 118.112000 | 39.266000 |
| C | 133.229000 | 118.479000 | 37.975000 |
| H | 133.365000 | 119.528000 | 37.662000 |
| C | 132.776000 | 118.990000 | 40.468000 |
| H | 132.550000 | 118.691000 | 41.518000 |
| H | 131.985000 | 119.725000 | 40.189000 |
| H | 133.703000 | 119.607000 | 40.525000 |
| C | 133.170000 | 114.863000 | 37.023000 |
| H | 133.446000 | 114.000000 | 37.652000 |

| | | | |
|---|---|---|---|
| C | 132.905000 | 114.607000 | 35.718000 |
| H | 132.644000 | 115.477000 | 35.092000 |
| C | 132.926000 | 113.305000 | 35.045000 |
| C | 132.603000 | 113.123000 | 33.679000 |
| C | 132.666000 | 111.856000 | 33.095000 |
| H | 132.419000 | 111.736000 | 32.027000 |
| C | 133.037000 | 110.744000 | 33.844000 |
| H | 133.108000 | 109.748000 | 33.376000 |
| C | 133.316000 | 110.916000 | 35.196000 |
| F | 133.657000 | 109.840000 | 35.942000 |
| C | 133.254000 | 112.161000 | 35.799000 |
| H | 133.463000 | 112.258000 | 36.878000 |
| O | 132.200000 | 114.153000 | 32.873000 |
| H | 131.277000 | 114.370000 | 33.094000 |

Molecule **8** docked to BChE

| | | | |
|---|---|---|---|
| C | 133.606000 | 116.497000 | 41.719000 |
| H | 133.877000 | 115.594000 | 42.291000 |
| C | 134.584000 | 117.089000 | 40.894000 |
| C | 134.236000 | 118.284000 | 40.223000 |
| C | 132.946000 | 118.807000 | 40.342000 |
| H | 132.679000 | 119.734000 | 39.807000 |
| C | 131.997000 | 118.168000 | 41.130000 |
| H | 130.978000 | 118.584000 | 41.195000 |
| C | 132.313000 | 117.001000 | 41.845000 |
| C | 131.283000 | 116.317000 | 42.713000 |
| H | 130.248000 | 116.720000 | 42.814000 |
| H | 131.712000 | 116.211000 | 43.737000 |
| H | 131.207000 | 115.254000 | 42.384000 |
| C | 135.905000 | 116.461000 | 40.801000 |
| H | 136.326000 | 116.359000 | 39.786000 |
| C | 136.671000 | 115.990000 | 41.817000 |
| H | 136.254000 | 116.077000 | 42.834000 |
| C | 137.981000 | 115.385000 | 41.699000 |
| S | 139.484000 | 116.250000 | 42.000000 |
| C | 140.450000 | 114.831000 | 41.652000 |
| C | 139.644000 | 113.774000 | 41.322000 |
| H | 140.035000 | 112.776000 | 41.063000 |
| C | 141.947000 | 114.877000 | 41.729000 |
| H | 142.562000 | 113.974000 | 41.507000 |
| H | 142.230000 | 115.249000 | 42.741000 |
| H | 142.302000 | 115.706000 | 41.074000 |
| C | 138.256000 | 114.083000 | 41.345000 |
| H | 137.471000 | 113.347000 | 41.102000 |
| O | 135.123000 | 118.990000 | 39.458000 |
| H | 134.857000 | 119.926000 | 39.472000 |

## 5. **Tables S1** and **S2**, free energies of binding obtained by docking

Table S1. Free energy of binding, $DG_{bind}$, estimated by molecular docking of molecule **6** into the active site of AChE (4EY7.pdb), along with the number of conformational clusters, distribution of conformations, and docking results of the reference compound donepezil and galantamine.

| Ligand | $DG_{bind}$/kcal mol$^{-1}$ | | Number of distinctive conformational clusters | Distribution of conformations within clusters with *n* > 1 (*n* = cluster population) |
|---|---|---|---|---|
| | lowest | highest | | |
| **6** | −8.28 | −8.06 | 2 | 20, 5 |
| Donepezil | −11.37 | −10.75 | 4 | 22 |
| Galantamine | −10.11 | −10.10 | 1 | 25 |

Table S2. Free energies of binding, $DG_{bind}$, obtained by molecular docking of listed molecules into the active site of BChE (1P0I.pdb), along with the number of conformational clusters, distribution of conformations, and docking results of the reference compounds donepezil and galantamine.

| Ligand | $DG_{bind}$/kcal mol$^{-1}$ | | Number of distinctive conformational clusters | Distribution of conformations within clusters with *n* > 1 (*n* = cluster population) |
|---|---|---|---|---|
| | lowest | highest | | |
| **5** | −7.09 | −7.01 | 2 | 22, 3 |
| **8** | −7.26 | −7.19 | 3 | 20, 2, 3 |
| Donepezil | −9.58 | −8.29 | 3 | 9, 8, 4 |
| Galantamine | −7.49 | −7.44 | 1 | 25 |

**6.** Docked molecule **6**

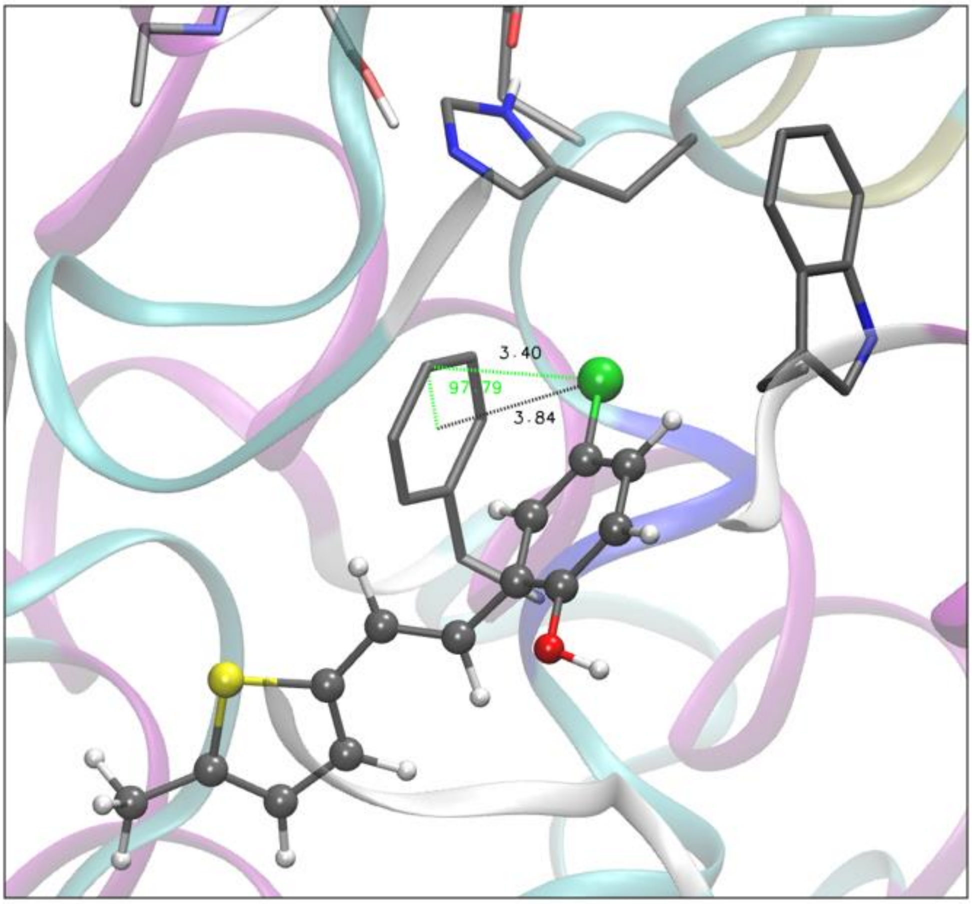


**Figure S76.** Molecule **6** docked into the active site of AChE, demonstrating an „edge-on" dispersive interaction between the ligand's chlorine atom and Phe338.
